# Discerning Seizure-Onset v. Propagation Zone: Pre-and-Post-Operative Resting-State fMRI Directionality and Boerwinkle Neuroplasticity Index

**DOI:** 10.1016/j.nicl.2022.103063

**Published:** 2022-05-28

**Authors:** Varina L. Boerwinkle, Bethany L. Sussman, Sarah N. Wyckoff, Iliana Manjón, Justin M. Fine, P. David Adelson

**Affiliations:** a Division of Pediatric Neurology, University of North Carolina, Dept. of Neurology, 170 Manning Dr, CB #7025, Chapel Hill, NC 25714, USA; b Division of Neuroscience, Barrow Neurological Institute at Phoenix Children’s Hospital, 1919 E. Thomas Rd, Ambulatory Building, Phoenix, AZ 85016, USA; c University of Arizona College of Medicine – Tucson, 1501 N. Campbell Ave, Tucson, AZ 85724, USA; d Department of Neuroscience, University of Minnesota, 321 Church St SE, Minneapolis, MN 55455, USA; e Division of Pediatric Neurosurgery, Barrow Neurological Institute at Phoenix Children’s Hospital, 1919 E. Thomas Rd, Phoenix, AZ 85016, USA

**Keywords:** BMA, Bayesian Model Averaging, BMR, Bayesian Model Reduction, BNI, Boerwinkle Neuroplasticity Index, BOLD, blood oxygen level dependent, CAT12, Computational Anatomy Toolbox version 12, DCM, dynamic causal modeling, DRE, drug resistant epilepsy, EEG, electroencephalography, EEG-fMRI, simultaneous EEG and functional MRI, GLM, general linear model, HH, hypothalamic hamartoma, ICA, independent component analysis, iEEG, intracranial EEG, IED, interictal epileptogenic discharges, IED-BOLD, simultaneous EEG-fMRI detected interictal epileptogenic discharges corresponding to blood oxygen level dependent rs-fMRI signal changes, LITT, laser interstitial thermal ablation therapy, MEG, magnetoencephalography, PCA, principal components analysis, PEB, Parametric Empirical Bayes, pZ, propagation zone, ROI, region of interest, rs-fMRI, resting state functional MRI, RSFC, (static) resting state functional connectivity, RSEC, resting state effective connectivity, SL, SearchLight, SOZ, seizure onset zone, Effective connectivity, Epilepsy surgery, Resting state functional MRI, Seizure, Surgical candidacy, Hypothalamic hamartoma

## Abstract

The goal of this study was to determine resting state fMRI (rs-fMRI) effective connectivity (RSEC) capacity, agnostic of epileptogenic events, in distinguishing seizure onset zones (SOZ) from propagation zones (pZ). Consecutive patients (2.1–18.2 years old), with epilepsy and hypothalamic hamartoma, pre-operative rs-fMRI-directed surgery, post-operative imaging, and Engel class I outcomes were collected. Cross-spectral dynamic causal modelling (DCM) was used to estimate RSEC between the ablated rs-fMRI-SOZ to its region of highest connectivity outside the HH, defined as the propagation zone (pZ). Pre-operatively, RSEC from the SOZ and PZ was expected to be positive (excitatory), and pZ to SOZ negative (inhibitory), and post-operatively to be either diminished or non-existent. Sensitivity, accuracy, positive predictive value were determined for node-to-node connections. A Parametric Empirical Bayes (PEB) group analysis on pre-operative data was performed to identify group effects and effects of Engel class outcome and age. Pre-operative RSEC strength was also evaluated for correlation with percent seizure frequency improvement, sex, and region of interest size. Of the SOZ’s RSEC, only 3.6% had no connection of significance to the pZ when patient models were individually reduced. Among remaining, 96% were in expected (excitatory signal found from SOZ → pZ and inhibitory signal found from pZ → SOZ) versus 3.6% reversed polarities. Both pre-operative polarity signals were equivalently as expected, with one false signal direction out of 26 each (3.7% total). Sensitivity of 95%, specificity 73%, accuracy of 88%, negative predictive value 88%, and positive predictive value of 88% in identifying and differentiating the SOZ and pZ. Groupwise PEB analysis confirmed SOZ → pZ EC was excitatory, and pZ → SOZ EC was inhibitory. Patients with better outcomes (Engel Ia vs. Ib) showed stronger inhibitory signal (pZ → SOZ). Age was negatively associated with absolute RSEC bidirectionally but had no relationship with Directionality SOZ identification performance. In an additional hierarchical PEB analysis identifying changes from pre-to-post surgery, SOZ → pZ modulation became less excitatory and pZ → SOZ modulation became less inhibitory. This study demonstrates the accuracy of Directionality to identify the origin of excitatory and inhibitory signal between the surgically confirmed SOZ and the region of hypothesized propagation zone in children with DRE due to a HH. Thus, this method validation study in a homogenous DRE population may have potential in narrowing the SOZ-candidates for epileptogenicity in other DRE populations and utility in other neurological disorders.

## Highlights

• Directionality differentiates the SOZ and pZ with 88% accuracy, 73% specificity, and 95% sensitivity.
• The Boerwinkle Neuroplasticity Index >70% is predictive of good outcomes.
• Signal from seizure onset zone to propagation zone is excitatory and signal from propagation zone to seizure onset zone is inhibitory.
• Greater inhibition from propagation zone is associated with better surgical outcome.
• Pre to post-operative SOZ and pZ modulation was diminished as expected.

## Graphical abstract

**Figure:**
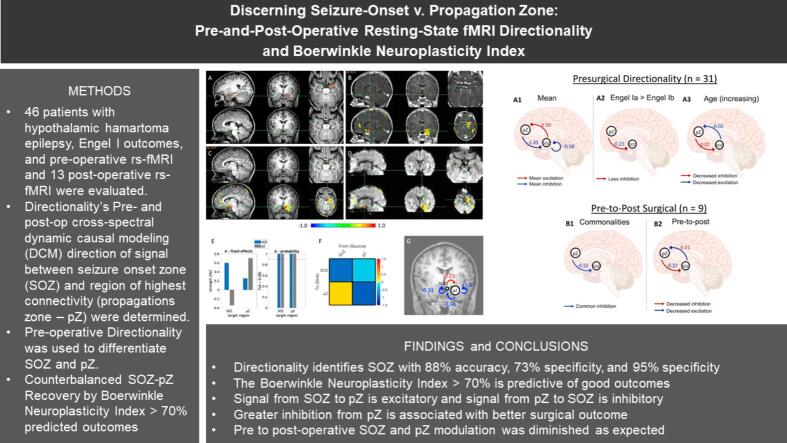

## 1 Introduction

The most effective and only known curative treatment for drug resistant epilepsy (DRE) is surgery to remove the seizure focus and or interrupt the epileptogenic network (Luders et al., 2006). The primary determinant of surgical candidacy and success is accurate localization of the seizure onset zone (SOZ) (West et al., 2019). However, current noninvasive SOZ localization methods still often depend on confirmation by intracranial electroencephalograph (iEEG), which is expensive, carry risks, and still only leads to 40–80% seizure-freedom when a seizure focus is “identified” (Tonini et al., 2004).

A recent *meta*-analysis shows improvement in noninvasive SOZ source localization over more standard methods has been demonstrated by static connectivity from resting state fMRI (rs-fMRI) via independent component analysis (ICA) (Chakraborty et al., 2020). Notably, rs-fMRI ICA-derived SOZ findings have not only been associated with iEEG, but also with increased surgical candidacy. Additionally, improved Engel outcomes, have been associated with resolution of post-operative rs-fMRI SOZ ICA networks (Boerwinkle et al., 2019a, Boerwinkle et al., 2020, Boerwinkle et al., 2017)***.*** However, after evaluating over 2000 rs-fMRI of individual studies with DRE using ICA for SOZ localization, a major weakness remains: this *static* network measure can identify more than one plausible SOZ candidate (Boerwinkle et al., 2019a, Boerwinkle et al., 2020, Boerwinkle et al., 2017).). Thus, there remains dependency on stereotactic iEEG to distinguish the best surgical target a concordance of data from rs-fMRI and other noninvasive methods.

The fundamental limitation of static rs-fMRI ICA is overcome to some extent by employing simultaneous EEG-fMRI, though this method only identifies the SOZ in a small proportion of cases, alone (Vaudano et al., 2021). The interictal epileptogenic discharges (IED) detected by EEG inform the time point at which pre-to-post blood oxygenation level dependent (BOLD) imaging maximal regions of interest (IED-BOLD ROI) are identified (Khoo et al., 2017). A possible advance on this method, subsequent dynamic causal modeling (DCM) of the IED-BOLD ROI, has *differentiated the epileptogenic driver from propagation regions in four cases as determined by good surgical outcomes* (Engel class I and II) (Engel, 1993, Vaudano et al., 2021). However, this limited series highlighted the dependency of EEG-fMRI on requires additional specialized equipment, EEG technician staff, and epileptiform events to occur during the scan; all of which are cumbersome features limiting clinical utility.

To promote advancement in this area, we designed a computational approach, Directionality, which employs cross-spectral DCM from *rs-fMRI alone, without electrophysiological information,* known as resting state effective connectivity (RSEC) helps to differentiate the driving SOZ region from propagation regions. These areas can be initially identified from the static rs-fMRI analysis. Directionality assumes SOZ are: First, (1) generators of excitatory (positive) signal toward regions of propagation; and secondly (2) receivers of inhibitory (negative) activity from the propagation node(s).

To determine if RSEC accurately distinguishes between SOZ and propagation zones (pZ), we selected a DRE population with homogenous and established SOZ localization, as well as prior surgically validated static rs-fMRI SOZ and pZ determined by the static rs-fMRI connectivity measure, SearchLight (SL) (Boerwinkle et al., 2018a). SL yields the Pearson Correlation of each HH voxel and the rest of the brain. SL in HH DRE was found to detect significant connectivity between the HH and the established regions of initial HH seizure propagation (thalamus, anterior cingulate, hippocampus, occipitotemporal junction, parahippocampal gyrus, amygdala, anterior operculum, nucleus accumbens, and caudate) reported by all other modalities (Boerwinkle et al., 2016, Regis et al., 2017).

DRE from our patients with hypothalamic hamartoma (HH-DRE) fills these criteria because (1) it is well-established that the HH is the primary seizure driver in HH-DRE; (2) the HH epileptogenic network dynamic follow the Papez circuit, as verified by prior iEEG, rs-fMRI by ICA and partial correlation (Boerwinkle et al., 2016) and SL (Boerwinkle et al., 2018a), and EEG-fMRI by DCM (Murta et al., 2012, Usami et al., 2016). Notably, the same pathway of seizure progression from the HH to the rest of the brain from iEEG was found by rs-fMRI static measures alone in HH, but only on a group level (Boerwinkle et al., 2016) inferring the potential success of SOZ-identifying information from rs-fMRI Directionality on an individual basis.

In those with additional post-operative rs-fMRI, we compared pre-to-post-operative rs-fMRI Directionality-derived Boerwinkle Neuroplasticity Indices (BNI), hypothesizing a >70% reduction in combined proportional SOZ->pZ excitation and pZ->SOZ inhibition. In this study, we tested our primary hypotheses that signal direction by Directionality and BNI threshold will distinguish the known HH’s SOZ from pZ in children with HH-DRE who underwent pre-operative rs-fMRI SL-guided laser interstitial thermal ablation therapy (LITT) and had Engel I surgical outcomes. We secondarily hypothesized that higher strength of signal from the identified region with negative signal (the pZ) and higher excitation from the region with positive signal (the SOZ) will correlate with seizure outcome. We evaluated the relationship between age, a surrogate marker for the length of time of DRE in this congenital rare epilepsy, hypothesizing that age would be associated with decreased improvement with surgery (Tonini et al., 2004), signal strength between these regions.

## 2 Methods

### 2.1 Participants

The local institutional review board granted approval for this study. Rs-fMRI became part of the standard preoperative evaluation for epilepsy surgery in 2012 and 2017 at Texas and Phoenix Children’s Hospitals, respectively, where the data was collected, therefore, no additional consent was deemed necessary for this retrospective rs-fMRI algorithm evaluation study.

Overall, there were 46 consecutive HH-DRE patients who underwent LITTs of pre-operative rs-fMRI surgical target, as in Boerwinkle et al. (2018a). Of these, 36 had Engel class 1 outcomes at least 12 months from surgery, meeting study criteria. Four total patients’ data were excluded: one due to file corruption and three from inadequate signal quality secondary to patient motion, yielding 31 total pre-operative full 600 vol acquisitions. Three of these patients, who had Engel 1b outcomes, subsequently had a second surgery, and both surgeries were included. One other pre-operative scan with only 300 volumes was included for pre-post rs-fMRI comparison.

Thirteen patients with available post-operative rs-fMRI (6 with 600 volumes and 7 with 300 volumes) at least 4 months from surgery and one year Engel I outcomes were similarly evaluated (included within the 31 full data sets and the one with only 300 pre-operative volumes). Thus, including a total of 32 pre-op and 13 post-op scans total (Table S1). Rs-fMRI SOZ ablation location was confirmed by two blinded study personnel based on visualization of the pre-operative SOZ and post-operative imaging, as previously described (Boerwinkle et al., 2018a) (see Table S2). The Directionality analyses were carried out by two rs-fMRI experts (BS and VB).

### 2.2 MRI data acquisition and processing

#### 2.2.1 MRI acquisition

Images were acquired on a 3 Tesla MRI (Ingenuity, Philips Medical Systems, Best, Netherlands) equipped with a 32-channel head coil. Rs-fMRI parameters included TR (repetition time) 2000 ms, TE (echo time) 30 ms, matrix size 80x80, flip angle 80, number of slices 46, slice thickness 3.4 mm with no gap, in plane resolution 3x3 mm, inter-leaved acquisition, and number of total volumes 600 (unless otherwise specified), in two 10-minute runs, totaling 20 min. For anatomical reference, a T1-weighted turbo field echo whole-brain sequence was obtained with the following parameters: TR 9 ms, TE 4 ms, flip angle 8, slice thickness 0.9 mm, and in-plane resolution 0.9 × 0.9 mm.

#### 2.2.2 MRI preprocessing

Using the program Statistical Parametric Mapping version 12 (Friston et al., 1994) (SPM12; www.fil.ion.ucl.ac.uk/spm, Wellcome Trust Centre for Neuroimaging, London, UK), in Matlab 2019b, the T1 image was resampled to 1x1x1 mm voxels, the origin was set at the anterior commissure and, if needed, minor adjustments were made to correct orientation (head-tilt). The T1 image was segmented using the Computational Anatomy Toolbox version 12.7 (CAT12) (Gaser and Dahnke, 2016) and the Automatic Anatomical Labelling Atlas 3 (AAL3) (Rolls et al., 2020) was registered into subject T1 space and masks for each of the hippocampi were generated.

For the fMRI data, the same pre-processing steps as prior work (Boerwinkle et al., 2019a, Boerwinkle et al., 2017) were applied by high-pass filtering the data to remove ultra-low-frequency non-neural artifacts and extract the grey matter voxel time-course while removing voxels in the cerebrospinal fluid and correcting for subject movement. Following ICA-based denoising (manual expert classification with removal of noise-based components in epilepsy (Boerwinkle et al., 2019a, Boerwinkle et al., 2017), CSF, and motion regression), the denoised functional data were re-aligned in SPM to register both of each subjects’ 10-minute rs-fMRI runs to each other. The mean functional image was segmented with unified segmentation. Using the bias-corrected mean functional image, the origin was set and orientation adjustments were made as described above for the T1. Functional images were co-registered (estimate) to T1 space in SPM using the bias-corrected mean functional image, with visual inspection.

#### 2.2.3 ROI selection

**Fig. 1:**
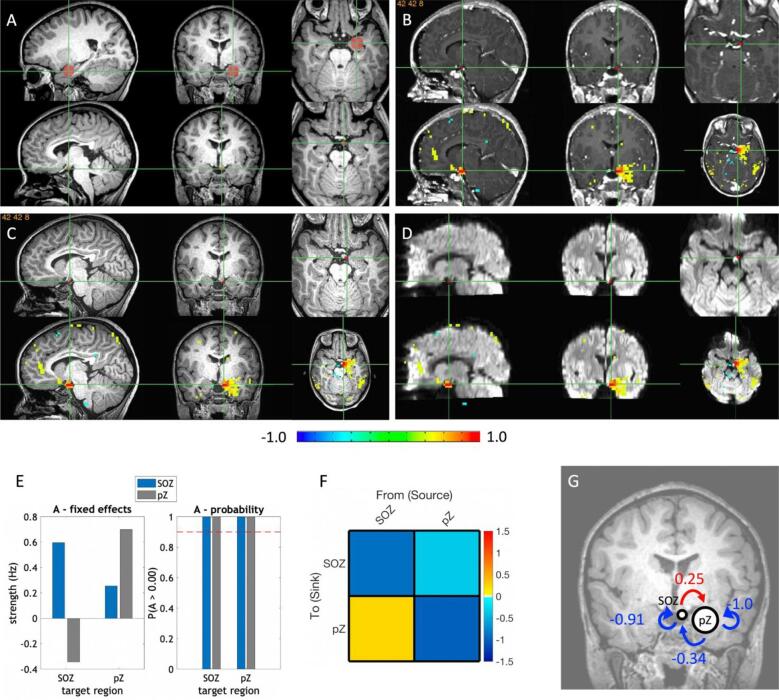
**Panel A-D Examples of SOZ and pZ selection.** Each set of six images is divided into two rows of sagittal, coronal, and axial images. **A.** Pre-operative T1W wherein row 1 is a left amygdala-hippocampal region pZ (red circle), and in row 2 is the SOZ within the HH, which is one voxel large. **B-D.** Post-operative post-contrast T1W, pre-operative T1W, and post-operative diffusion weighed images, respectively showing in row 1 same SOZ as in A, and row 2 includes the SL connectivity to the SOZ. These views allow visualization of the regions of the SOZ destroyed as either the post-contrast or the diffusion were positive in SOZ location and with connectivity to the pZ selected. The color bar reflects positive connectivity increasing from green to red, and negative in green to blue, signifying the connectivity values in the images above. **Panel E-G Individual DCM results**. **E.** Parameter estimates and their posterior probabilities of the fully specified DCM A matrix (intrinsic connectivity) after inversion using cross-spectral DCM. Target region is listed on the x-axis; connections from the SOZ (HH) are in blue and connections from the pZ are in grey. The left graph is the effect sizes, self-connections are shown in the log scaling value used to stabilize the model (conversion to Hz is −0.5 * exp(A)). The right bar chart shows the posterior probabilities of each estimated parameter. **F.** Adjacency matrix of the A matrix parameter estimates after optimization (exhaustive BMR followed by BMA). This matrix is thresholded to only include connections with a posterior probability >0.9. Self-connections have been converted from the log-scaling value to Hz. **G.** The values from panel C shown as a diagram superimposed on the patient’s coronal T1W image. Abbreviations: DCM: dynamic causal modeling; ROI: region of interest; pZ: propagation zone; SOZ: seizure onset zone; SL: SearchLight; HH: hypothalamic hamartoma.

The SOZ and pZ were located according to prior clinical SL results (Boerwinkle et al., 2018a, Boerwinkle et al., 2016, Murta et al., 2012, Usami et al., 2016), constrained by: (1) SOZ must be within the HH and in all cases the SOZ size was less than the size of the HH (from Boerwinkle et al, 2018a the total HH mean (SD) and range in size were 7.7 (5.4), 1.5–18.4 cm^3^). (2) SOZ must be located within the post-operative ablated portion of the HH that also preoperatively had the largest cluster with the highest correlation to the regions outside the HH in pZ candidate initial propagation region(s), determined by prior modalities, including the thalamus, anterior cingulate, hippocampus, occipitotemporal junction, parahippocampal gyrus, amygdala, anterior operculum, nucleus accumbens, and caudate, or the closest neighbor to one of these regions. Clusters covering pZ candidate regions with high positive (yellow–red) considered to have greater connectivity than negatively correlated regions (blue-green). Thus, borders of the SOZ and pZ were determined by those SL HH voxels within the HH meeting these criteria and the structure of the pZ candidate regions with no inclusion of possible contiguous high connectivity tissue by SL between them, if present. The pZ was either drawn manually or an AAL3 hippocampus mask. For example, in Fig. 1 the amygdala fits pZ criteria, whereas the occipital location does not. For pZ, the smallest regional sphere of grey matter thickness (narrowed to 1–2 gyri) within continuous single brain structure/region was selected, with exceptions as detailed in results.

Time series extraction from the SOZ and pZ were performed through a general linear model (GLM) of the rs-fMRI data. Given the prior noise correction, no additional noise correction regressors were used. Both rs-fMRI runs were entered as the same session. SPM concatenate (Casanova et al., 2007) was used to specify the boundary between the runs to correct for intensity differences between sessions. From the resulting SPM, a time series for each SOZ and pZ was extracted from the specified ROI masks by first thresholding to only include activity at p < 0.5 in the adjusted SPM, then computing the first eigenvariate of the region, thereby reducing the effective ROI size.

#### 2.2.4 DCM

##### 2.2.4.1 Model estimation

In overview, cross-spectral DCM (Friston et al., 2014) was used to model the regional time course signals, in a fully-connected model (meaning forward and backward between all nodes, and nodal self-connection), similar to previously described (Sussman et al., 2021). Cross-spectral DCM estimates parameters of auto- and cross-spectrum through multivariate auto-regression models of BOLD data generating estimated spectrums. As such, a two-node model was specified per case and steady-state spectral amplitude and phase representations of each node’s (SOZ or pZ) first principal component activity was obtained through Fourier transform. Variational Bayesian inversion was used to fit the differential equation connectivity model (Friston et al., 2003, Friston et al., 2014). Inversion involves fitting the DCM to maximize the likelihood of the model under prior parameter specifications. Model-fitting estimates parameters that describe the amplitude by frequency-spectral representation for each region. Each region’s local spectrum was modeled as a power law distribution with an amplitude and scale, the latter indicating the frequency by amplitude slope. Effective connectivity was derived through estimating the same parameters through frequency cross-spectrum between regions.

##### 2.2.4.2 Single subject model comparison

Our aim of identifying connections in this HH network was achieved by a Bayesian model reduction (BMR) and averaging approach (BMA). Specifically, we estimated the probability that every single model generated the data through its Bayesian furnished model evidence of the fully connected model (BMR). The model evidence was then used to weight the probability of each connectivity parameter in each fit model. These parameters were then averaged across all models to determine the statistically optimal estimate of local neural and regional connectivity effects (BMA). This approach allowed us to furnish a statistical reliability measure of estimated connectivity parameters for each tested model within a single subject, circumventing the need to compare models across subjects. A threshold of 0.9 was used for the posterior probability of each parameter for further analysis.

##### 2.2.4.3 Group model estimation and comparison

In addition to single-subject model comparison, we planned a group Parametric Empirical Bayes (PEB) analysis to perform group-based model reduction and comparison to identify mean group effects as well as investigate the group effects of Engel class outcome and age on parameter estimate size. For this, the fully connected individual DCMs were estimated, but then exhaustive Bayesian model reduction, comparison, and averaging were performed through a PEB paradigm (Friston et al., 2016, Friston et al., 2015, Zeidman et al., 2019). Exhaustive BMR was used because it is a data-driven strategy in an otherwise hypothesis driven technique. In the model, Engel class 1a outcome were coded as 1 and Engel class 1b outcome were coded as −1. The covariates were mean-centered and entered in the following order: Engel class outcome, age. Covariate effects of interest (posterior probability > 0.95) were entered into a leave-one-out (LOO) cross-validation analysis to examine their predictive ability. For discussion purposes, we focused on parameters with a posterior probability>0.95 (free energy).

##### 2.2.4.4 Post-operative rs-fMRI comparison and Boerwinkle Neuroplasticity Index

For the patients with pre- and post-operative rs-fMRI and ongoing brain activity in the ablated SOZ, we performed a 3-level hierarchical PEB analysis similar to Park et al. (2017). Briefly, the first level was session (fully connected DCMs separately inverted for pre and post rs-fMRI for each subject without model reduction). Next (second level) a PEB was run on the subject level (i.e., 9 PEBs), comparing the two (pre minus post) and evaluating commonalities and differences. Lastly (third level), A PEB of PEBs was run across this group, evaluating commonalities and differences from pre to post on a group level, then BMR with BMA was performed. We included parameters with a posterior probability >0.9 (free energy) in our discussion. In sum, each case was compared to itself in a repeated-measures fashion before being compared to the group.

Ablation heats the HH tissue either destroying it or rendering it viable but less functional. Thus, we expected the prior SOZ to pZ excitation to become no longer detectable or reduced below seizure generation threshold, with corresponding reductions in SOZ to pZ excitation and pZ to SOZ inhibition. Those SOZ to pZ connection changing direction from excitation to inhibition were considered reduced along the expected continuum, and correspondingly so for pZ to SOZ inhibition to excitation, as post-operative signal from pZ no longer needs to be inhibition for appropriate/expected signal counterbalance. To account for and capitalize on this dependency in counterbalanced SOZ-pZ signal phenomenon, we created the Boerwinkle Neuroplasticity Index (BNI). The BNI is derived from the proportional combined non-thresholded reductions in respective SOZ excitation and pZ inhibition (Table 2). These are the signal magnitude and sign values obtained from the individually inverted (non-reduced) pre and post ablation DCMs for each subject. We theorize the minimum BNI to achieve seizure freedom is >70%.

**Table 1:** Demographics and pZ location.

**N = 31**		
Sex (M:F)	20 M: 11F	
Handedness	16R: 7L: 8ND	
Age (Mean, SD)	(8.05, 5.18)	

**pZ**	**Left/Right/Bilateral**	**Total**
Amg-Hipp	3/7/0	10
Frontal	3/4/0	7
Temporal	3/3/0	6
Parietal	1/0/0	1
Occipital	0/1/0	1
Cingulate	0/0/1	1
Basal ganglia	3/0/0	3
Thalamus	1/0/0	1
Brainstem	1/0/0	1
**TOTAL**	**15/15/1**	**31**

Sex: M – male, F – female; Handedness: R – right, L – left, ND – non-dominant; SD – standard deviation; pZ – propagation zone, Amg-Hipp – amygdala-hippocampus region. Handedness was determined by epileptologists’ documentation in patient’s medical record.

**Table 2:** Pre- and post-operative directionality comparison signal magnitudes and Boerwinkle neuroplasticity index.

Measure	Subject data									*M*	*SD*
Subject (N = 9)	1	2	3	4	5	6	7	8	9		
Volumes	300	300	300	600	300	300	600	600	600		
Pre-op SOZ → pZ (Excitation)	0.14	0.28	0.10	0.50	0.08	0.41	0.21	0.04	0.23	0.22	0.16
Post-op SOZ → pZ (Excitation)	−0.59	0.08	0.08	−0.52	−0.51	−0.30	−0.54	0.27	0.38	−0.18	0.38
Reduced Excitation ^a^	1	1	1	1	1	1	1	0	0		
Became Inhibitory ^a^	1	0	0	1	1	1	1	0	0		
SOZ Recovery Index ^b^	512	71	22	203	768	172	360	−621	−66	158	391
Pre-op pZ → SOZ (Inhibition)	−0.03	−0.97	−0.84	−0.91	−0.40	−0.96	−1.18	−0.20	−1.09	−0.73	0.41
Post-op pZ → SOZ (Inhibition)	0.40	−0.26	−0.30	0.47	0.15	−0.01	0.02	−0.94	−1.50	−0.22	0.64
Reduced Inhibition ^a^	1	1	1	1	1	1	1	0	0		
Became Excitatory ^a^	1	0	0	1	1	0	1	0	0		
pZ Recovery Index ^c^	1239	73	64	152	138	99	102	−359	−38	163	433
Boerwinkle Neuroplasticity Index ^d^	1751	144	86	354	906	271	462	−980	−104	321	739

Note. pZ = propagation zone; SOZ = seizure onset zone. ^a^ 0 = no, 1 = yes. ^b^ SOZ Recovery Index = ^c^ pZ Recovery Index = ^d^ Boerwinkle Neuroplasticity Index = SOZ + pZ Recovery Index.

### 2.3 Statistical analyses

#### 2.3.1 Demographics and comparison of single subject model results

Baseline demographics and clinical factors were summarized using count and percent for categorical variables and the mean and standard deviation for quantitative measures. Descriptive SOZ and pZ locations were quantified. Age was used as surrogate total time of epilepsy condition in HH, given the congenital lesion of HH and initiation of gelastic seizures frequently occurs in the neonatal and first year of life, but is often has significant delay in seizure recognition (Harvey and Freeman, 2007).

Sensitivity, specificity, accuracy, and negative and positive predictive value were determined by Directionality’s identification of the SOZ and propagation zones in individuals’ models from the single subject model estimation and comparison. Pre-operative full data sets true positive (TP), true negative (TN), false positive (FP), and false negative (FN) were determined on a connection basis. Post-operatively, the BNI > 70% (threshold chosen per hypothesis) indicated sub-seizure threshold activity qualifying as TN. Those SOZ surgically obliterated resulting in total signal loss, also qualified as TN (detailed in Table 3). Pre-operative SOZ and pZ connections counted as 1 each; Post-operative connections were combined into a single marker, multiplied by two to account effectively for the weight of both connections.

**Table 3:** Contingency table of directionality results and signal characterization.

		*True condition*										Total	Measures (95% CI)
***Predicted Condition***		***Positive***					***Negative***						**Positive Predictive Value**
	***Positive***	**TP Aggregated Connections (n = 55)**					**FP Aggregated Connections (n = 8)**					**63**	88% (78–93%)
		Op	SOZ → pZ	pZ → SOZ	SOZ-pZ BNI	n	Op	SOZ → pZ	pZ → SOZ	SOZ-pZ BNI	n		
		Pre	Excitatory			27	Pre	Inhibitory			1		**Negative Predictive Value**
		Pre		Inhibitory		28	Pre		Excitatory		1		88% (70–96%)
							Pre	Detected, n.s.			1		
							Pre		Detected, n.s.		1		**Sensitivity**
							Post			BNI < 70%	2*2		95% (86–99%)
	***Negative***	**FN Aggregated Connections (n = 3)**					**TN Aggregated Connections (n = 22)**					**25**	
		Op	SOZ → pZ	pZ → SOZ	SOZ-pZ BNI	n	Op	SOZ → pZ	pZ → SOZ	SOZ-pZ BNI	n		**Specificity**
		Pre	Detected, n.s.			2	Post			BNI > 70%	7*2		73% (54–88%)
		Pre		Detected, n.s.		1	Post			SOZ Obliterated, No Signal	4*2		
													**Accuracy**
***Total***		**58**					**30**					**88**	88% (79–94%)

**Abbreviations:** n: number; n.s., non-significant; BNI: Boerwinkle Neuroplasticity Index; Op: Operative phase, pre- post-; pZ: propagation zone; SOZ: seizure onset zone; TP: true positive; FP: false positive; FN: false negative; TN: true negative; **Formulas:** Accuracy: TP + TN/(TP + TN + FP + FN) × 100; Negative Predictive Value: TN/(TN + FN) × 100; Positive Predictive Value: TP/(TP + FP) × 100; Sensitivity: TP/(TP + FN) × 100; Specificity: TN/(TN + FP) × 100.

The proportion of agreement between pre-operative Directionality and surgical outcome with 95% binomial exact confidence interval (CI) was calculated by node-to-node connection (see pre-operative values in Table 3 contingency table). The agreement of pre-operative Directionality and surgical outcome was assessed using the prevalence-adjusted bias-adjusted kappa (PABAK) (Byrt et al., 1993) in R 3.5.2 (R Core Team, 2017) with epiR v2.0.4 (Stevenson and Sergeant, 2021) package. A groupwise effective connectivity PEB analysis was performed as described above.

#### 2.3.2 Bias

To avoid bias we also explored whether PEB results were likely related to additional clinical, demographic, or analysis-related (e.g., SOZ and pZ size) variables, we also planned a series of tests as follows: To further explore if PEB results are explainable by clinical and demographic characteristics, we correlated age with SOZ size, pZ size, pre-operative seizure frequency, and age. Also, to ensure that SOZ and pZ size was not associated with DCM outcomes, we correlated the SOZ and pZ size with baseline (A matrix) connectivity parameters from the PEB analysis. Finally, although it was not included in the PEB model, we examined whether sex was associated with baseline parameter estimates from the PEB given sex-based seizure-network related differences (Boerwinkle et al., 2019a), as well as pre-to-post operative seizure percent improvement. Independent sample comparisons of continuous variables were tested using two-sided t-tests or Mann-Whitney tests in cases of violations of assumptions of normal data. Correlations were tested using two-sided Pearson’s r tests. Bonferroni corrections were used. All confounder analysis tests were performed with JASP Statistics v0.16. (JASP Team, 2021).

## 3 Results

### 3.1 Descriptive results

Individual patient clinical characteristics are summarized in Table 1 and expanded on in Table S1. Of the full data pre-op 31 patients, the mean age was 8.1 years (standard deviation (SD) of 5.2 years, range of 2.1–18.2 years), with a male to female ratio of 20:11. The pZs were both cortical and subcortical (27 cortical:4 subcortical), and in greater proportion in the frontal and temporal lobes (Table 1). The median size of the SOZ mask was one 3x3x3.4 mm voxel [interquartile range (IQR): 1–1; range: 1–11] and the median size of the pZ mask was sixty-five 3x3x3.4 mm voxels [IQR: 8–181.3; range: 1–227]. Of the pZ, 26 were spherical, 4 fit a structure’s mask (hippocampus/putamen), and 1 was irregularly shaped to a region. Regarding pZ brain sub-structure conformity largest dimension, 18 were one grey matter thickness with the exception that the hippocampus was considered 1 width, 10 were 1–2 contiguous gyri, and 3 were regionally larger. The pZ mean longest dimension was 7.6 mm, SD 4.5, with range of 1–25 mm.

Each pZ time series was determined by its first eigenvector, resulting in a representative reduced volume with mean of 3.09 cm^3^, SD 3.11 cm^3^, range 0.03–8.45 cm^3^. This first eigenvector pZ size was not correlated to strength or polarity of connections, however the analysis was not designed to determine the scale of connectivity metric changes as regional ROI size varies. There was inadequate data to determine if a relationship between shape and connectivity metrics existed.

### 3.2 Comparisons with individually estimated and reduced models

**Fig. 2:**
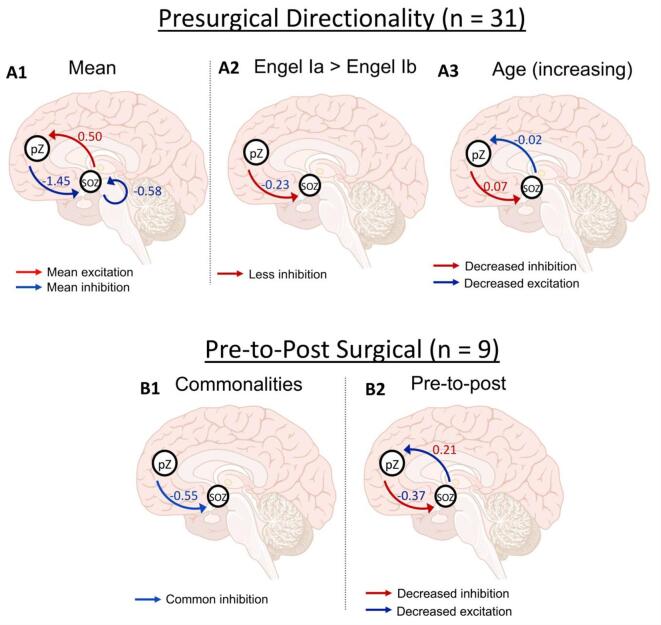
**A1**. Group baseline connections between the SOZ and pZ thresholded at 0.95 posterior probability after exhaustive Bayesian Model Reduction (BMR) and Bayesian Model Averaging (BMA). The location of the pZ varied by subject. Red arrows and values are excitatory modulation, blue arrows and values are inhibitory modulation. **A2-A3.** Group connections associated with Engel outcome and age thresholded at 0.95 posterior probability. Blue arrow/value indicates a negative correlation and a red arrow/value indicates a positive correlation with age. **A2.** Engel class Ia outcomes were coded with a higher value than Engel class Ib outcomes, thus, the blue arrow in Panel A indicates that patients with better one-year outcomes (Engel class Ia) showed more inhibitory modulation from the pZ to SOZ. A red arrow indicates less inhibition. **A3.** Older patients show decreased inhibition from the pZ to SOZ and decreased excitation from the SOZ to pZ, thus, overall, absolute strength of these parameter effect sizes decreased with age. **B1-B2.** Pre-to-post surgical changes in modulation thresholded at 0.9 posterior probability. **B1.** Common between pre and post ablation was inhibitory modulation from pZ to SOZ (blue arrow). **B2.** From pre to post ablation (pre > post), there was decrease in inhibitory signal from pZ to SOZ and a decrease in excitatory signal from SOZ to pZ (red arrows indicate decreased inhibition, blue arrows indicate decreased excitation).

Results from the individual DCM estimation, model reduction, and Bayesian model averaging for an example subject are shown in Fig. 2, and all subjects in Table S2**.**

Of the pre-operative SOZ effective connectivity, 9.6% had no significant connection. Of the remainder with significant connections, 96% and 3.6% were in the expected and reversed directions, respectively. Pre-operative positive and negative polarity signals were nearly equivalent as expected between the SOZ and pZ.

Of the 13 with post-operative rs-fMRI, 12 were part of the original 31 patients with full 600 vol data, and an additional one with only 300 pre-operative rs-fMRI volumes. SOZ and pZ pre to post op images with respective DCM matrices are illustrated in Table S3. In four, the SOZ was ablated to the point of rs-fMRI signal obliteration at the SOZ, with the remaining nine having adequate signal for DCM. In comparing pre- to post- op SOZ and pZ signal (Table 2), notably the expected nearly proportional decrement in respective excitation and inhibition from the SOZ and pZ occurred in 7 of the 9. Further of the five SOZ with signal inversion from excitatory to inhibitory, 4 conversely also had pZ signal swap from inhibitory to excitatory. The two of the nine whose SOZ excitatory signal increased, also had increasing pZ inhibitory signal. Thus, of the 13 total, 85% SOZ-pZ pairs neuroplasticity pre- to post-operative was as expected, and all pairs SOZ-pZ interaction indicated mutual proportional inverse modulation pre to post operatively.

Regarding discernment of SOZ from pZ, preoperative Directionality with pre-to-post operative Boerwinkle Neuroplasticity Index yielded the SOZ-pZ discerning values (Table 3). The PABAK estimate was 0.86 (95% CI: 0.65–0.96), indicating substantial agreement between pre-operative Directionality and surgical outcome.

### 3.3 Parametric Empirical Bayes group analysis results

The BMA group model is shown in Fig. 2**A1**. The connection from the SOZ to the pZ was excitatory and the connection from the pZ → SOZ was inhibitory. The SOZ self-connection was also present (inhibitory). The pZ self-connection was pruned during BMR.

**Supplementary figure 1:**
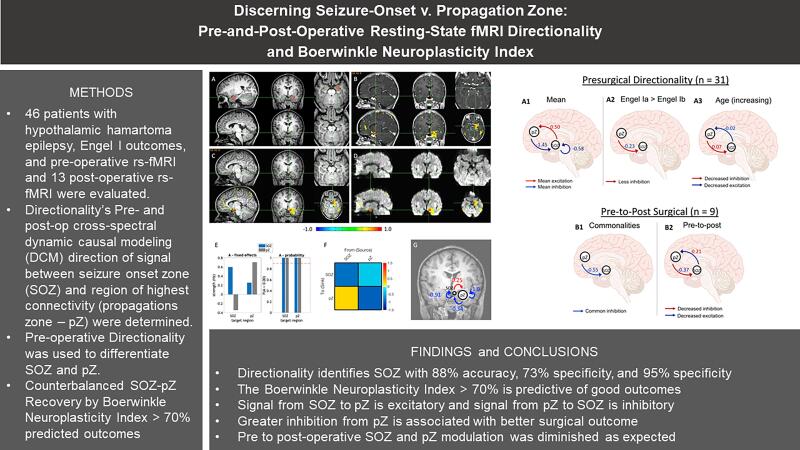

Regarding behavior correlations, Engel class outcome was associated with signal strength ***from the pZ to the SOZ*** (Fig. 2**A2**). Patients with a better Engel class outcome (1a) showed greater inhibitory signal from the pZ to the SOZ **(**Fig. 2**A2)**. However, this difference, however, was not significant with LOO cross-validation (r(29) = −0.05, *P* = 0.6; Figure S1**A**). Age was also associated with between-node connections; age was positively correlated with signal from the pZ → SOZ and negatively correlated with signal from the SOZ → pZ (Fig. 2A3). Neither of these correlations were significant with LOO cross-validation r(29) = 0.06, *P* = 0.38 and r(29) = 0.28, *P* = 0.07, respectively (Figure S1**B-C**). Self-connections were not related to Engel outcome or age.

#### 3.3.1 Parametric Empirical Bayes Pre-post group analysis results

The BMA pre-to-post ablation results are shown in Fig. 2B1-2. From pre-to-post ablation, the only connection in common was pZ → SOZ inhibitory signal (Fig. 2**B1**). However, from pre-to-post ablation, pZ → SOZ showed decreased inhibition and SOZ → pZ showed decreased excitation (Fig. 2**B2**), as expected. Self-connections were not changed by ablation.

### 3.4 Bias comparisons results

Mann-Whitney test results were not significant for differences between Engel outcomes and patient variable of pre-operative seizure rate, SOZ, and pZ size (Table S4). Further, age at scan was not correlated with SOZ size, pZ size, seizure frequency, nor seizure frequency improvement (%), nor were SOZ or pZ size correlated with A matrix parameter estimates from the PEB analysis (Table S5). Student t-tests were not significant for sex differences for A matrix parameter estimates from the PEB analysis and a Mann-Whitney test was not significant for sex differences in seizure frequency improvement (%) **(**Table S5**).**

## 4 Discussion

This is the first reported DCM analysis of rs-fMRI, performed independent of electrophysiological data, to demonstrate the capacity of RSEC alone, via Directionality and BNI, to differentiate SOZ from the region of propagation with sensitivity of 95%, specificity 73%, accuracy of 88%, negative predictive value 88%, and positive predictive value of 88% in identifying and differentiating the SOZ and pZ This is also the first study to demonstrate that cross-spectral DCM of rs-fMRI yields the excitatory signal direction from SOZ to a region of propagation and inhibitory signal from the region of propagation back to the SOZ, on both in the individual and group level. Before application to the more heterogeneously localized SOZ of the general DRE population, it was necessary to perform this analysis in such a homogenous and well established SOZ location of the HH to understand the potential of this tool for narrowing ROI for SOZ location determination. Importantly, polarity of SOZ and pZ connections pre-operatively were *consistent in both individual and group analyses,* increasing confidence in individual application, similar to prior RSFC (Boerwinkle et al., 2019b, Boerwinkle et al., 2018a, Boerwinkle et al., 2017, Boerwinkle et al., 2016) and RSEC work (Vaudano et al., 2021).

Our current results show some differences in terms of polarity from prior studies. For example, we show that the pre-operative connection from pZ → SOZ is overwhelmingly negative; however, pZ → SOZ connections in prior event-based DCM studies in have been mostly reported positive (Hamandi et al., 2008, Murta et al., 2012, Vaudano et al., 2013) with one study showing a mix (Vaudano et al., 2013). Thus, our results show greater consistency of A matrix connection polarities. This difference may be due to the number of nodes modeled, differences in SOZ or pZ specification, patient effects (all of our patients had Engel class I outcomes), or potentially more likely – our model did not include direct inputs or modulatory influences during captured explicit epileptiform activity and used cross-spectra (second-order statistic) instead of time-varying fluctuations (time series). As such, we also assume that the state of the relationship between nodes in the current study is modelling the basal state of the relationship between SOZ and pZ without placing temporal emphasis on ictal or inter-ictal events. This may be different from their relationship during these events, thus contributing to differences seen in our analysis. However, the differences could also be related to volatility of nodal relationship during epileptiform activity, as nodal strength and polarity inconsistencies in prior EEG-fMRI are prevalent (David et al., 2008, Murta et al., 2012, Vaudano et al., 2013), whereas Directionality was highly consistent.

Also consistent for Directionality was network architecture. Bi-directional pre-operative connections between SOZ and pZ had overwhelmingly high probability, which is not dissimilar to previous time-locked DCM studies that compared models with and without bidirectional connections along propagation pathways (Murta et al., 2012, Warren et al., 2019). Thus, our results imply that both SOZ → pZ and pZ → SOZ connections in baseline epileptogenic networks are relevant and, in a two-node model, can be identified with exhaustive BMR rather than specifying models for BMR.

Both pre-operative groupwise covariates evaluated were revealing. Engel class Ia versus Ib outcomes showed greater inhibitory signal from pZ → SOZ. Assuming that inhibitory pZ → SOZ signal is suppressive, then this effect may indicate that not only is SOZ ablation needed for good outcome, but also a stronger pre-operative pZ inhibition encourages possible residual SOZ activity to remain below the seizure threshold. ***Notably, the potential of measuring a precision network-dependent factor of pZ inhibition to predict seizure outcome from rs-fMRI alone is novel.*** Further work is indicated with additional surgical outcomes, as, despite very strong posterior evidence, LOO cross-validation results were not significant. Likely, broader Engel class I-IV outcomes would clarify if the pZ inhibition strength is associated with surgical outcomes.

Absolute signal strength emitted from SOZ and pZ was smaller as age increased. However, these associations did not survive LOO cross-validation, limiting current predicative power of directional signal strength. Decreasing excitation from SOZ → pZ with age is novel, though counterintuitive, given that strength of aberrant signal is expected to increase with disease length. However, recent DCM of epileptiform propagation pathways of Lennox-Gastaut Syndrome also showed reduction with age (Warren et al., 2019). This being noted, the strength difference did not have an effect on SOZ detection yield in this group. Further, the strength difference over age is relatively small, thus not expected to have an effect on SOZ detection in other DRE populations.

Importantly, Directionality showed differences pre-to-post ablation. Specifically, in a sub-group with Engel Ia outcomes and a post-operative rs-fMRI, SOZ → pZ became less excitatory and pZ → SOZ became less inhibitory. While this was shown in a small subgroup, the results highlight Directionality’s ability to detect RSEC differences between the same (pre-operative) seizure network locations when seizure freedom is achieved. Such repeat measures in serially in the same patients pre- to post-treatment serve to validate RSEC. In all but one patient, SOZ → pZ signal became less excitatory, often becoming inhibitory or not showing appreciable modulation (in fact, a time-series was no longer able to be extracted from the ablated tissue in four patients). Similar to our results, another study using a hierarchical PEB analysis to identify RSEC changes after thalamotomy in essential tremor found a change in network dynamics after surgery (Park et al., 2017). As such, our results add strength to the assertion that aberrant directional network dynamics can be detected with RSEC, as can changes associated with clinical improvement after surgical intervention.

To the author’s knowledge, this is the first multisubject study of RSEC of epileptogenic networks to demonstrate pre-to-post operative changes with Engel I outcomes. Vaudano et al. (2013) recently depicted pre and post-surgical EEG-fMRI (epileptogenic activity) with sub-optimal surgical outcomes in a single case. In this case, the region that DCM retrospectively suggested was the SOZ was not removed and there were suboptimal outcomes. While a post-operative DCM was not performed, they did perform post-operative EEG-fMRI and intracranial EEG. In these tests EEG-fMRI showed fMRI activation in both the (pre-operative) DCM SOZ and pZ during IED/seizure and intracranial EEG SOZ was concordant with the pre-operative DCM SOZ (Vaudano et al., 2013). In contrast, in our study with patients with Engel I outcomes and the DCM SOZ ablated, there were clear corresponding changes from pre-to-post-operative RSEC. While we do not have Engel II-IV outcomes in this study, taken together, this suggests that the RSEC changes in the pre-post subgroup are most likely reflective of their seizure outcomes.

Engel outcomes and pre-operative seizure frequency and SOZ and pZ size were unrelated. Further, age, SOZ and pZ size, and pre-operative seizure frequency were unrelated to each other. The lack of relationship between surgical outcome and pre-operative seizure frequency and region size, as well as between variables, make it unlikely that these factors biased Directionality. RSEC strength correlated with Engel outcome, but not seizure frequency nor reduction. Thus, severity of epilepsy also does not appear to influence the applicability of Directionality.

Our hypothesis that, from rs-fMRI alone, intrinsic modulation from the SOZ is excitatory and modulation from the pZ is inhibitory is supported by our results. We did not time lock or model around ictal onset or known periods of interictal discharge (Daunizeau et al., 2012, Hamandi et al., 2008, Klamer et al., 2018, Klamer et al., 2015, Murta et al., 2012, Vaudano et al., 2013, Vaudano et al., 2009, Vaudano et al., 2021, Warren et al., 2019). Instead, we assumed, that in DRE, pathological networks are present in resting-state networks, irrespective of and independent of need for capture of epileptogenic events. This has been supported in previous studies using RSFC to reliably identify the (static) spatial locations of epileptogenic networks (but not the direction of signal propagation) between regions in these networks without time-locking to epileptogenic activity (Boerwinkle et al., 2018a, Boerwinkle et al., 2017, Boerwinkle et al., 2018b, Boerwinkle et al., 2016).

A network characterizing modality is valuable if unique and clinically relevant information is discovered. Static rs-fMRI has already proven itself by these standards without anchoring to simultaneous EEG (Boerwinkle et al., 2019a, Boerwinkle et al., 2018a, Boerwinkle et al., 2017, Boerwinkle et al., 2021, Boerwinkle et al., 2016, Chakraborty et al., 2020, Desai et al., 2018). Effective connectivity could prove similarly useful.

While it is possible to use methodologies to know whether patients were seizing in the scanner or would have shown concurrent epileptiform activity, the consistency of the results herein is a strength that implies that, in this population, such knowledge may not be necessary to model seizure propagation direction but requires further study in a larger population of these patients who had poor outcomes and see if the model remains consistent.

Using EEG-fMRI to guide DCM, Vaudano et al. (2021) found that all 4 patients whose DCM indicated SOZ was concordant with the clinical SOZ and surgically-targeted had good clinical outcomes, though this subsample was 14.3% (4/28) surgically-validated clinical yield of all patients who underwent EEG-fMRI. A broad summary of methodological differences between the current study and prior studies is detailed in Table 4.

**Table 4:** Comparison of methods in prior and current work.

Method differences	Prior work (EEG-fMRI)	Current study (rs-fMRI)	Comment
ROI selection	- Requires simultaneous EEG-fMRI, clinically cumbersome - Requires epileptic discharge (interictal/ictal epileptic activity) - ROI specification guided by identifying BOLD response associated with epileptogenic activity - ROI specification has also incorporated information from MEG (Klamer et al., 2015) and tractography (Hamandi et al., 2008)	- Unimodal rs-fMRI, EEG not needed - Static RSFC (ICA, SL), guides ROI specification as cross-spectral DCM relies on generalized measures of RSFC (Friston et al., 2014) making RSFC an appropriate basis for ROI selection - No requirement for capture of epileptiform activity	- Both require ROI selection method
Assumptions about epileptogenic drivers from subtype of timed events	- GPFA (Warren et al., 2019) - IED (Hamandi et al., 2008, Klamer et al., 2018, Vaudano et al., 2013, Vaudano et al., 2009, Vaudano et al., 2021) - ictal (David et al., 2008, Klamer et al., 2015, Murta et al., 2012)	- None	- EEG-fMRI assumes captured events indicate accurate direction of signal to identify SOZ. - rs-fMRI alone assumes excitation from SOZ and inhibition from pZ will be consistent and accurate to determine SOZ
DCM method subtype	- Deterministic timeseries (David et al., 2008, Hamandi et al., 2008, Klamer et al., 2018, Klamer et al., 2015, Murta et al., 2012, Vaudano et al., 2013, Vaudano et al., 2009, Vaudano et al., 2021, Warren et al., 2019) - Stochastic, with event specification (Daunizeau et al., 2012)	- Cross-spectral	
DCM matrices specified	- A matrix (baseline (intrinsic) connectivity) - B matrix (modulatory (context/edge) inputs) - C matrix (driving (direct/node) inputs) - D matrix (intrinsic gating)	- A matrix (baseline (intrinsic) connectivity)	- Matrices are dependent on inputs available, B-D matrices are related to extrinsic timed events, whereas the A matrix is from intrinsic (nonevent related) brain activity
Information that can be gleaned from each DCM method subtype	-Able to explicitly model SOZ by specifying direct/driving input to nodes and compare likelihood of driving input location (e.g., whether driving input during ictal/interictal event is to node A or B) and explicitly model modulation of connection during ictal/interictal event (e.g. whether modulation is to the A → B connection or the B → A connection) - EEG - BOLD events can be compared	- Can only use A matrix (intrinsic connections), does not test hypotheses about specific timed modulation of connections or direct input to nodes	- EEG-fMRI allows observation of timed signal transmission to determine node order
Model selection	- Generally, hypothesis driven model specification with FFX comparison - BPA	- Exhaustive BMR with BMA	- rs-fMRI utilizes a data-driven model, wherein no assumption of relationship between nodes is made. - BMR is less computationally intensive nested model estimation and comparison scheme than separately estimating each model
Evaluation of excitation in SOZ identification	- Yes, reported single patient validated with iEEG (Vaudano et al., 2013), Single patient with EEG-fMRI (Hamandi et al., 2008, Murta et al., 2012)	- Yes, 96% of HH SOZ with excitation to pZ	- Corroboratory.
Evaluation of inhibition in SOZ identification	- Reported but not interpreted as meaningful (Vaudano et al., 2013)	- Yes, identifies inhibition in A matrix as most common ‘feedback’ from pZ to the SOZ	- rs-fMRI alone offers differentiation of SOZ and pZ through inhibition as coming from the pZ as opposed to the SOZ
Validated by surgical outcomes	- Largest study N = 10, 6 with surgical outcome (Vaudano et al., 2021)	- Current study (N = 31)	- Most studies are without comparison to surgical outcomes

Abbreviations: SL: SearchLight; ICA: independent components analysis; IED: interictal epileptic discharge; EEG: electroencephalography; FFX: fixed effects; GPFA: generalized paroxysmal fast activity; BPA: Bayesian Parameter Averaging; BMA: Bayesian Model Averaging; RSFC: resting state functional connectivity.

### 4.1 Limitations

Because all patients included by study design had good outcomes, it is not known how Directionality will perform in the broader outcome nor heterogenous DRE cause population. Although this study used a homogenous group and only two nodes, it provides a foundational basis for expansion to more complex patients and models. Our method of SOZ and pZ selection does not preclude the possibility of additional pZs, that any given pZ is the ‘first’ pZ from the HH-SOZ, nor a ‘longer’ propagation pathway. Since patients with HH generally do not receive iEEG for surgical planning, it is not possible to determine the absolute truth of the pZ. However, since all HH-SOZs were selected to be within both the area of surgical destruction that lead to increased or total seizure freedom and within a location determined by SL to have high RSFC outside of the HH, and there were changes in the modulatory relationships between the selected SOZ and pZ after seizure freedom after SOZ destruction, we are confident that SOZ selection is accurate.

### 4.2 Future directions

Noninvasive characterization of regional excitation/inhibition relationships may prove clinically impactful in other neurological disorders amenable to precision-based network-targeted interventions such as in the broader DRE population, and possibly movement (Sussman et al., 2021), neurodegenerative, and neuro-psychiatric disorders. The current models depict seizure propagation pathways in two node models where the SOZ is known. Future directions should also investigate patients with suboptimal surgical outcomes as well as other epilepsies with other etiologies, models with more nodes, and cross-modality validation such as by EEG-fMRI (Iannotti et al., 2016) and ultimately against prospective surgical outcomes.

## 5 Conclusions

This study demonstrates sensitivity of 95%, specificity 73%, accuracy of 88%, NPV 88%, and PPV of 88% of Directionality to identify the origin of excitatory and inhibitory signal between SOZ and the hypothesized pZ in children with HH-DRE. This method validation study in a homogenous population with known SOZ location and surgical outcomes may be helpful in narrowing the SOZ in regions of suspicion for epileptogenicity.

## Data Availability Statement

The data made available under institutional IRB approval that supports the findings of this study are available in the Supplementary Material of this article.

## Funding

This research did not receive any specific grant from funding agencies in the public, commercial, or not-for-profit sectors.

### CRediT authorship contribution statement

**Varina L. Boerwinkle:** Conceptualization, Methodology, Validation, Formal analysis, Investigation, Resources, Writing – original draft, Writing – review & editing, Visualization, Supervision, Project administration. **Bethany L. Sussman:** Methodology, Validation, Formal analysis, Investigation, Writing – original draft, Writing – review & editing, Visualization. **Sarah N. Wyckoff:** Investigation, Formal analysis, Data curation, Writing – review & editing. **Iliana Manjón:** Visualization, Writing – review & editing. **Justin M. Fine:** Writing – review & editing. **P. David Adelson:** Writing – review & editing.

## Declaration of Competing Interest

The authors declare that they have no known competing financial interests or personal relationships that could have appeared to influence the work reported in this paper.

