# Supplementary material for: Discerning Seizure-Onset v. Propagation Zone: Pre-and-Post-Operative Resting-State fMRI Directionality and Boerwinkle Neuroplasticity Index
Source: Neuroimage Clin. 2022 May 28;35:103063. doi: 10.1016/j.nicl.2022.103063 (PMC9163994; doi:10.1016/j.nicl.2022.103063)
Supplement: Supplementary data 1 [file mmc1.docx]

**Supplementary Table 1. Patient ROIs, Pre and Post-Operative Imaging, and Directionality Result Matrices**

| # | A. Directionality HH SOZ: sagittal, axial, and coronal T1W with HH SOZ overlayed in RED | B. Directionality Propagation ROI: sagittal, axial, and coronal T1W with propagation ROI overlayed in RED | C. Pre-operative Searchlight used to select the HH SOZ and Propagation ROI for Directionality: top row sagittal, coronal, and axial T1W with HH SOZ voxel from SL selected to be the HH SOZ for directionality based on bottom row of images with show the region of connectivity in the rest of the brain to which the voxel had greatest connectivity. Note aliased noise outside of brain occurs. Color bar of connectivity values, yellow- red positive/relative activation, blue-green negative/relative deactivation  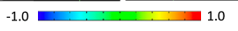 | D. Post-operative Images Used to verify Ablation of the HH SOZ for  Directionality: Diffusion weighted, and post-contrast images taken immediately  after ablation. Patient 5-28 with same pre-op SL results overlayed directly on the  post-op imaging for direct verification. | E. Directionality Result Matrix. How to read the matrix: example patient 4 – From HH to HH is light blue, therefore mild inhibitory connection. pZ to HH is dark blue thus strong inhibition. HH to pZ is yellow-orange thus excitatory. pZ to pZ is light blue thus mild inhibition. |
| --- | --- | --- | --- | --- | --- |
| P1 | 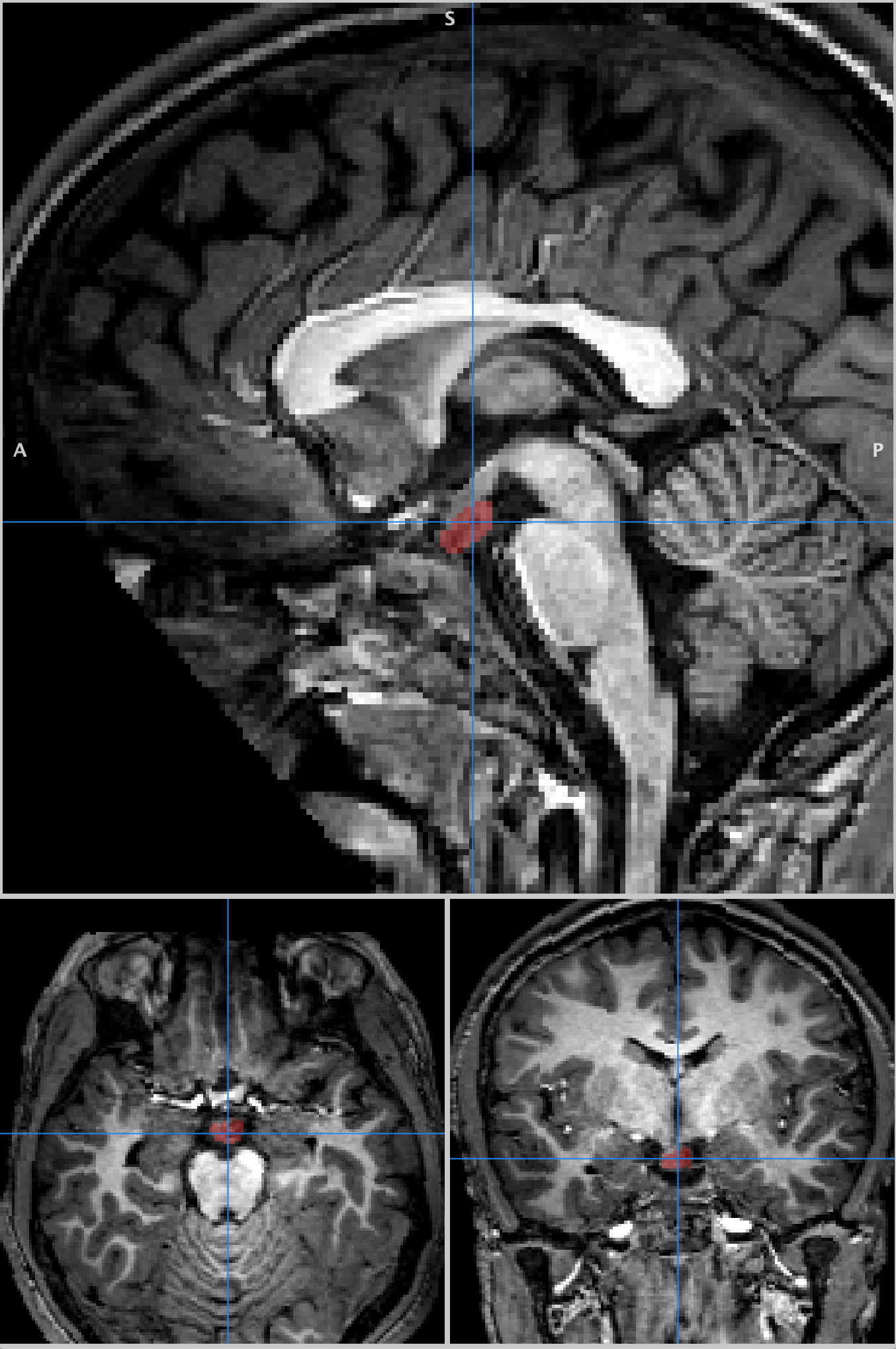 | 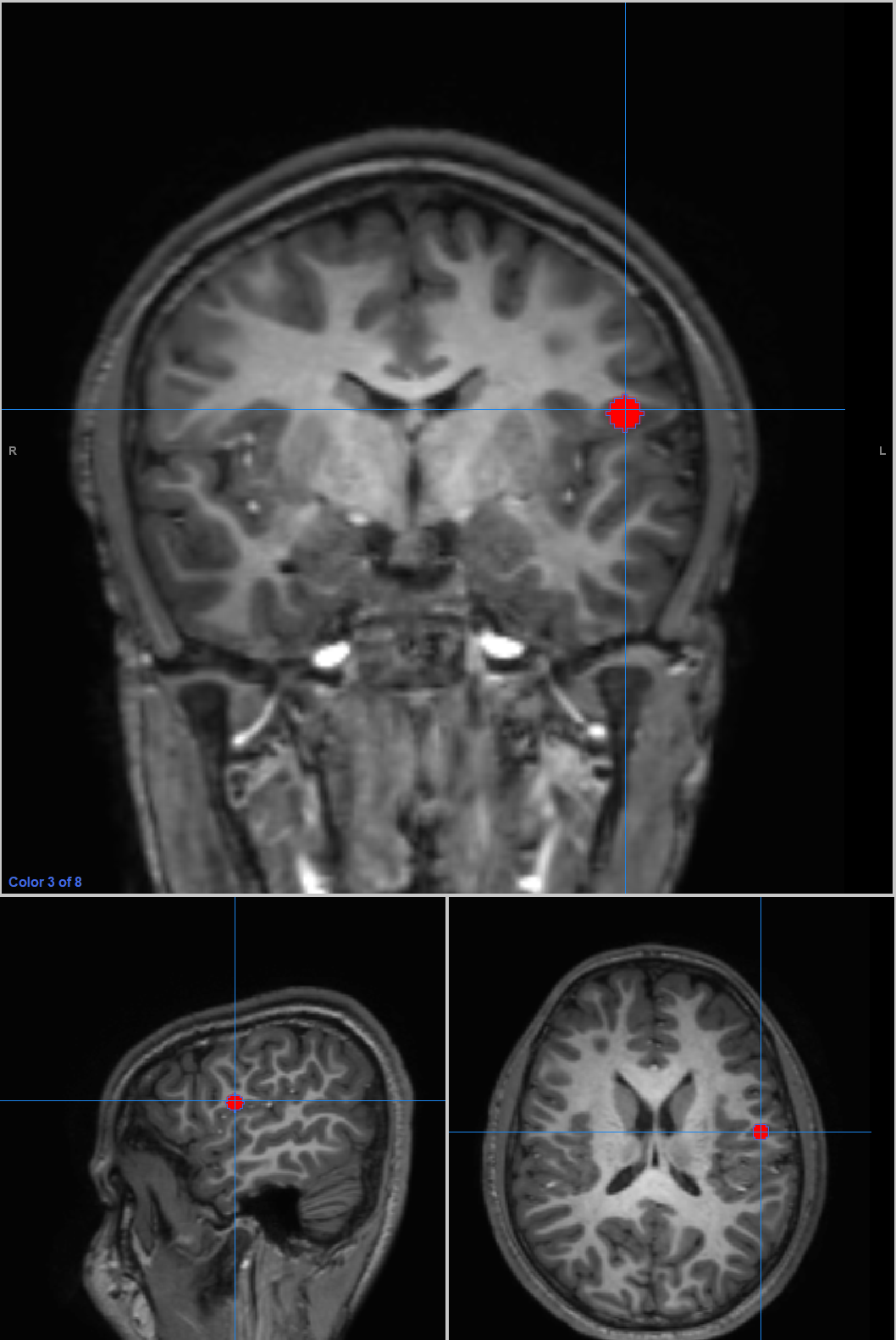 | 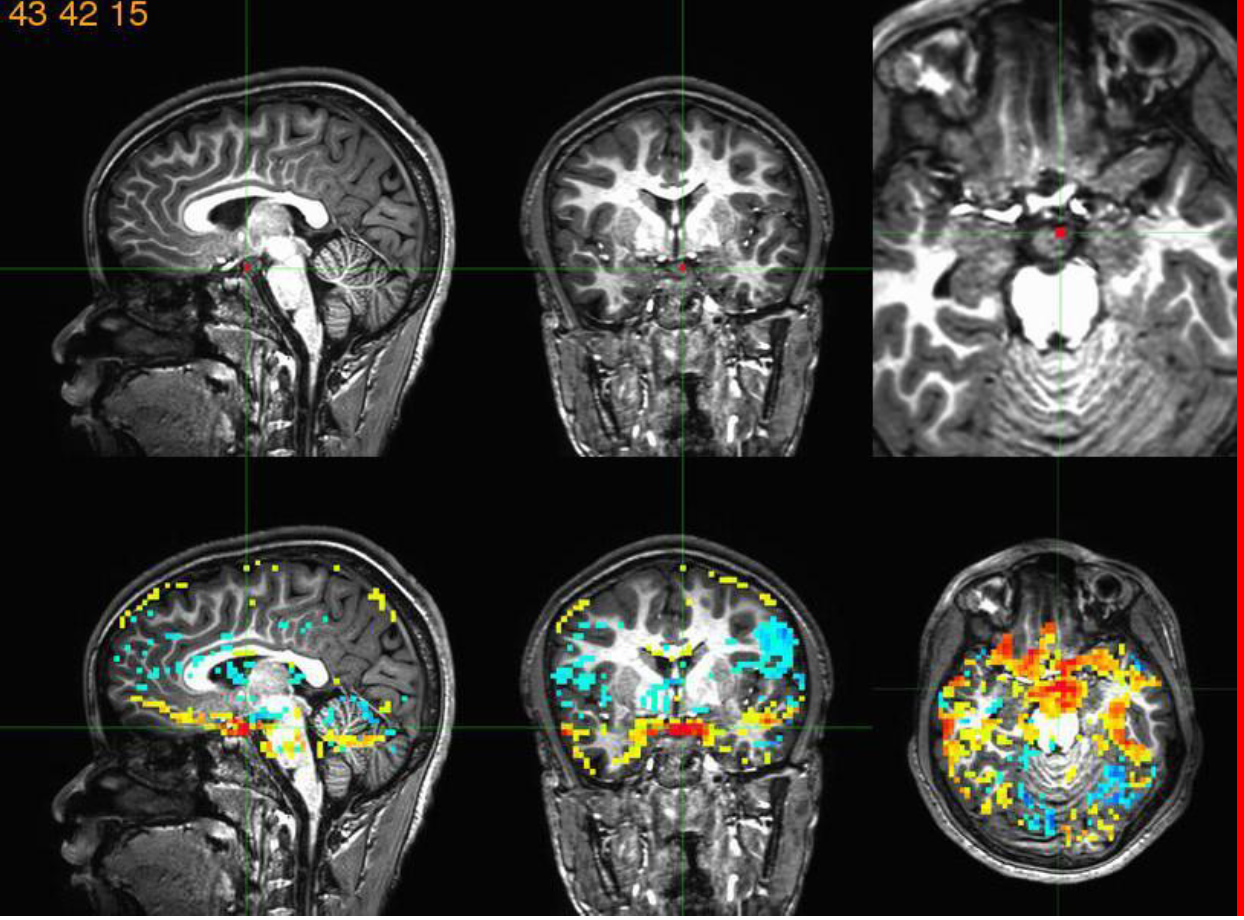 | 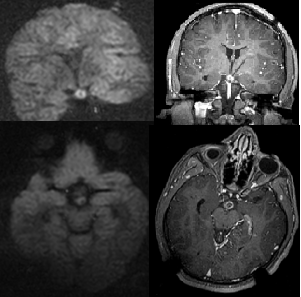 | 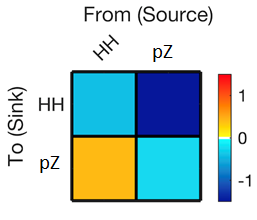 |
| P2 | 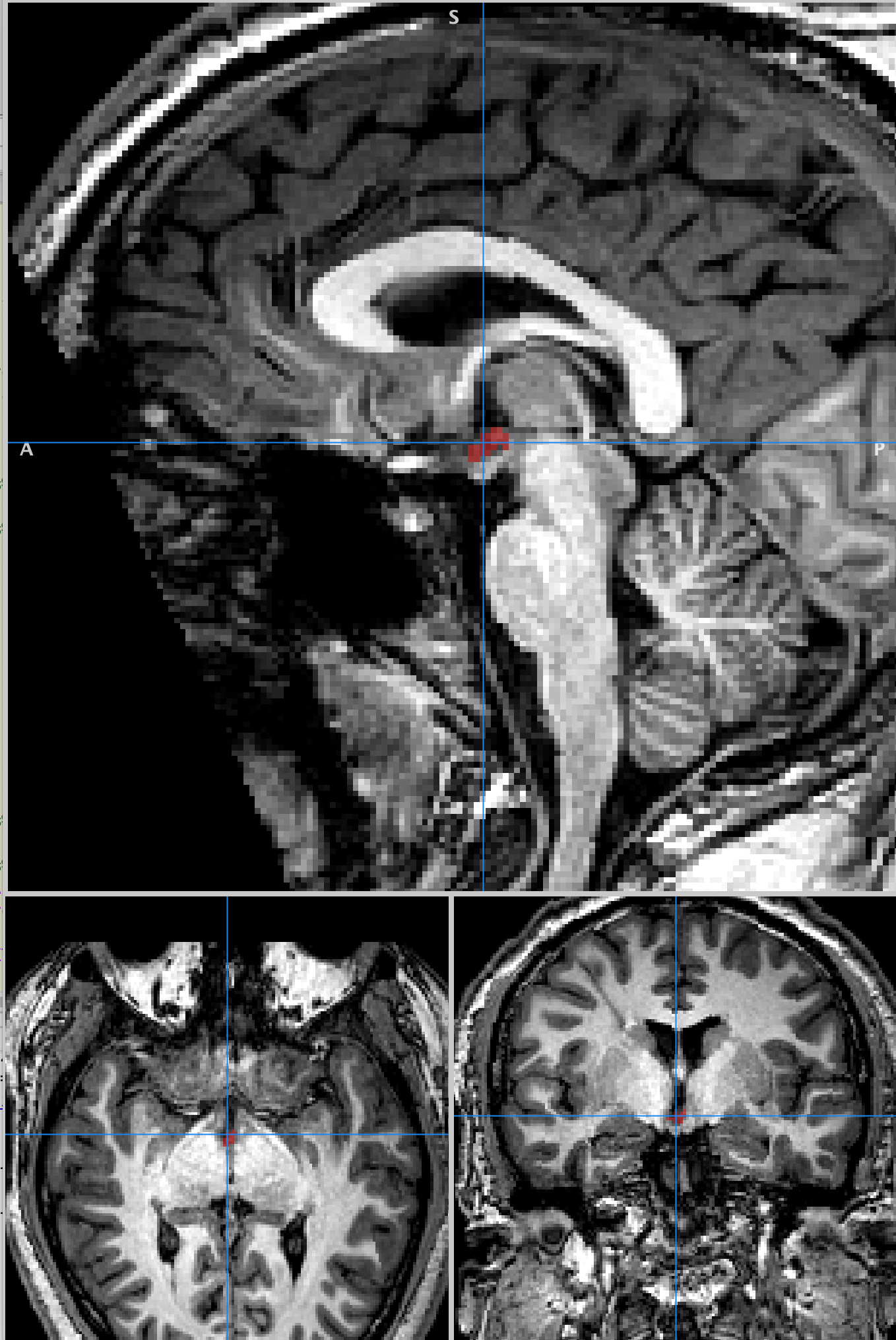 | 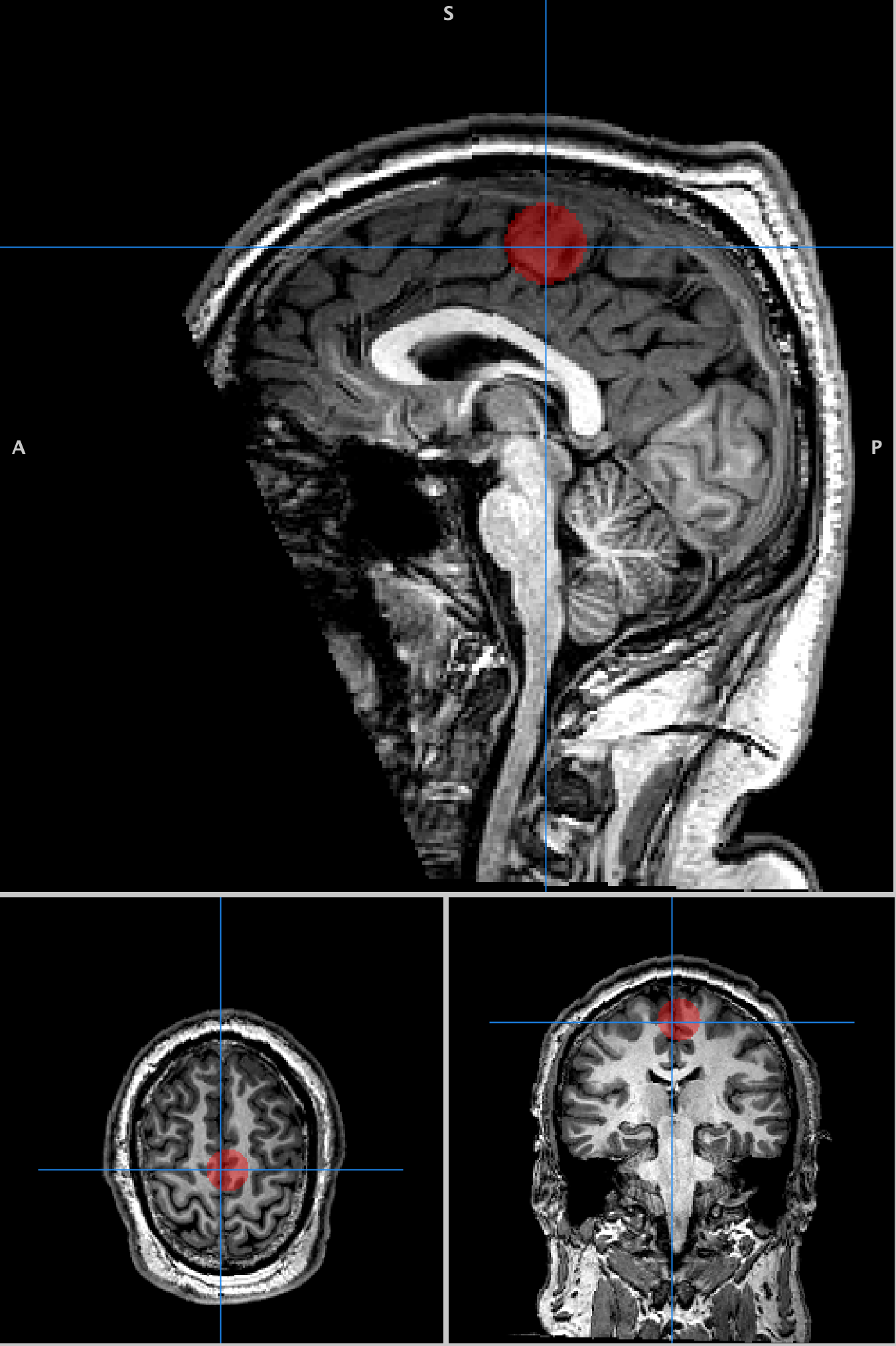 | 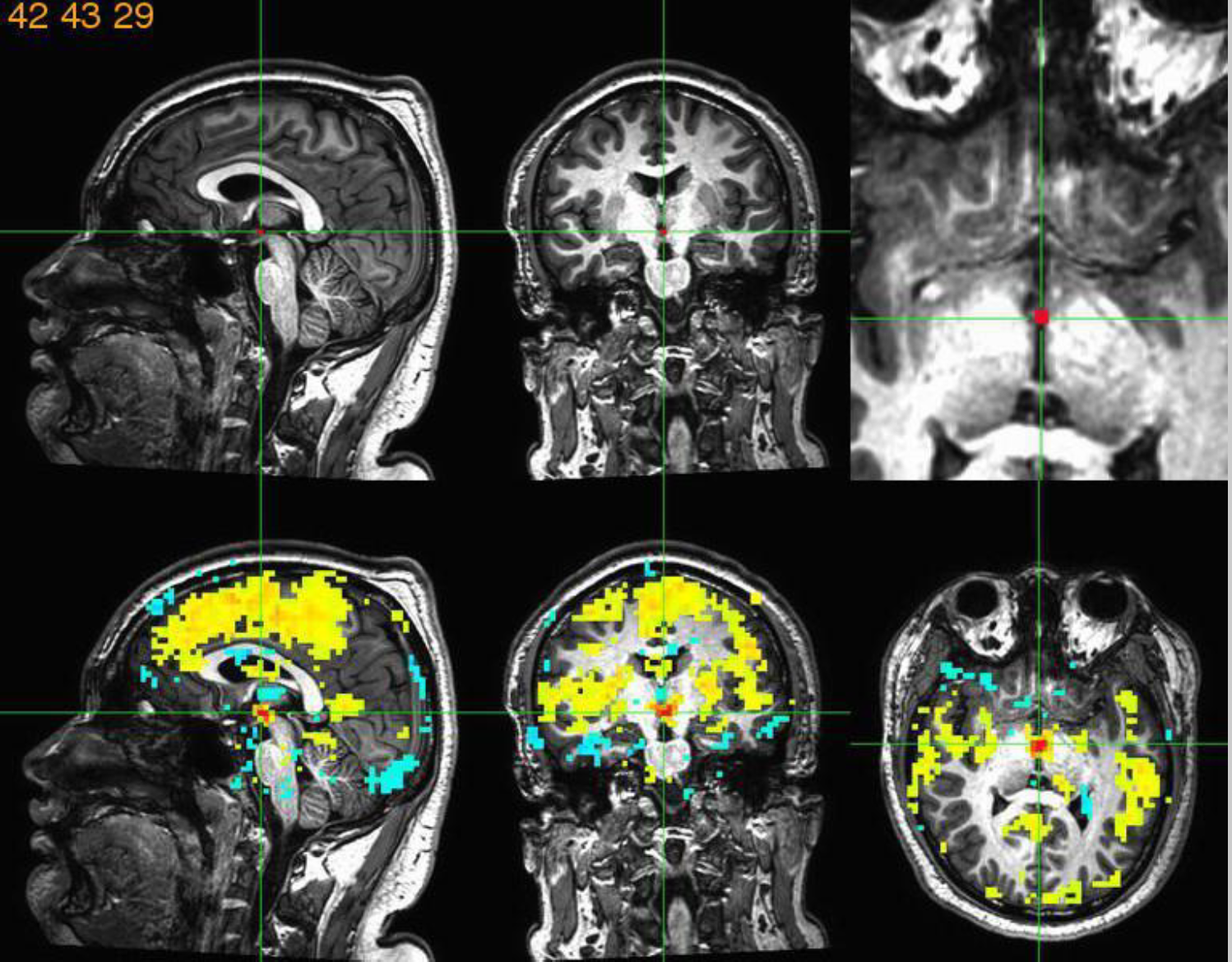 | 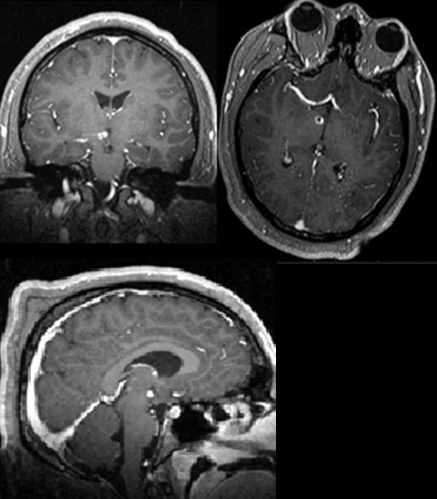 | 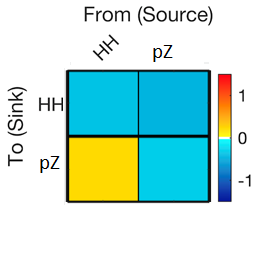 |
| P3 | 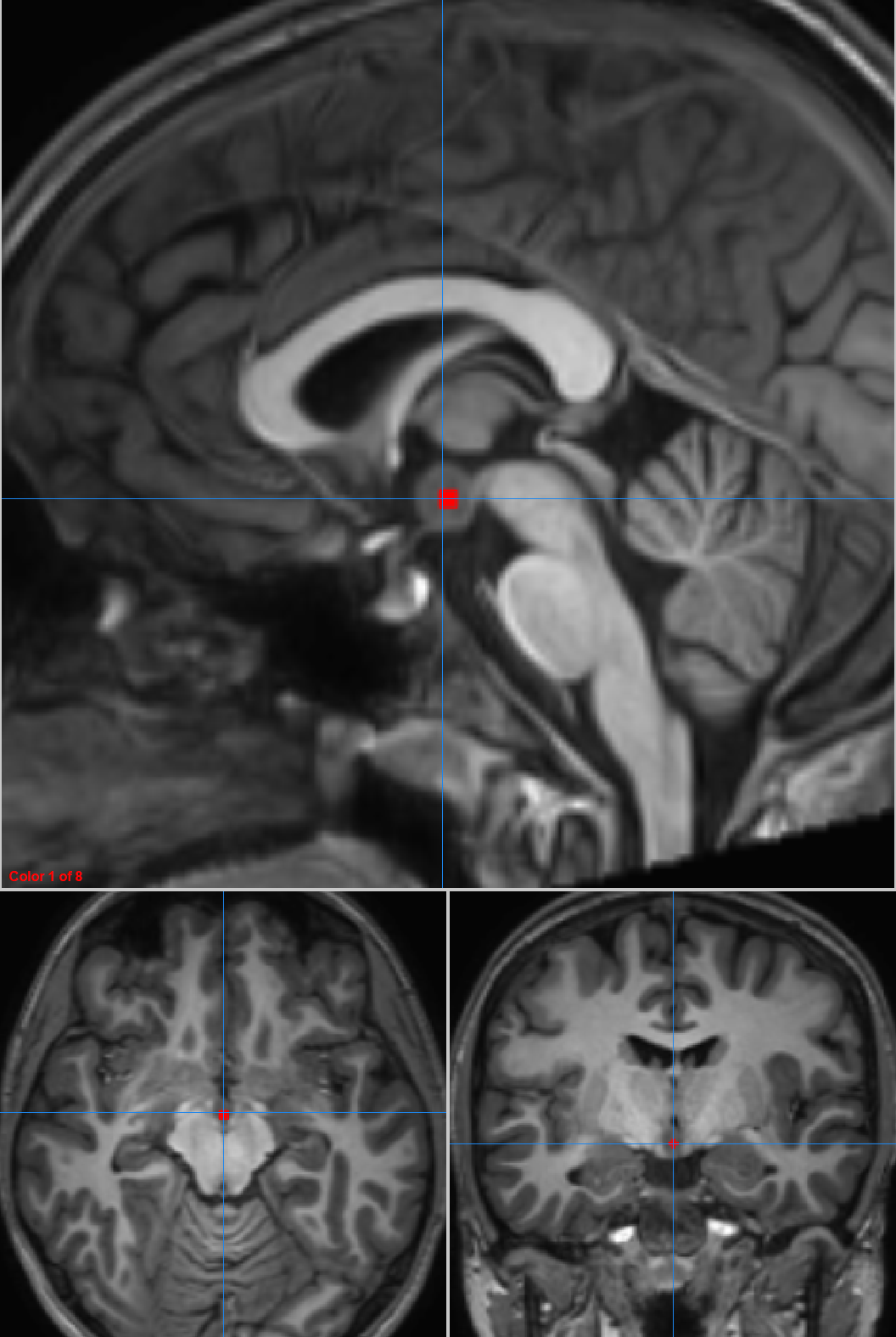 | 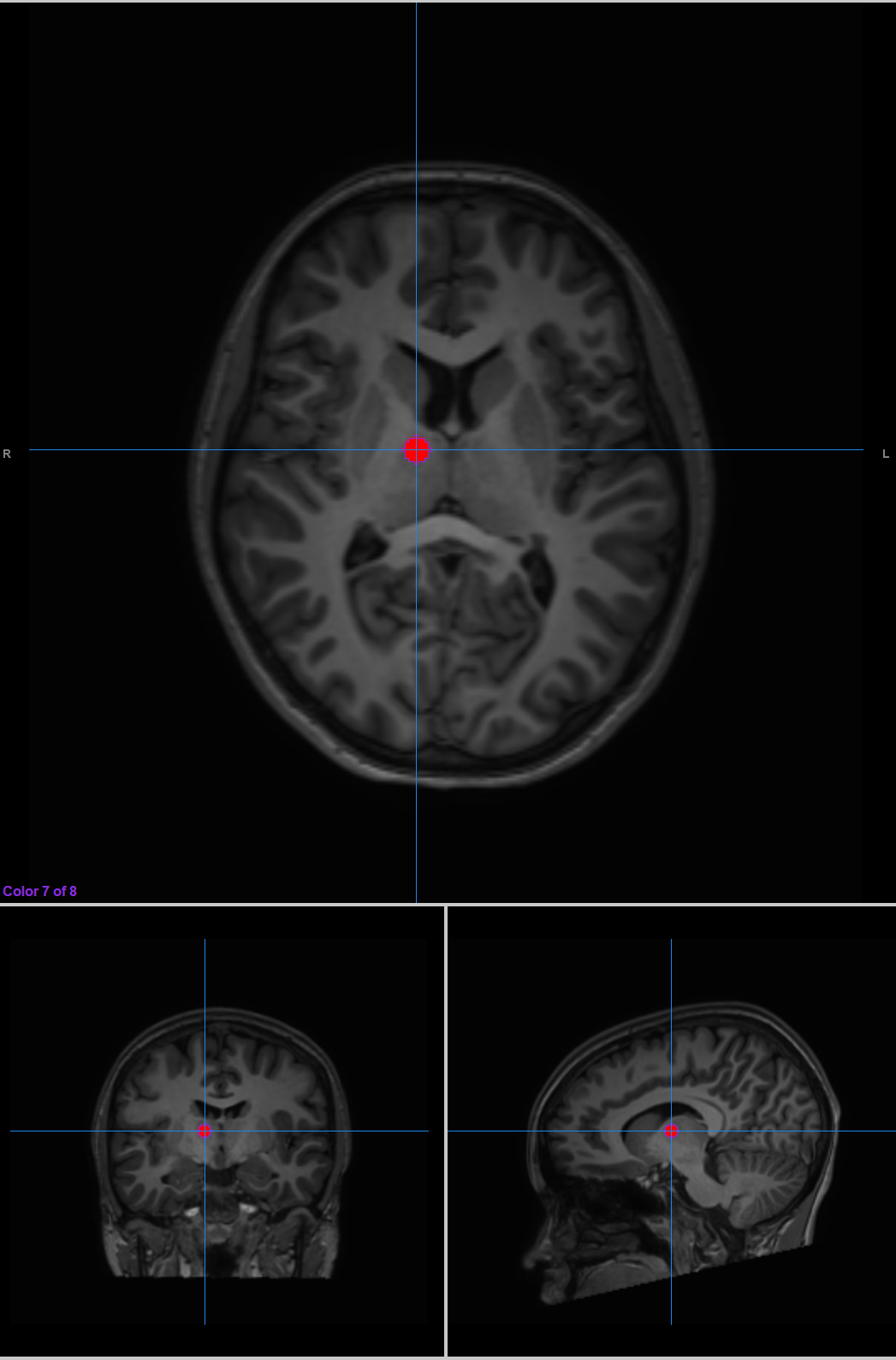 | 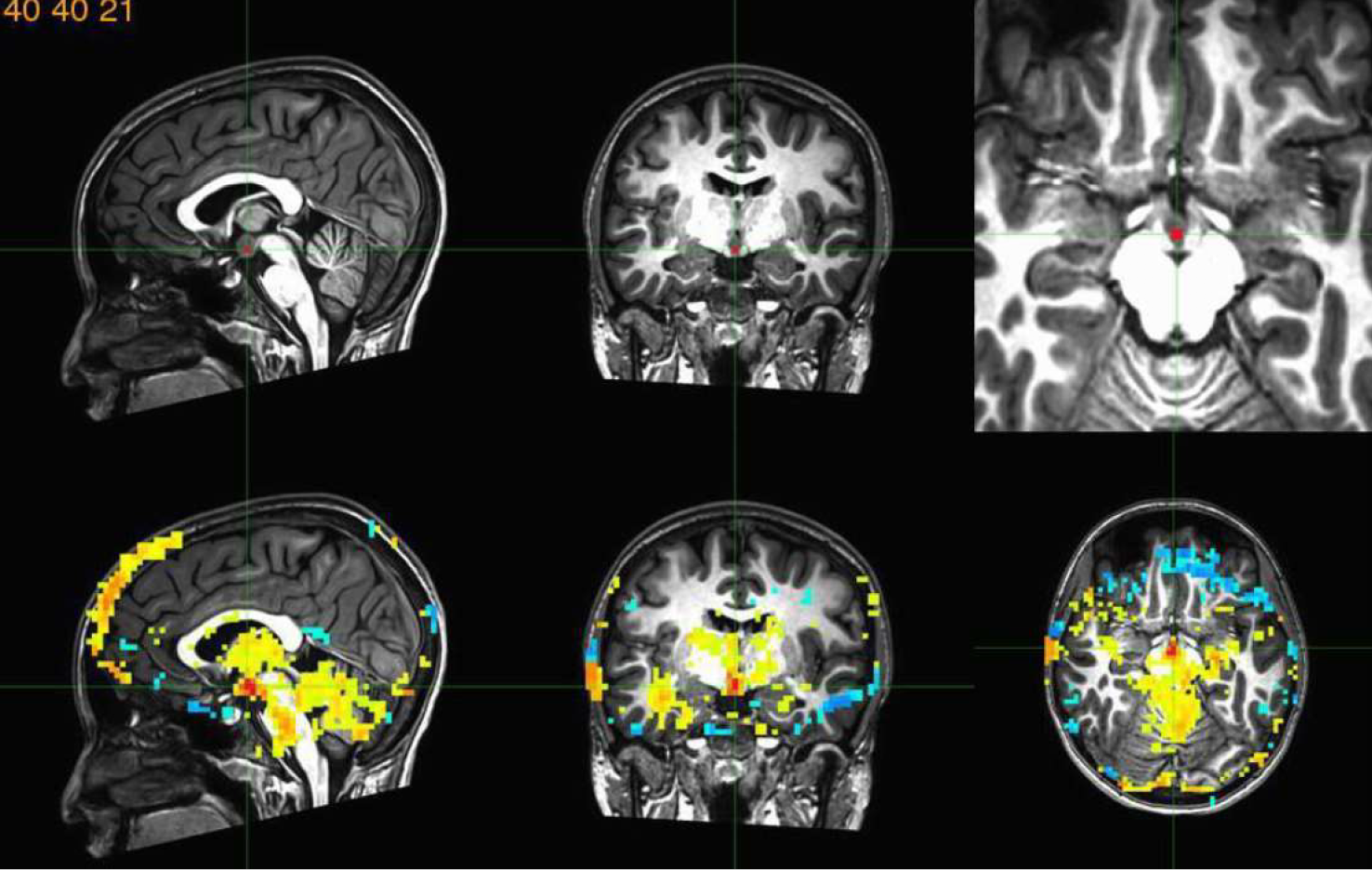 | 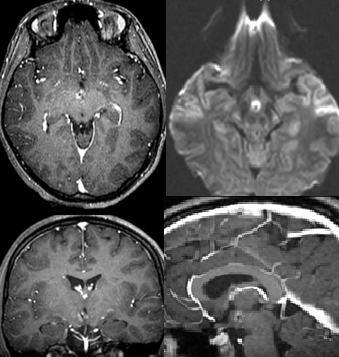 | 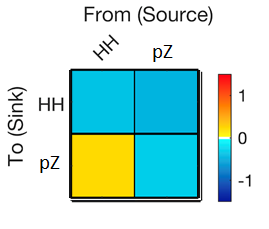 |
| P4 | 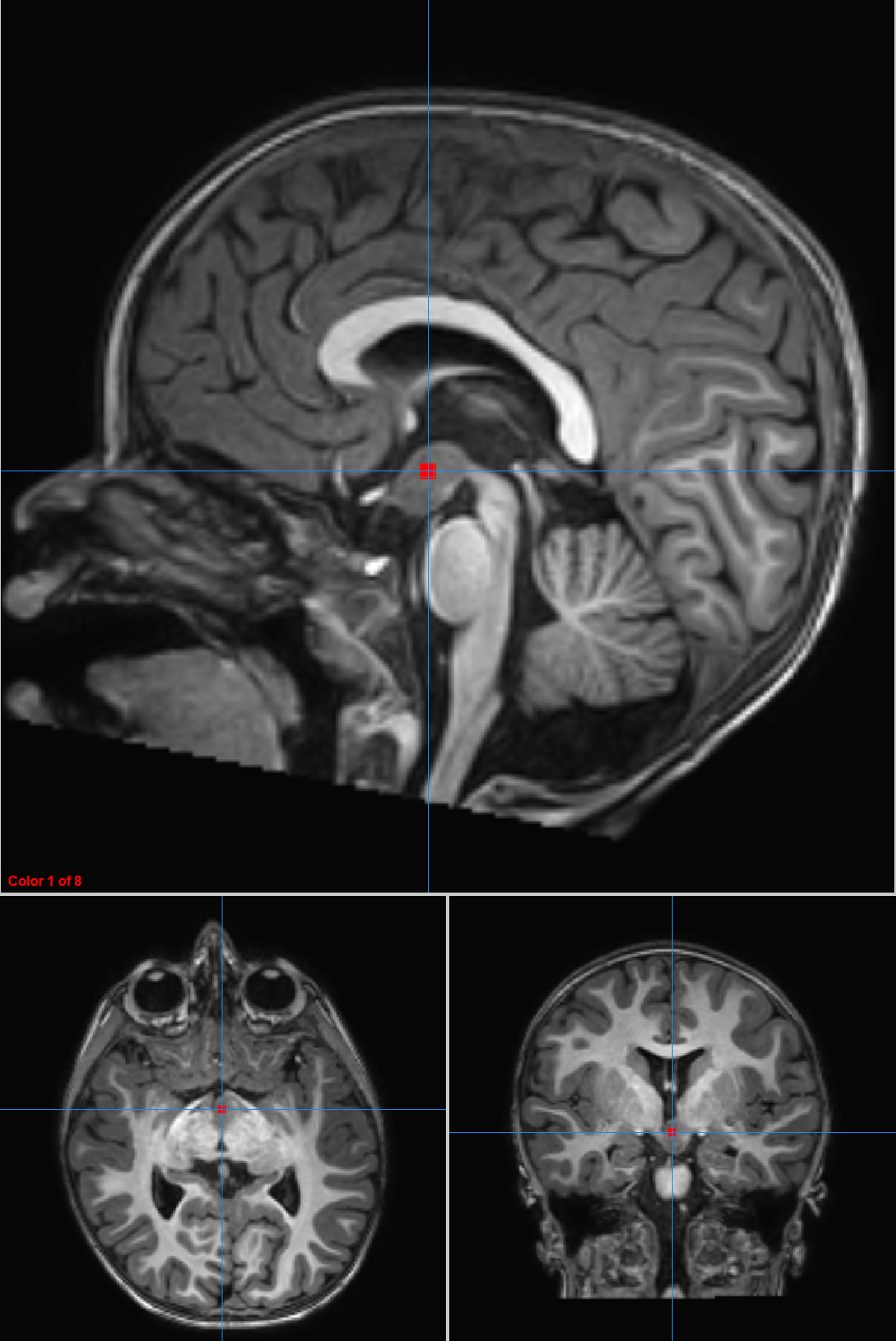 | 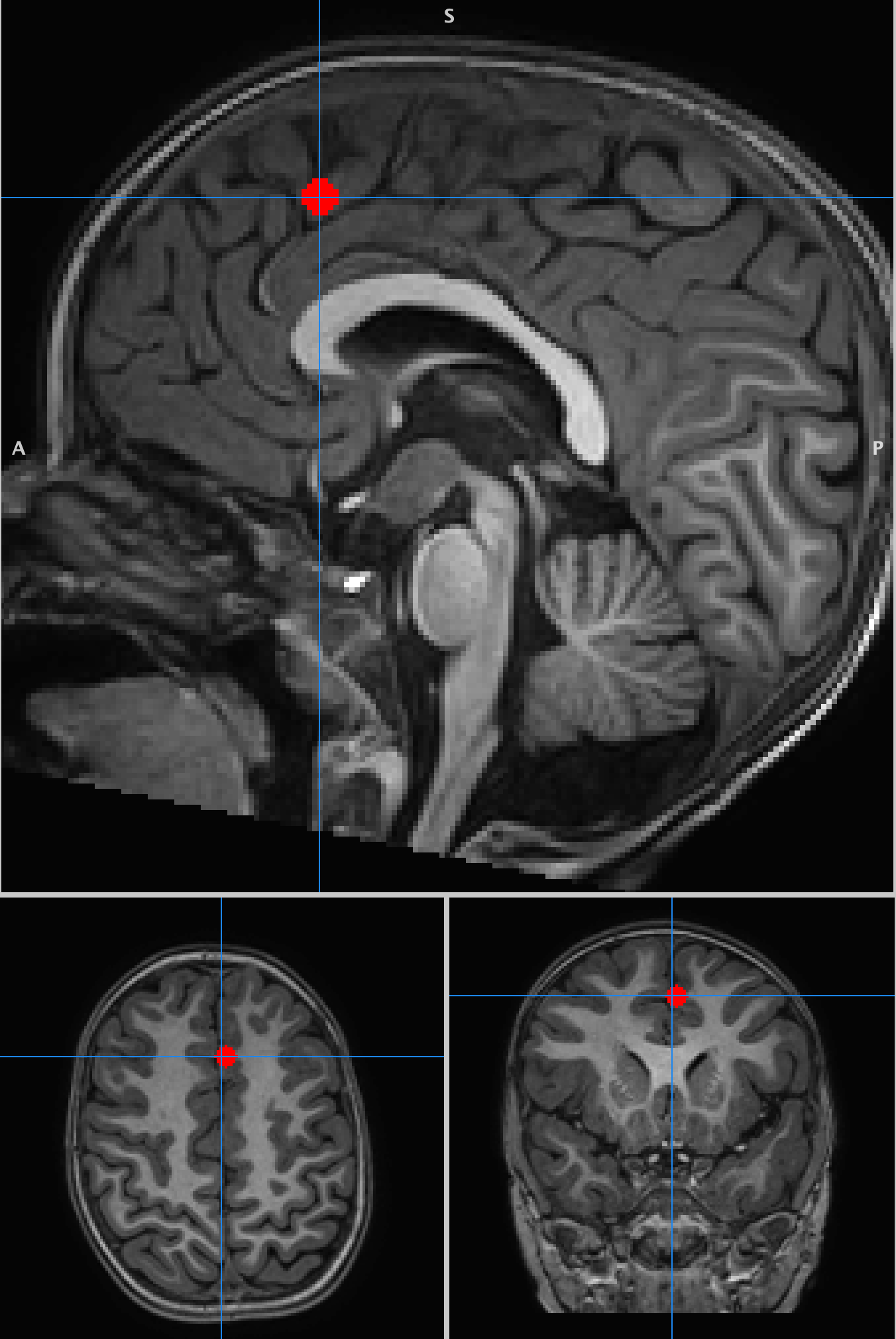 | 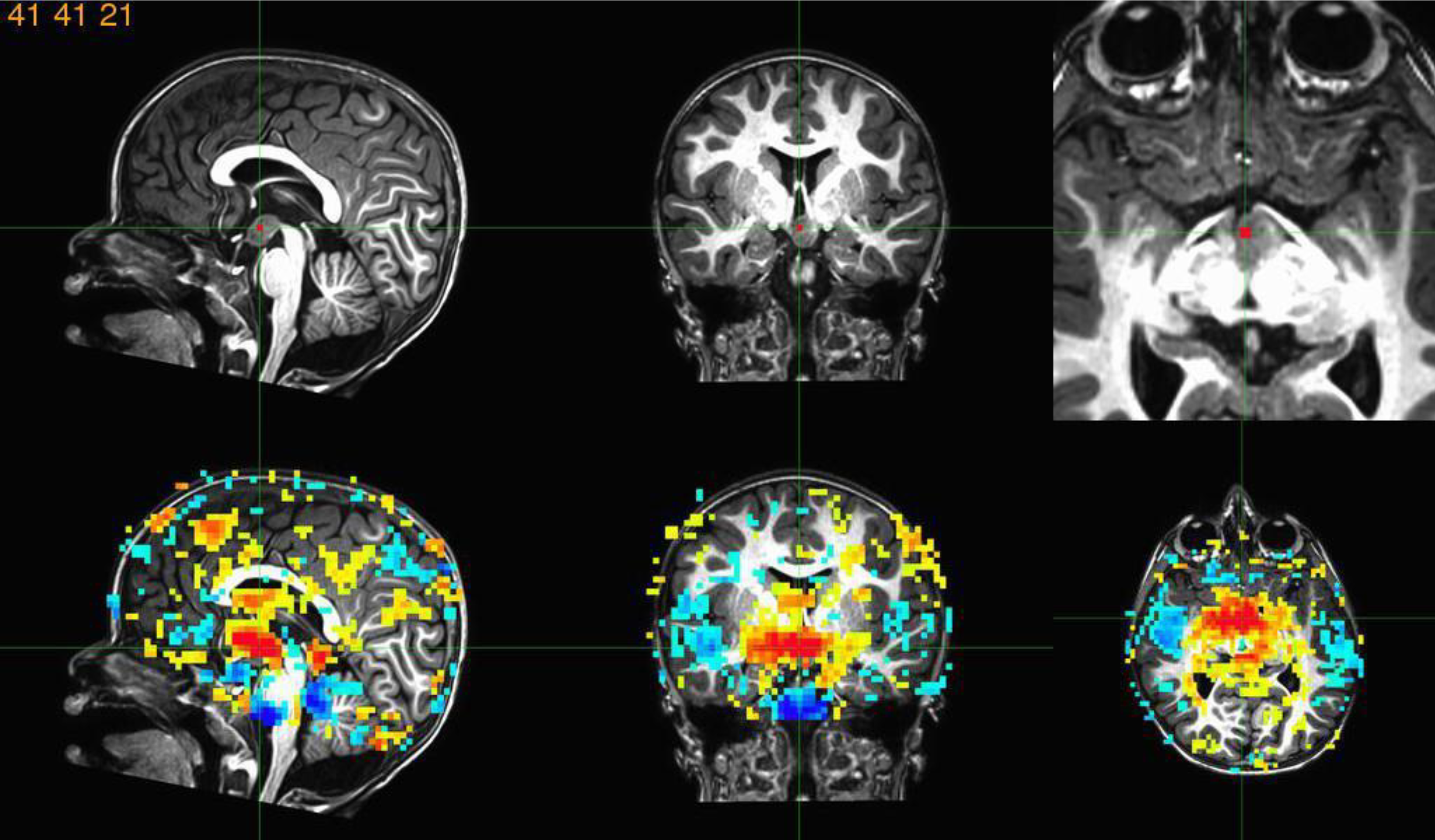 | 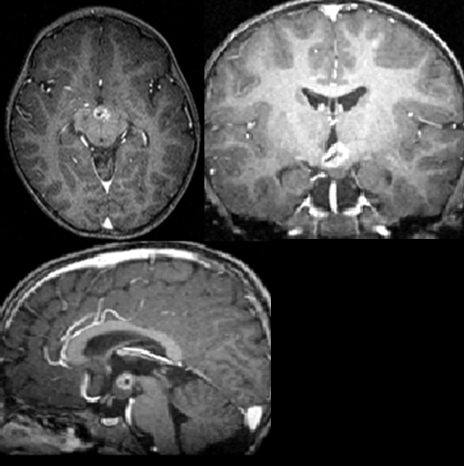 | 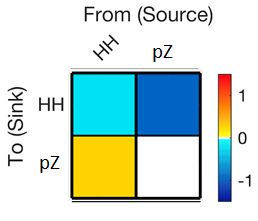 |
| P5-T1 | 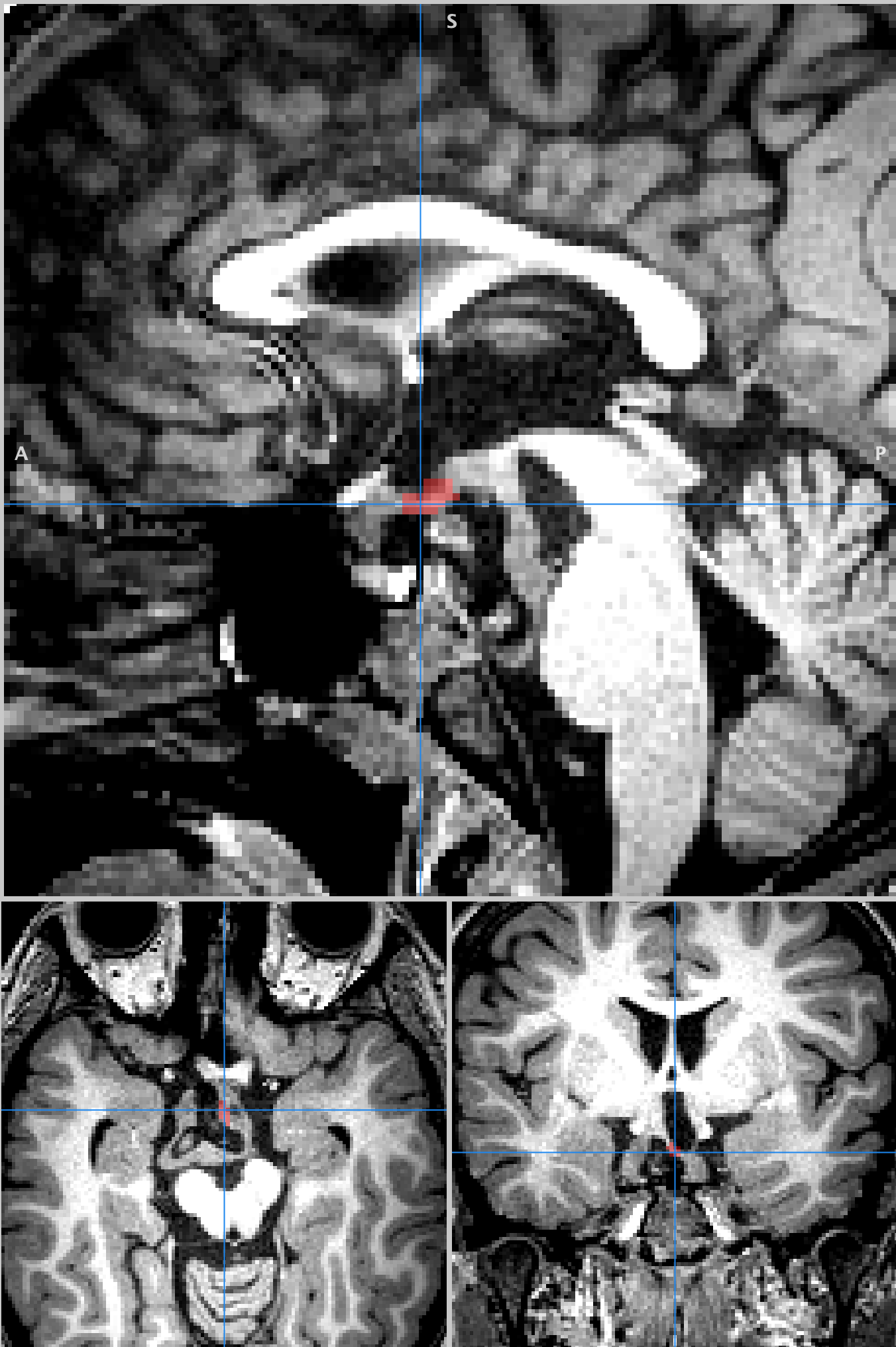 | 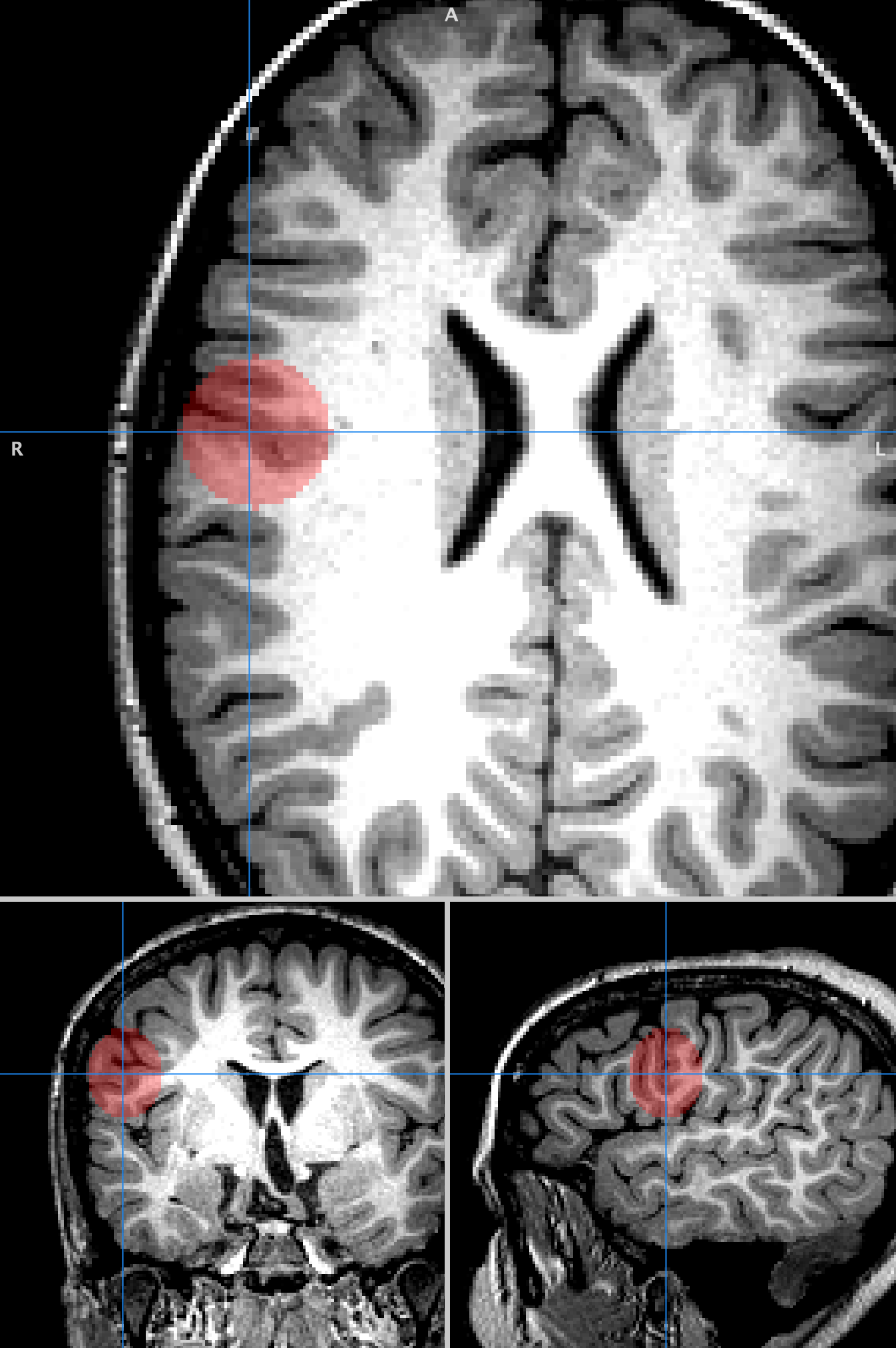 | 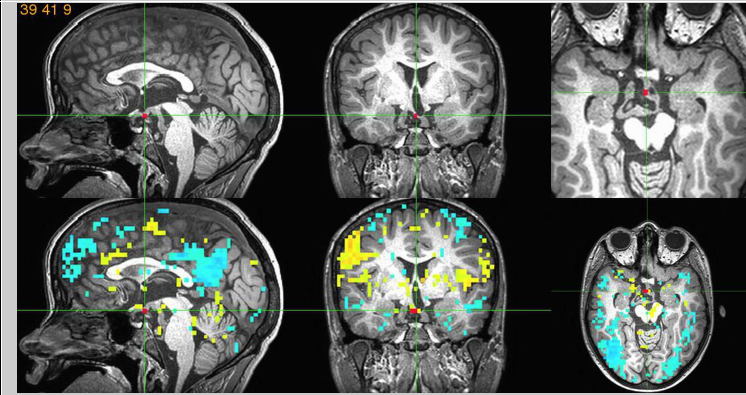 | 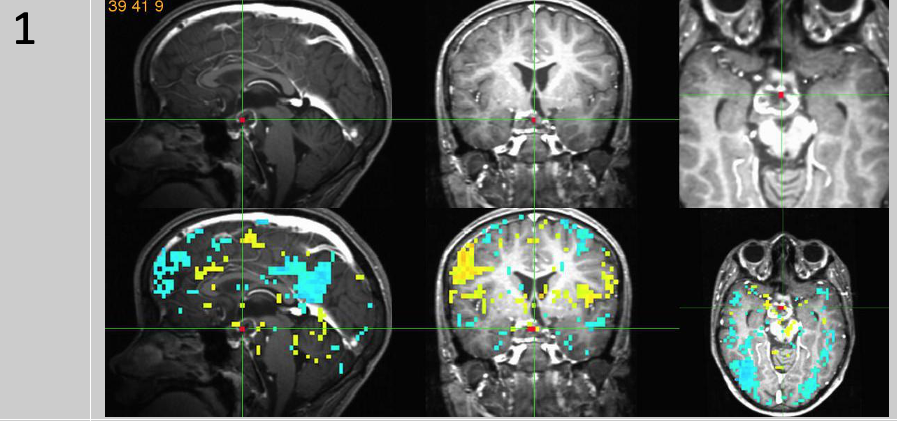 | 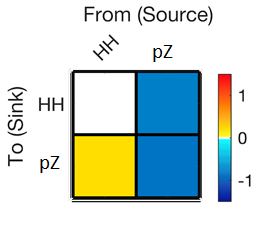 |
| P6-T2 | 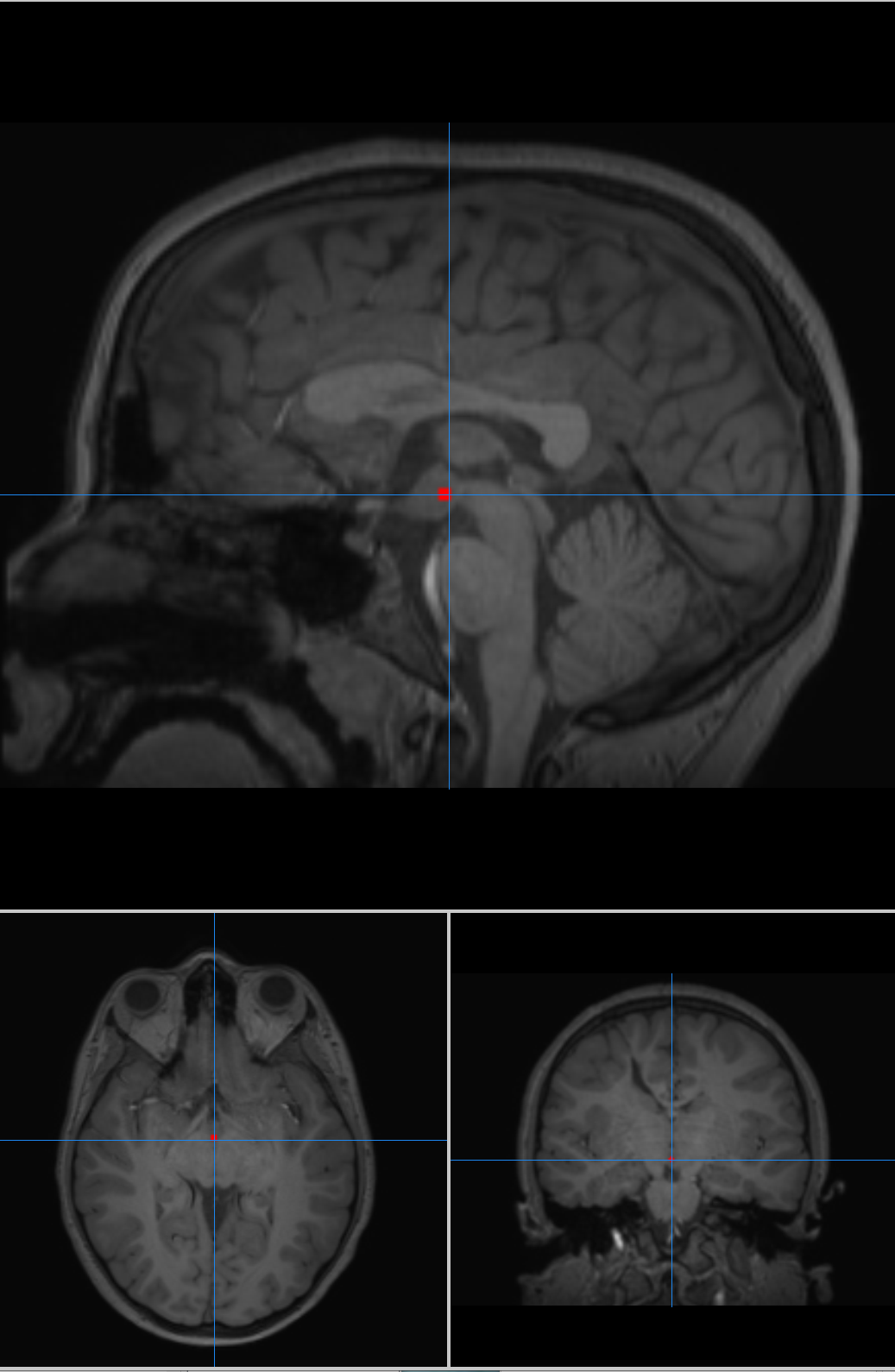 | 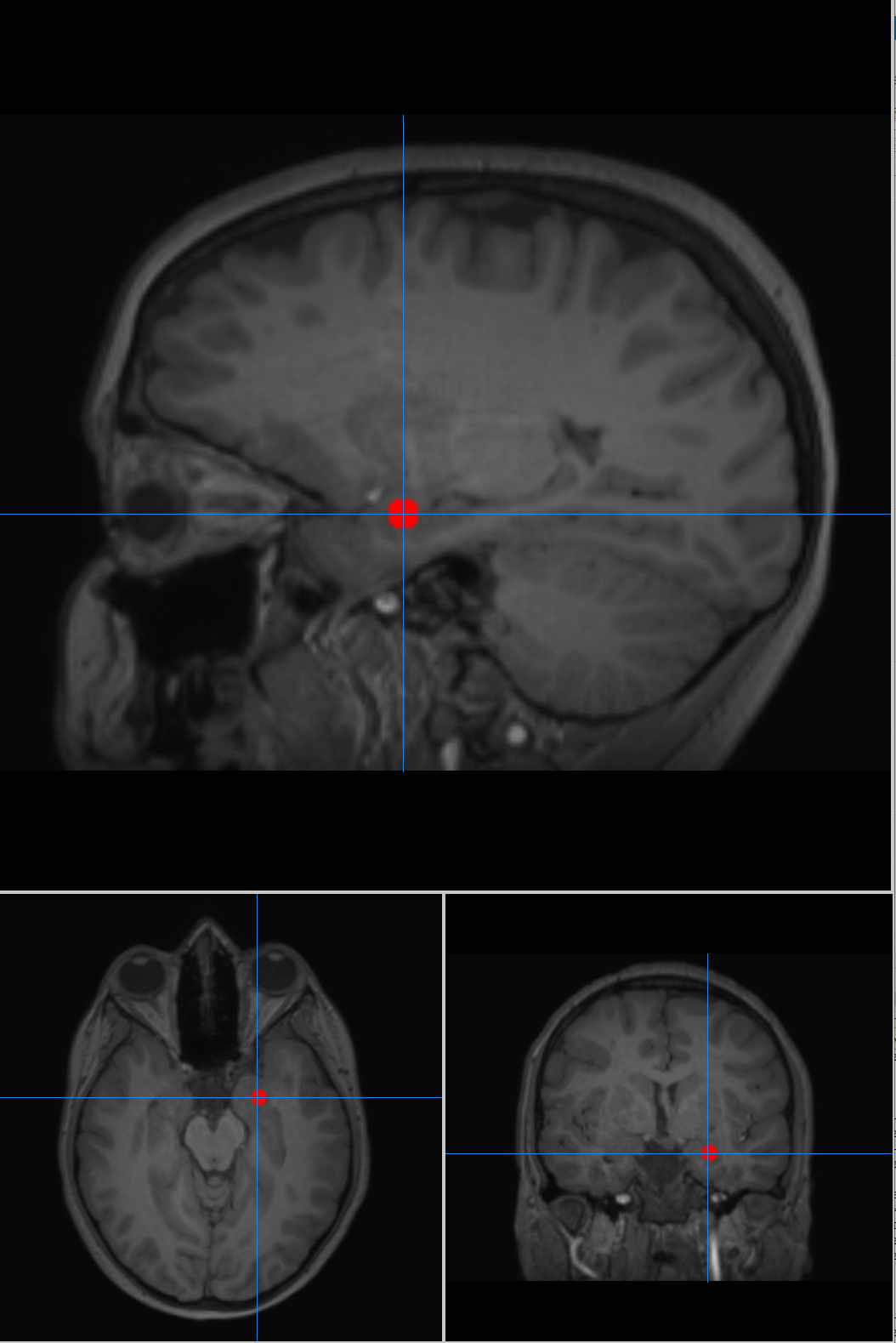 | 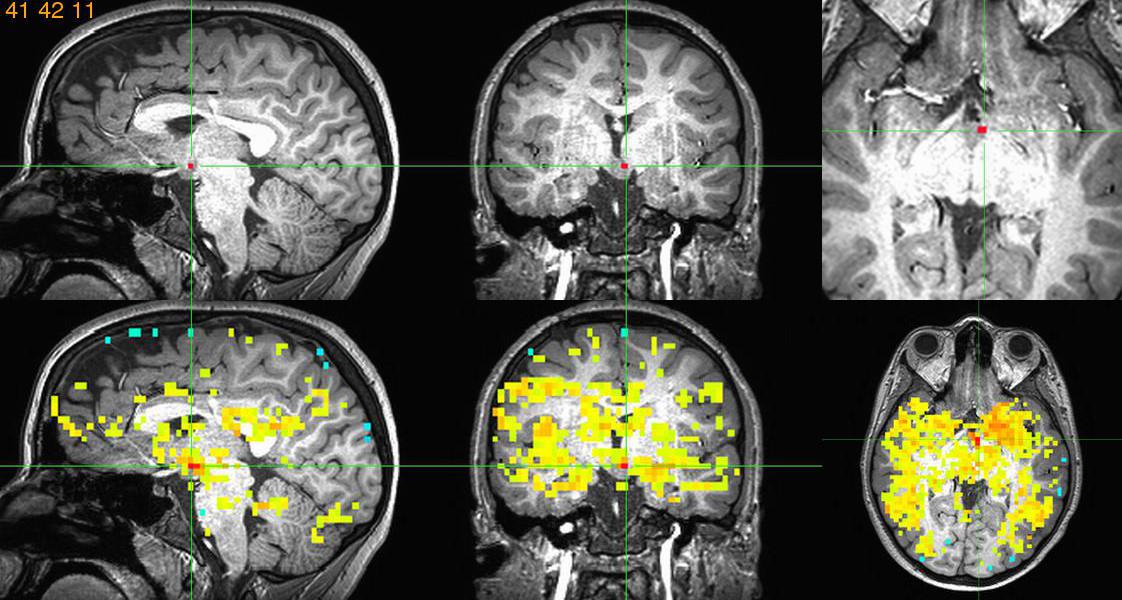 | 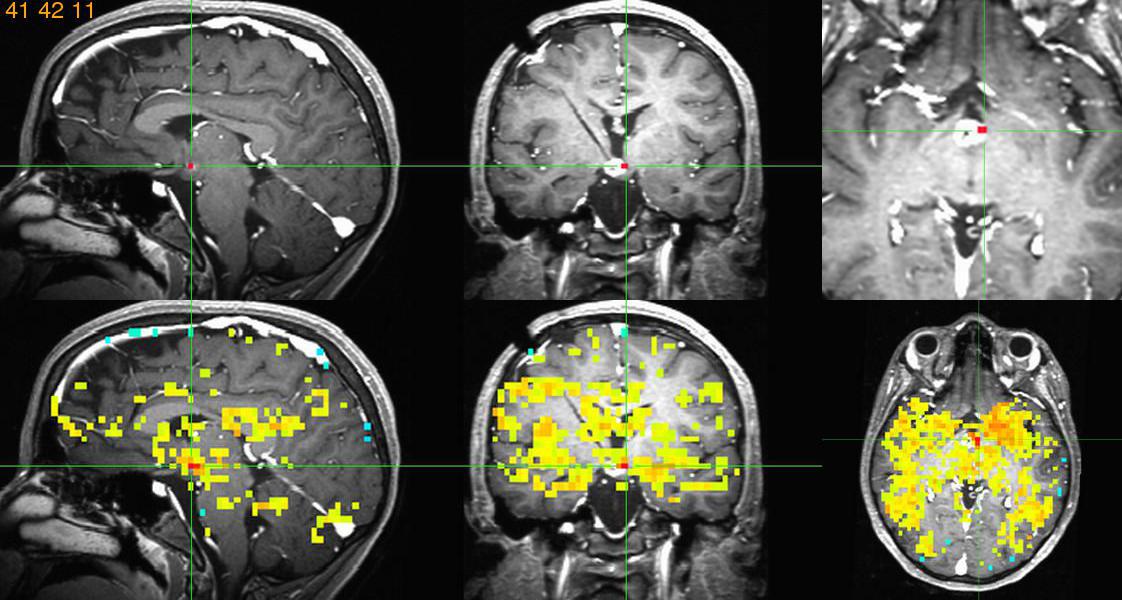 | 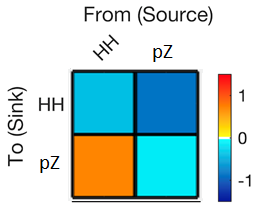 |
| P7-T3 | 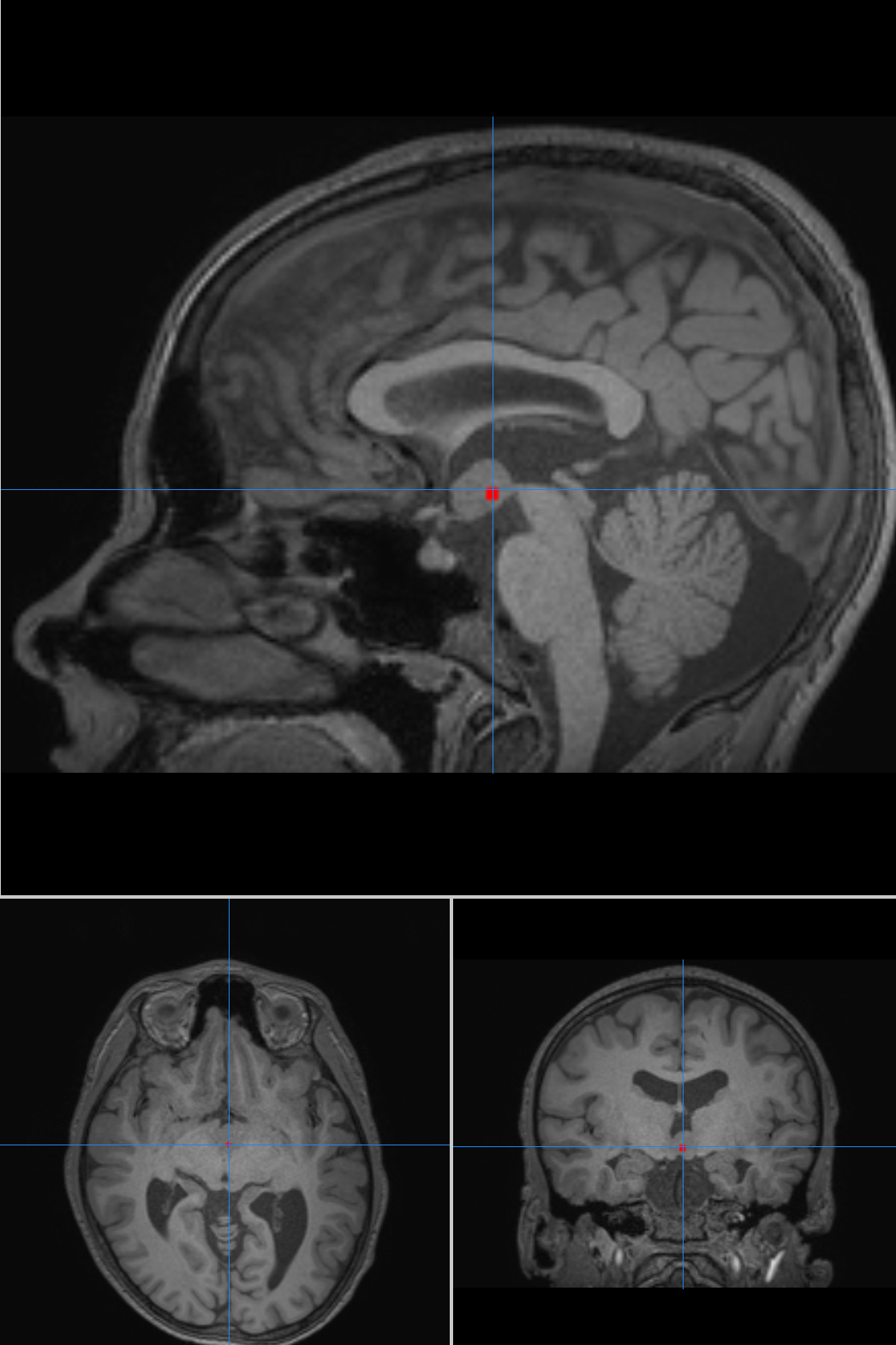 | 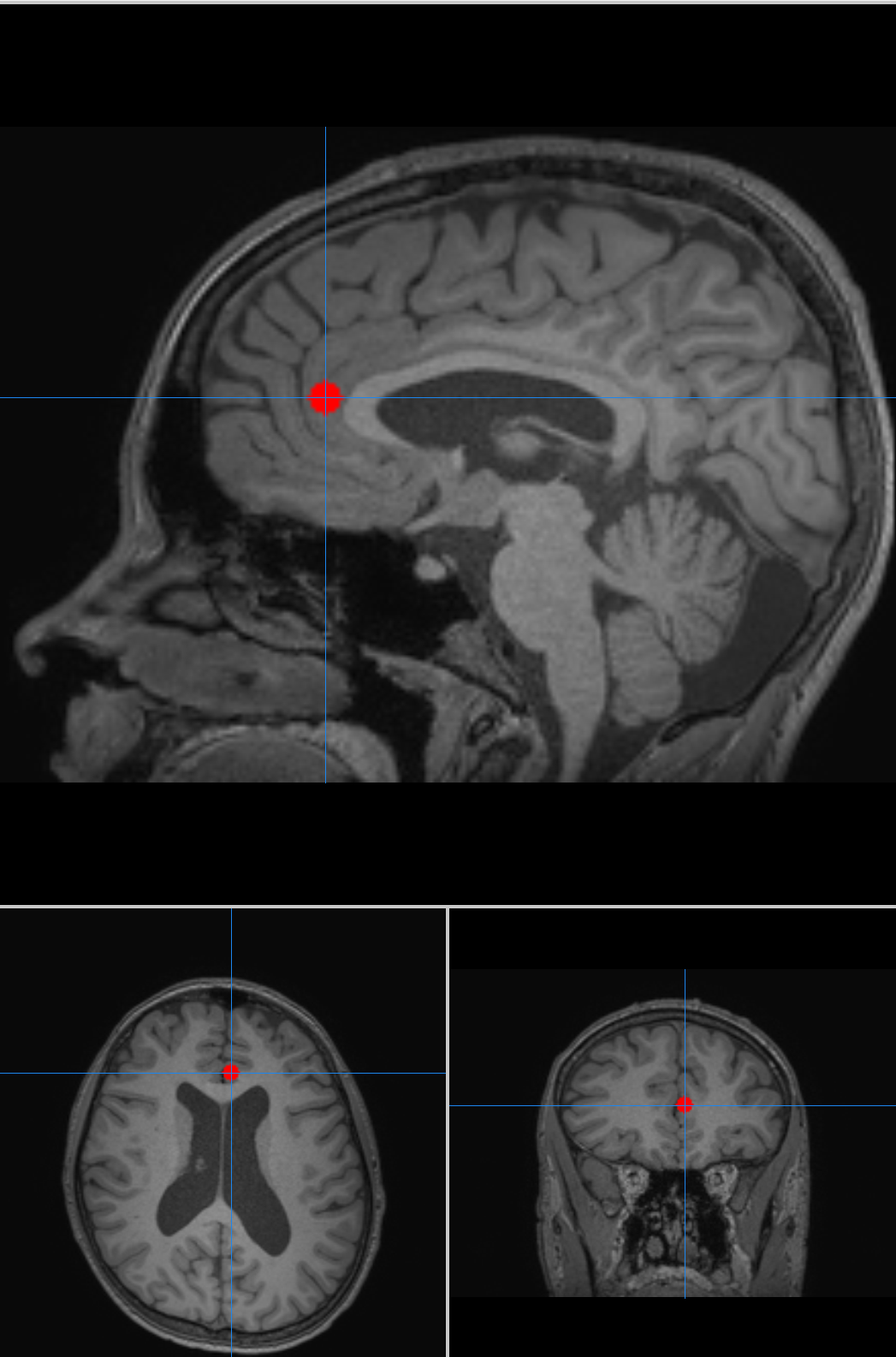 | 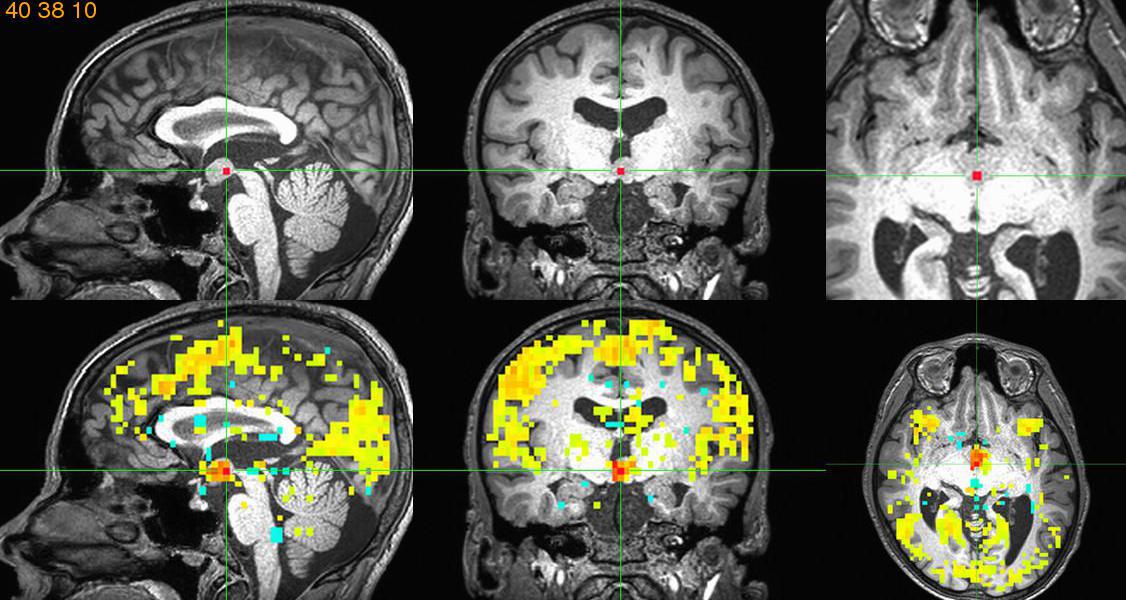 | 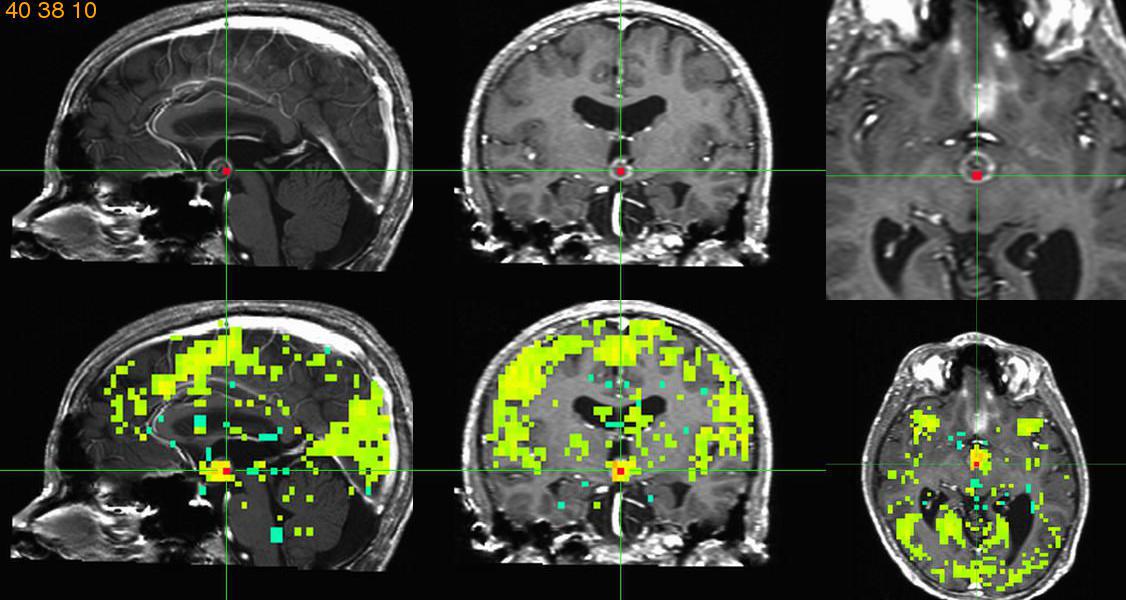  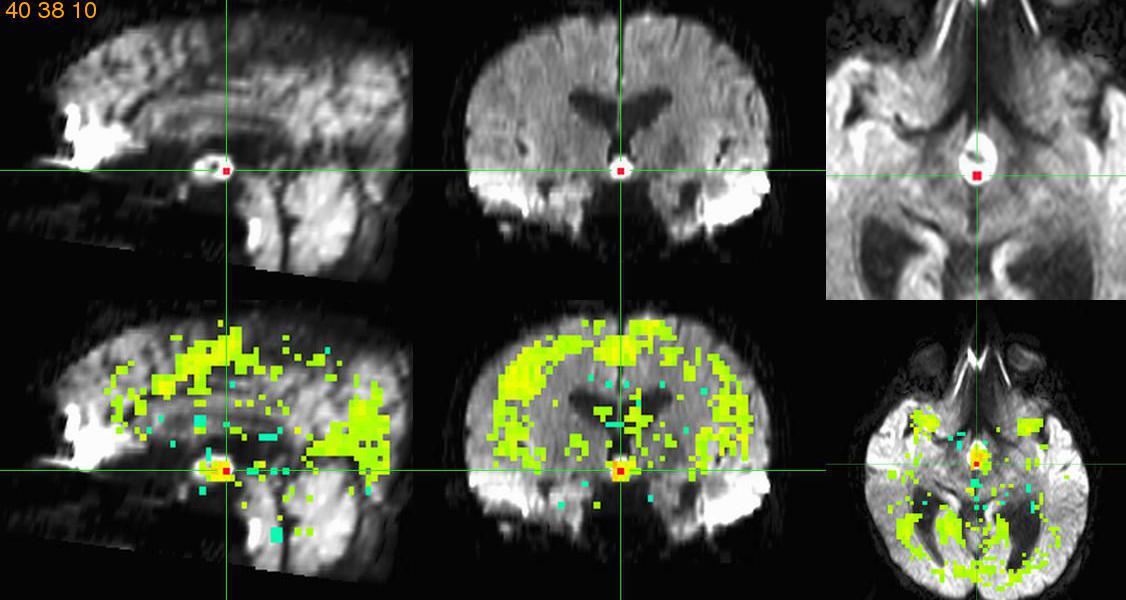 | 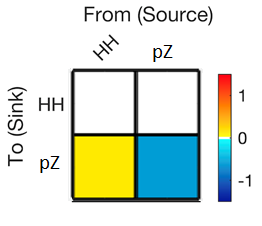 |
| P8-T4 | 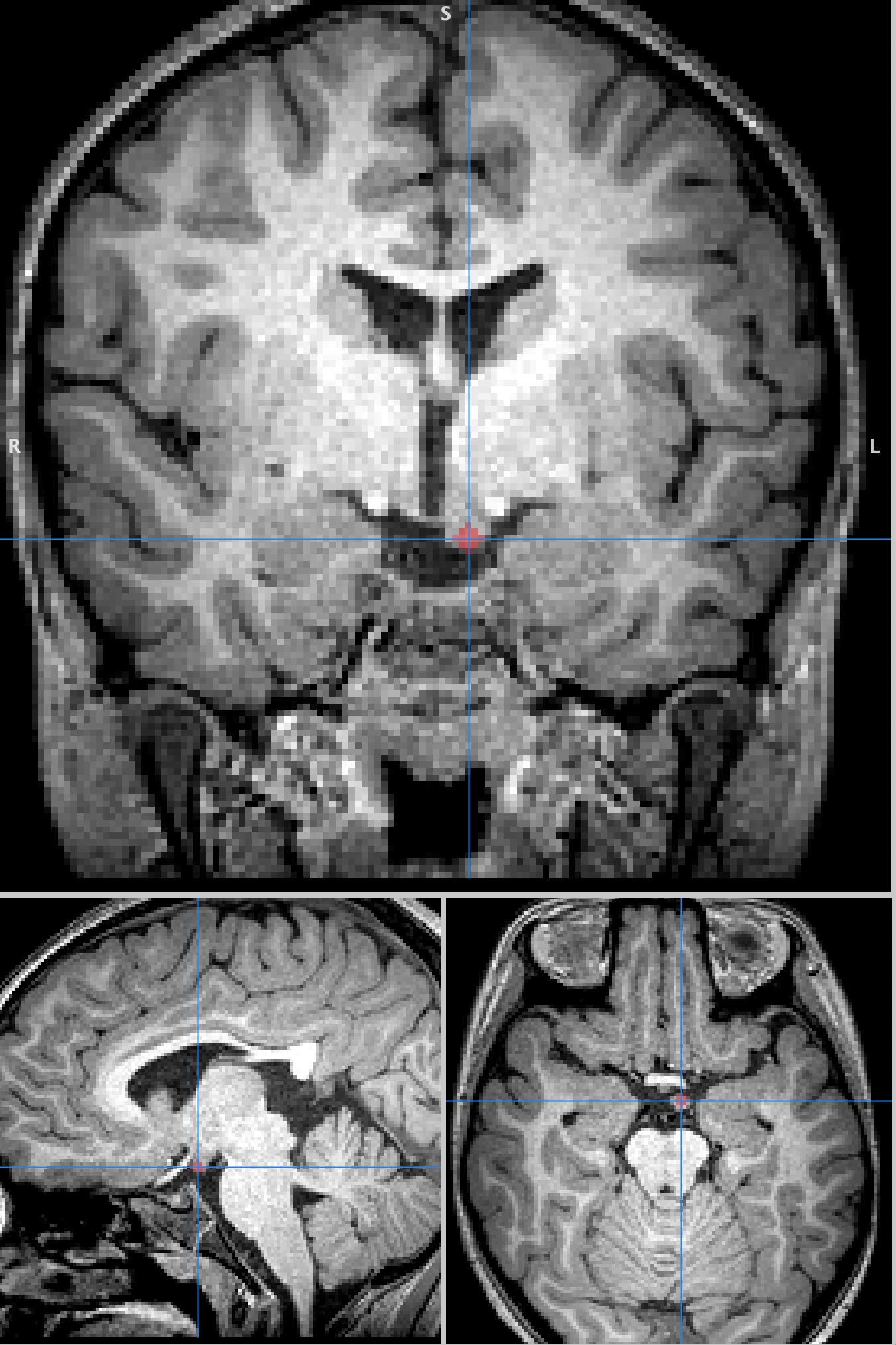 | 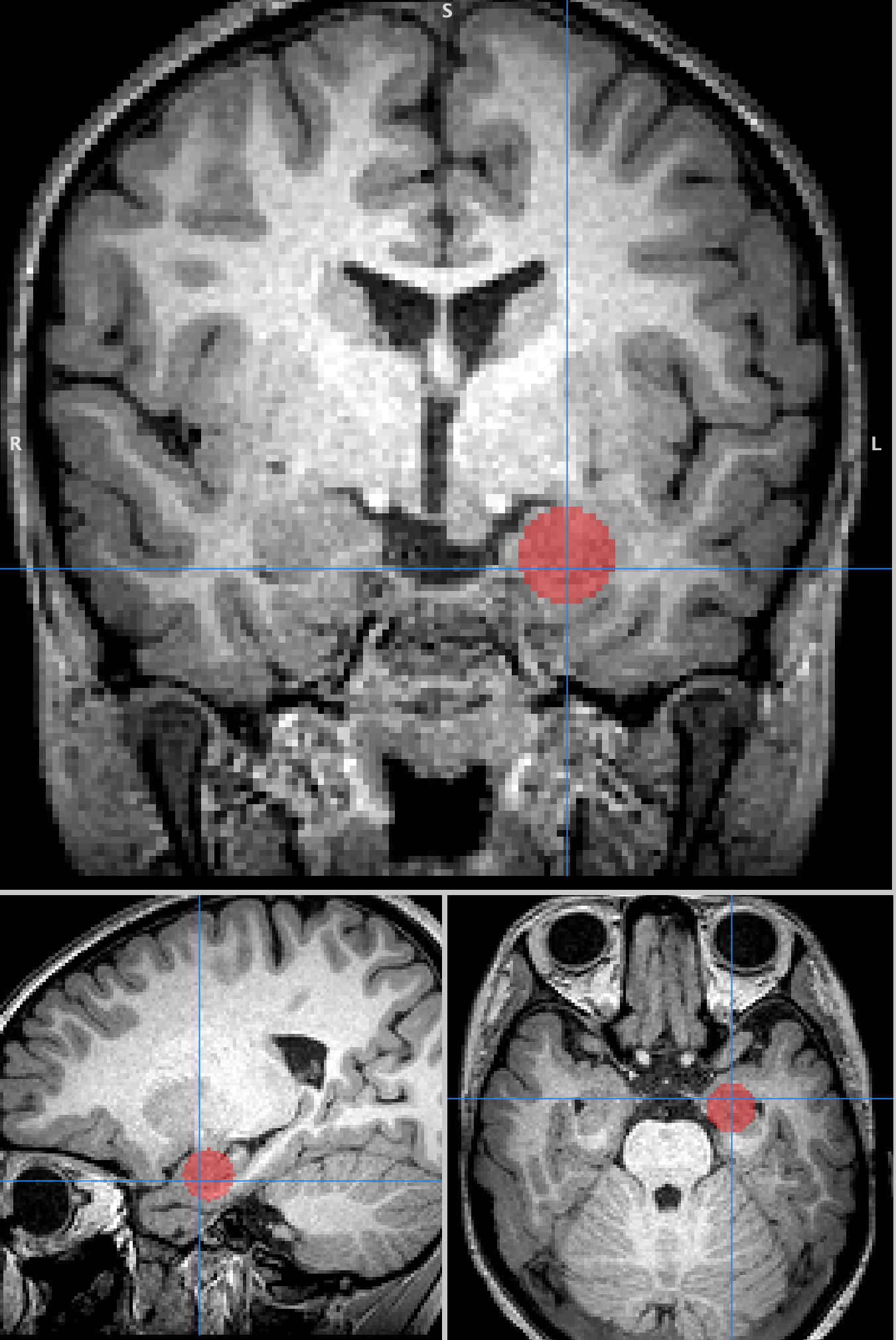 | 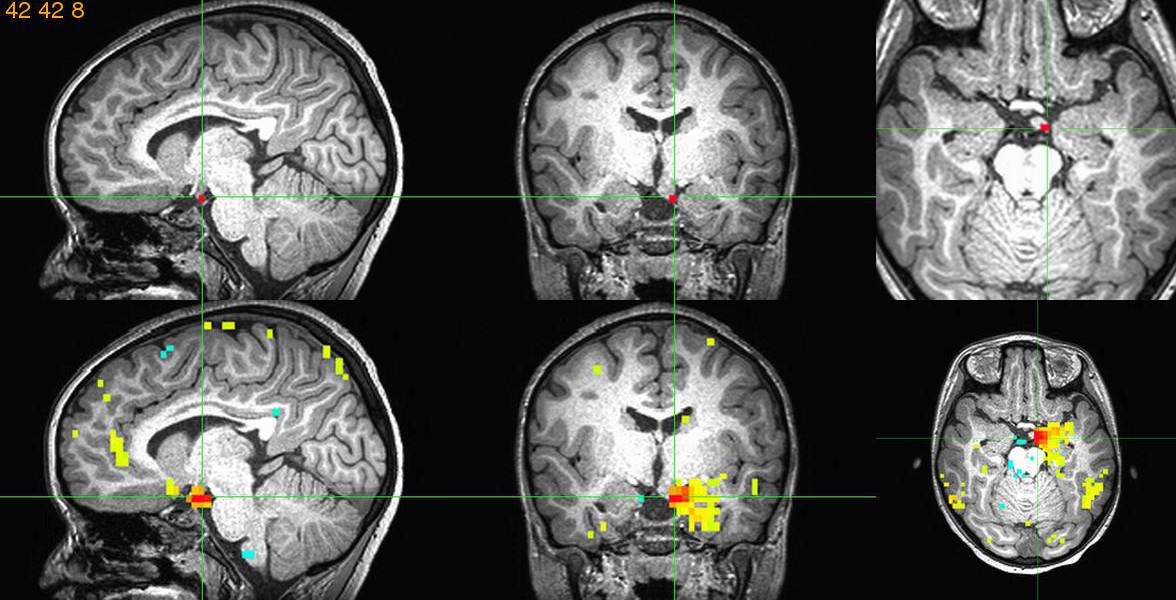 | 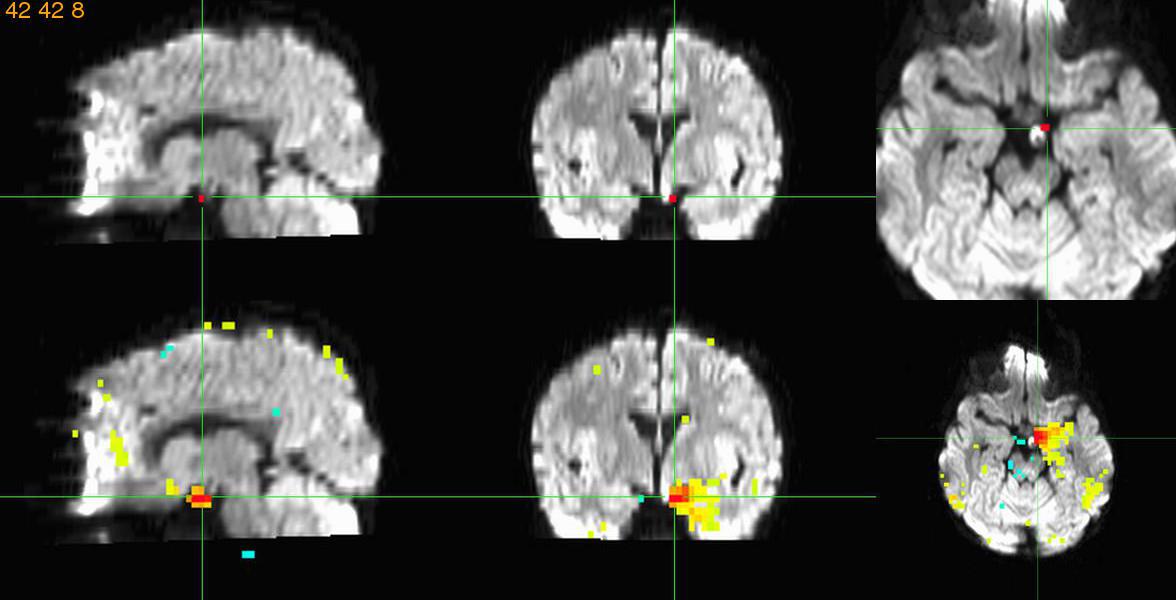 | 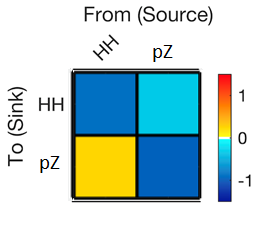 |
| P9-T5 | 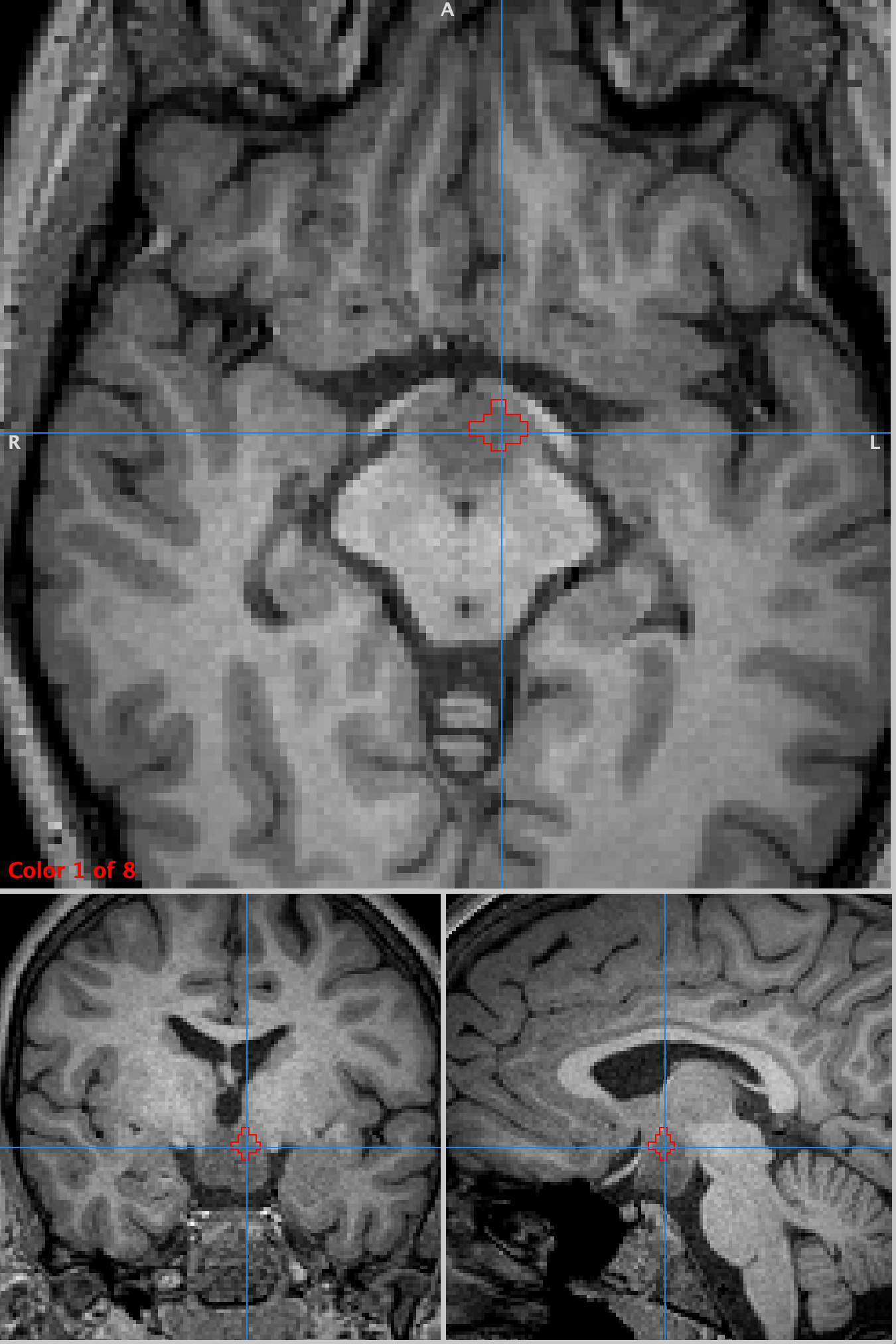 | 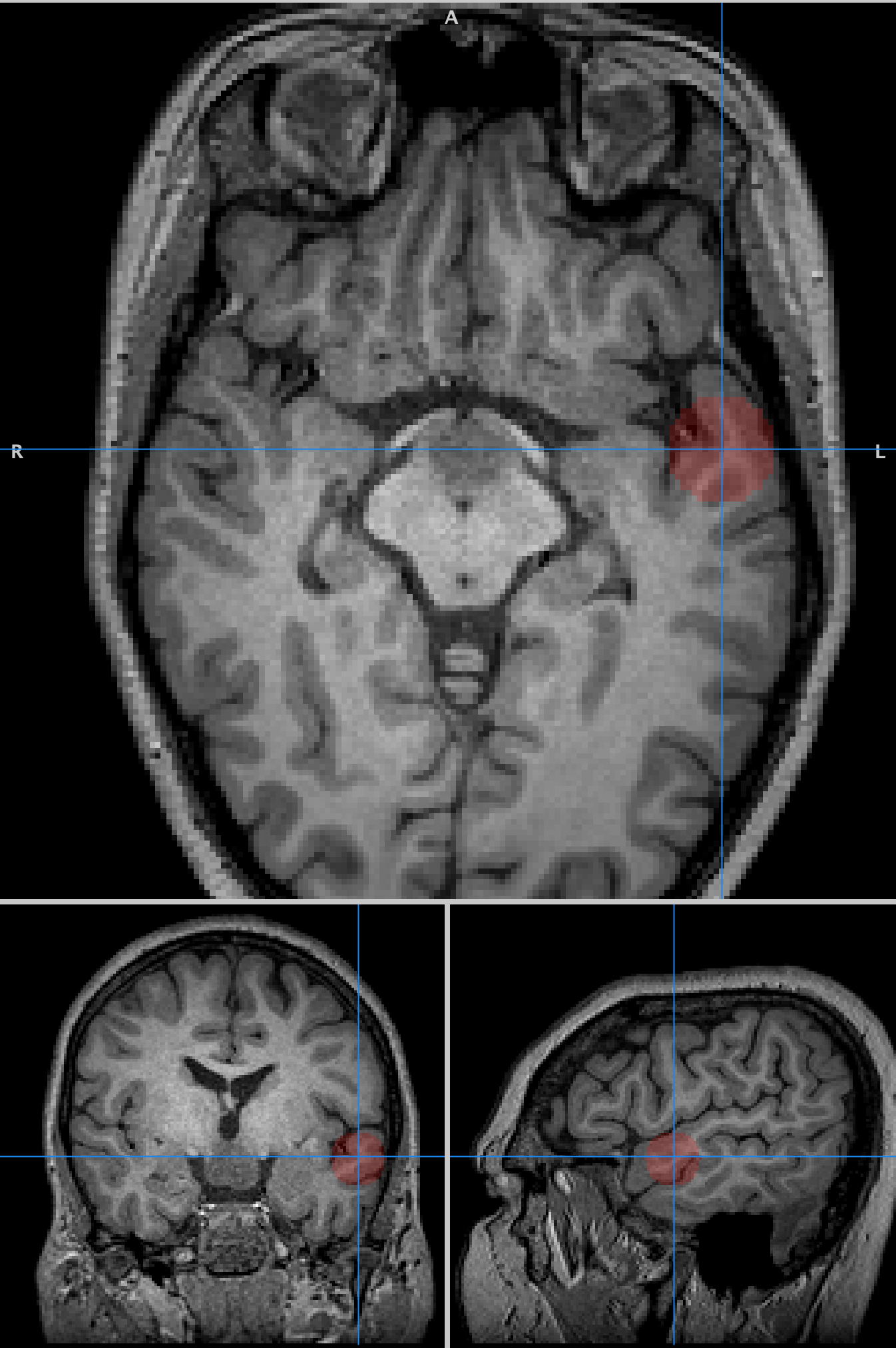 | 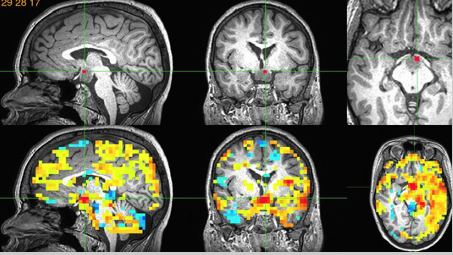 | 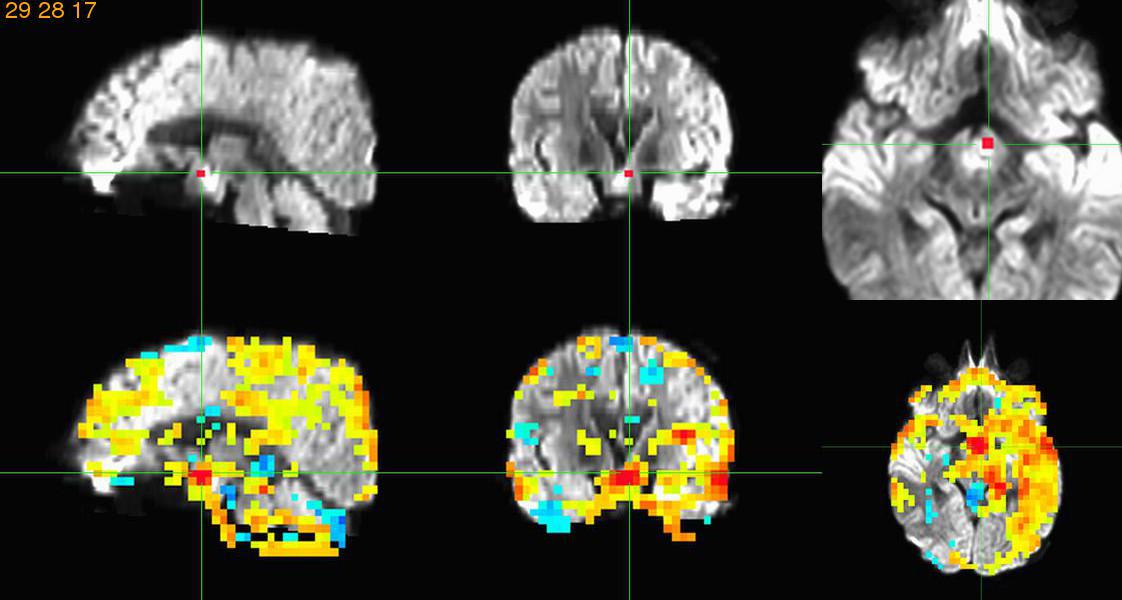 | 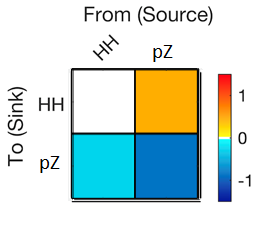 |
| P10-T6 | 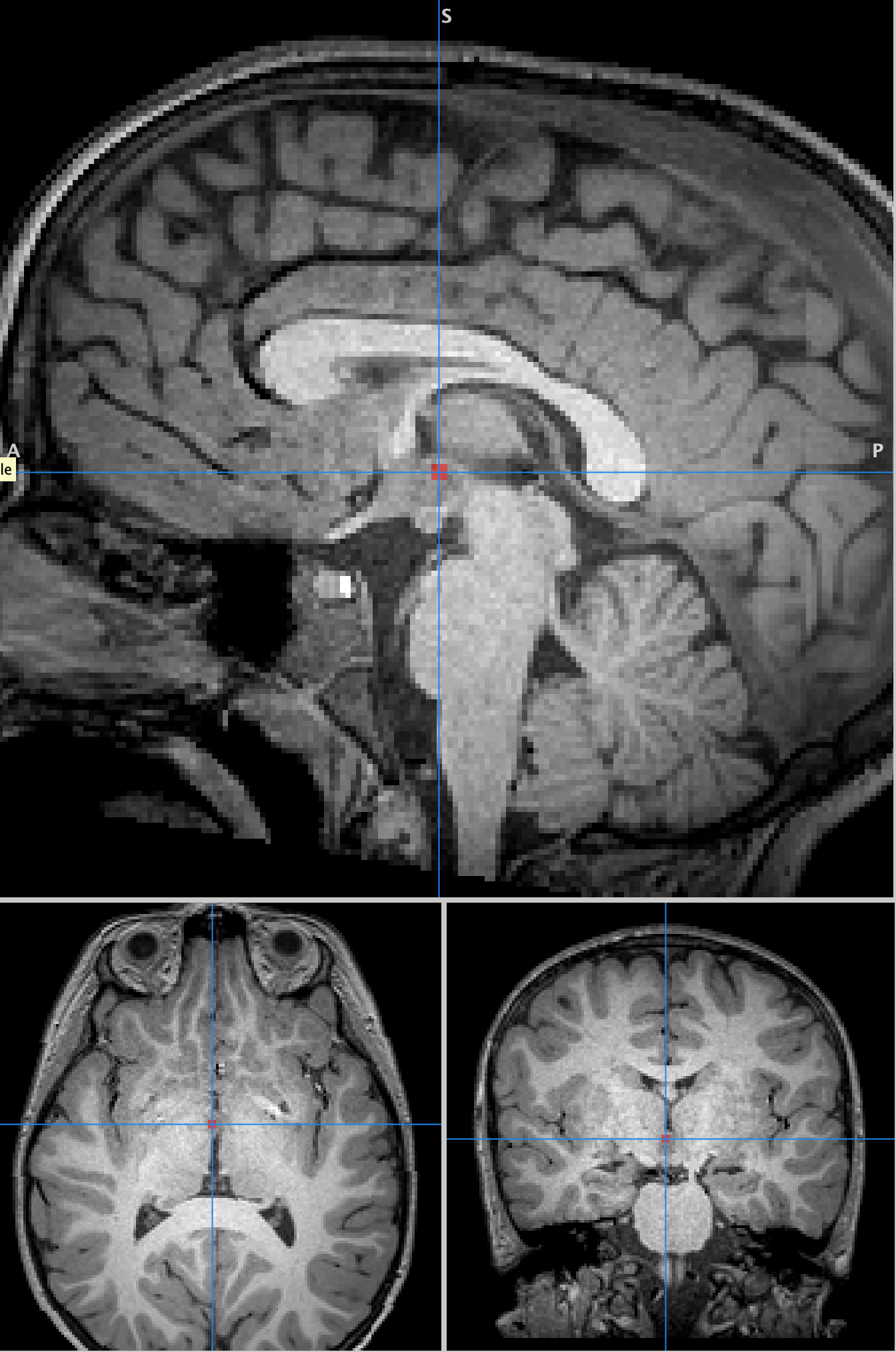 | 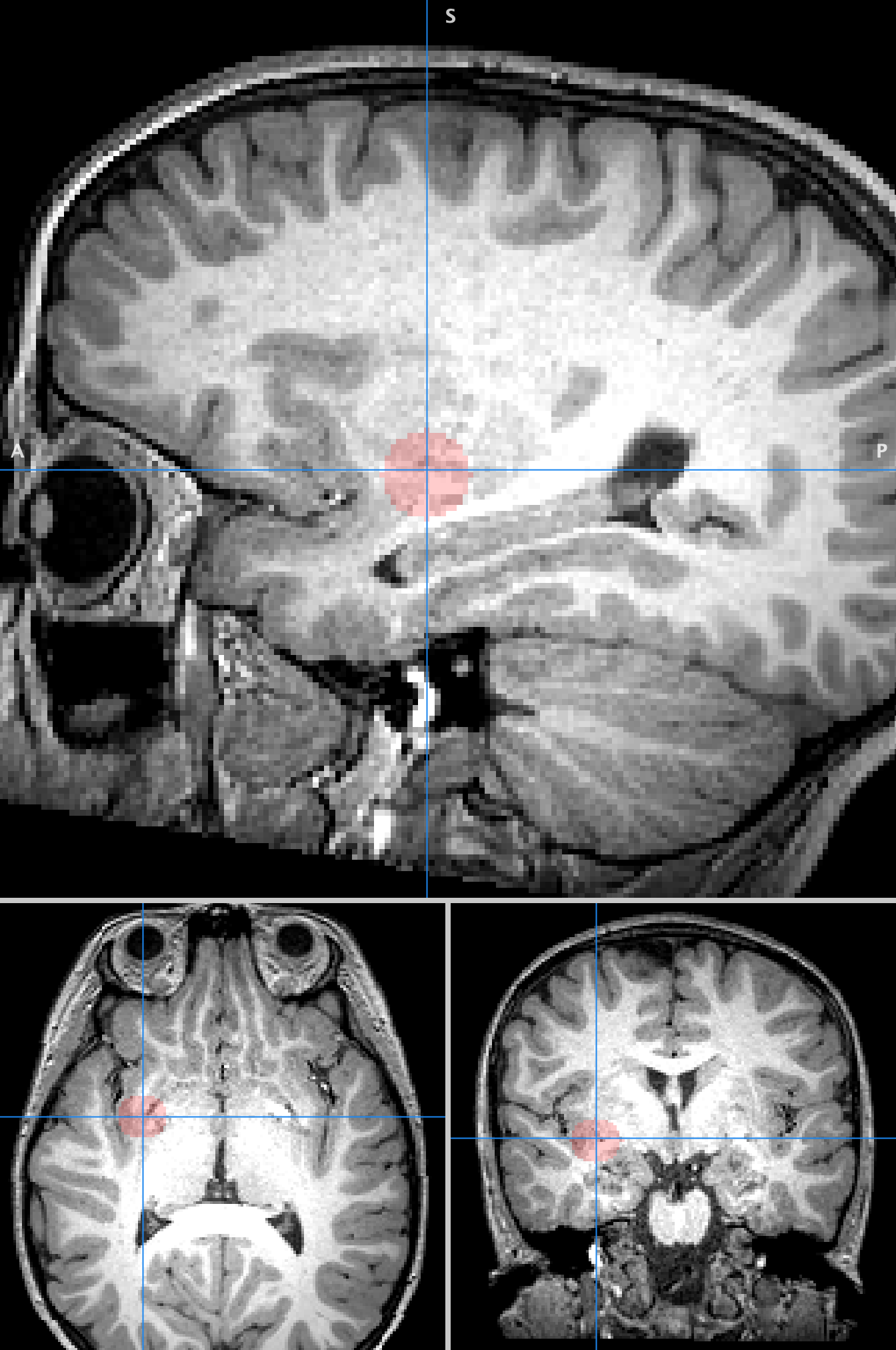 | 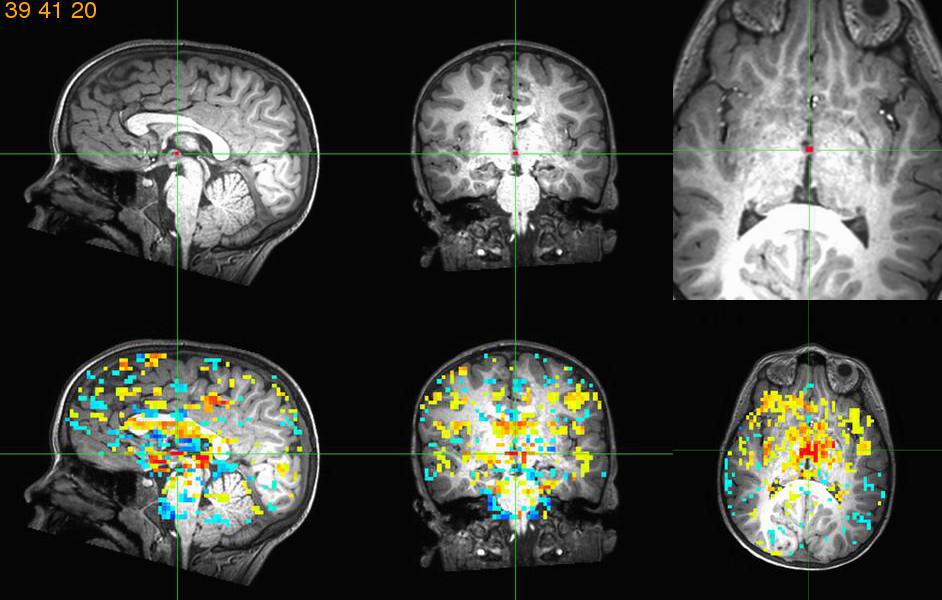 | 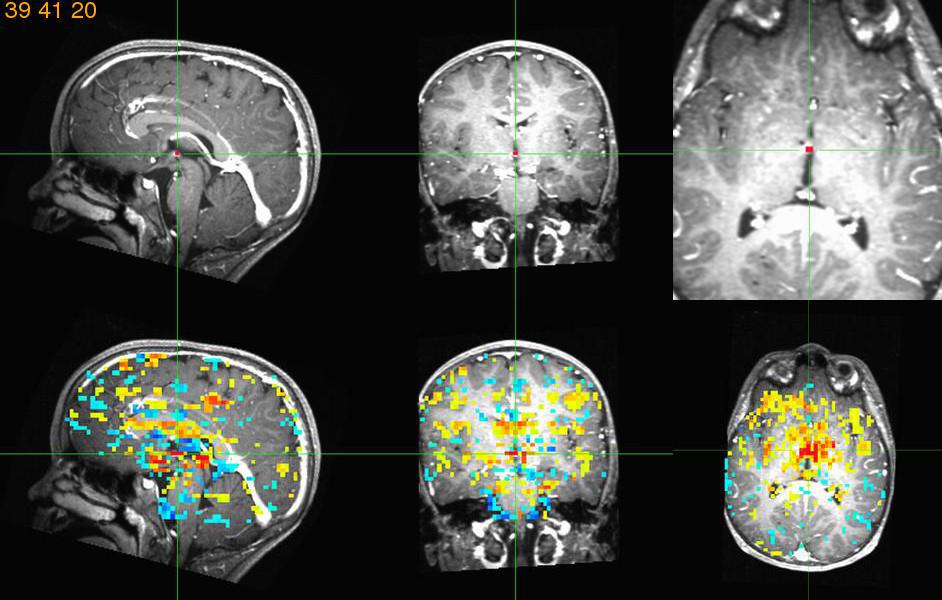 | 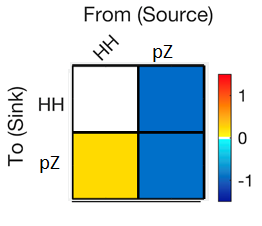 |
| P11-T7 | 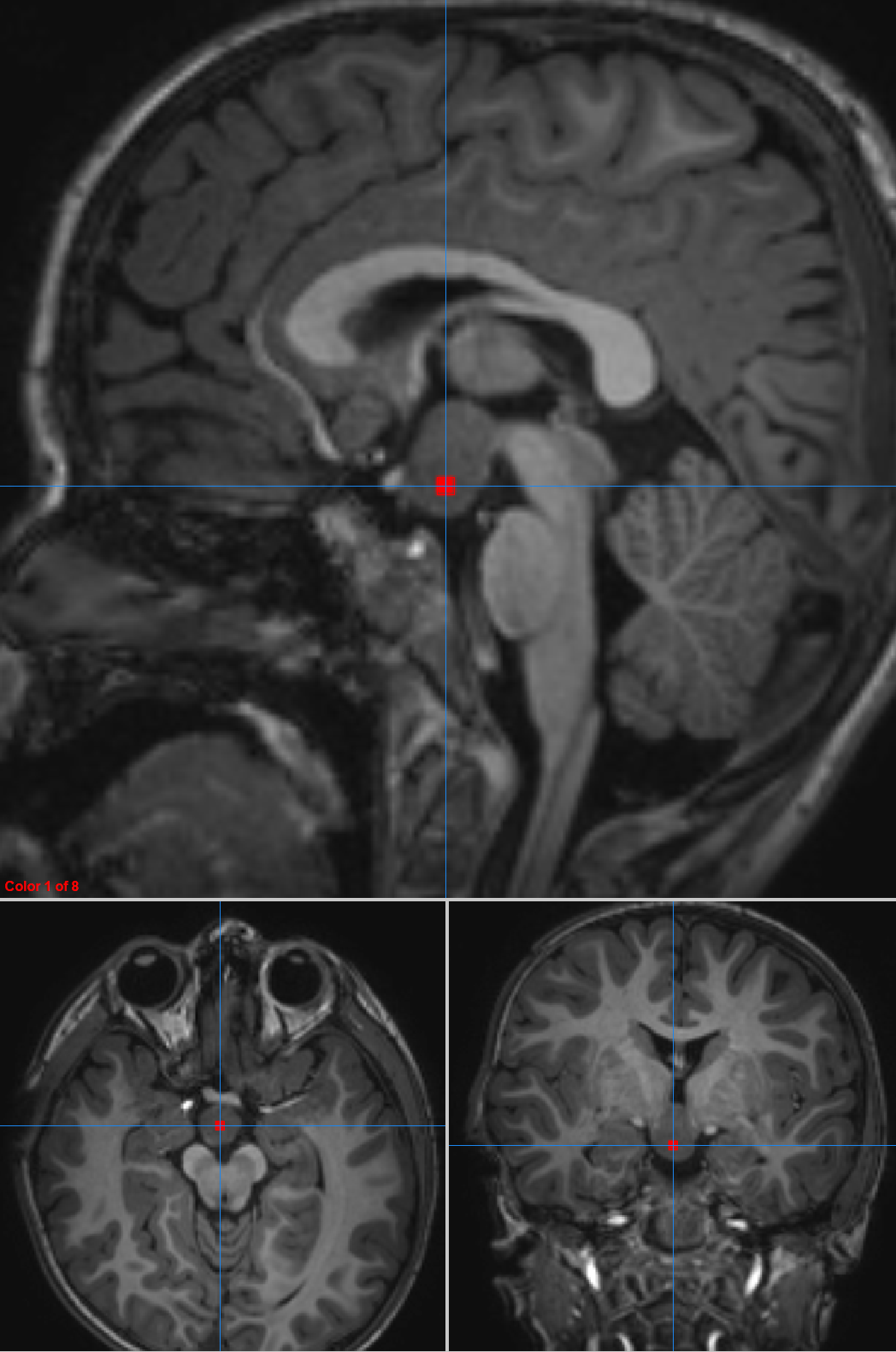 | 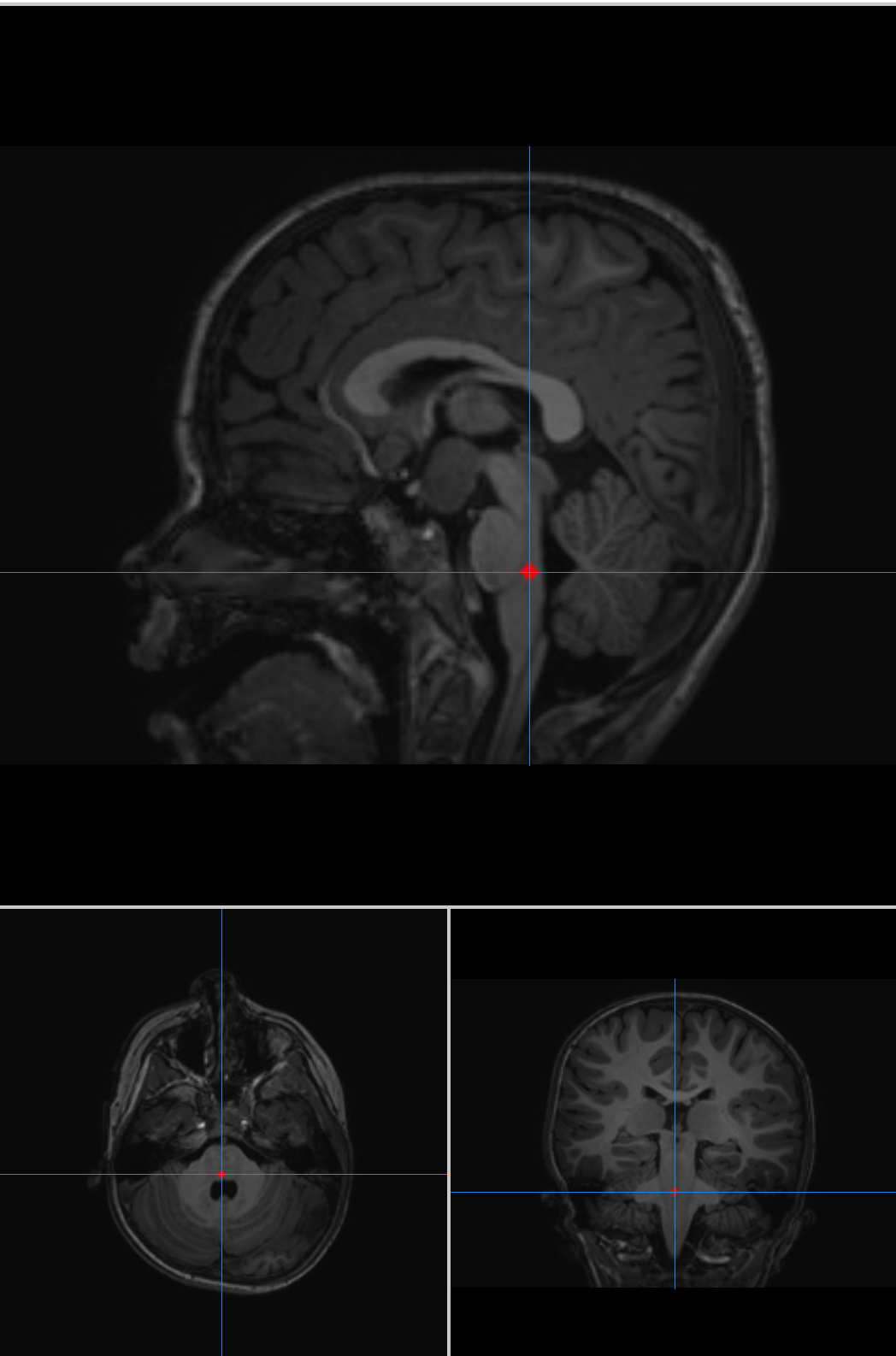 | 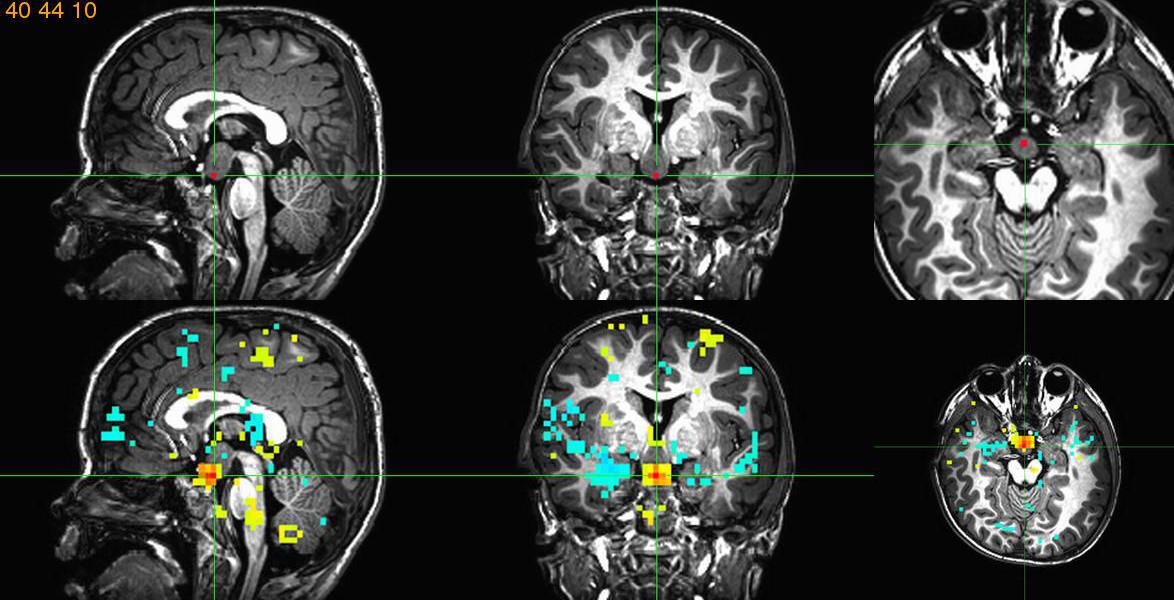 | 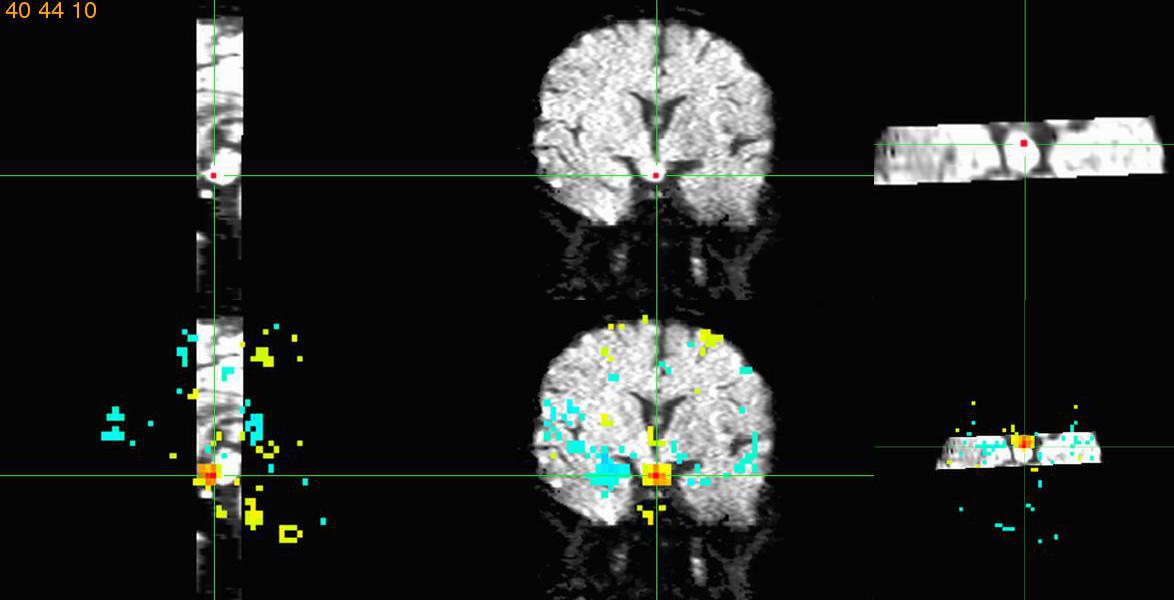 | 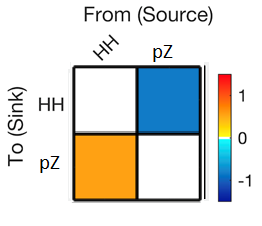 |
| P12-T8 | 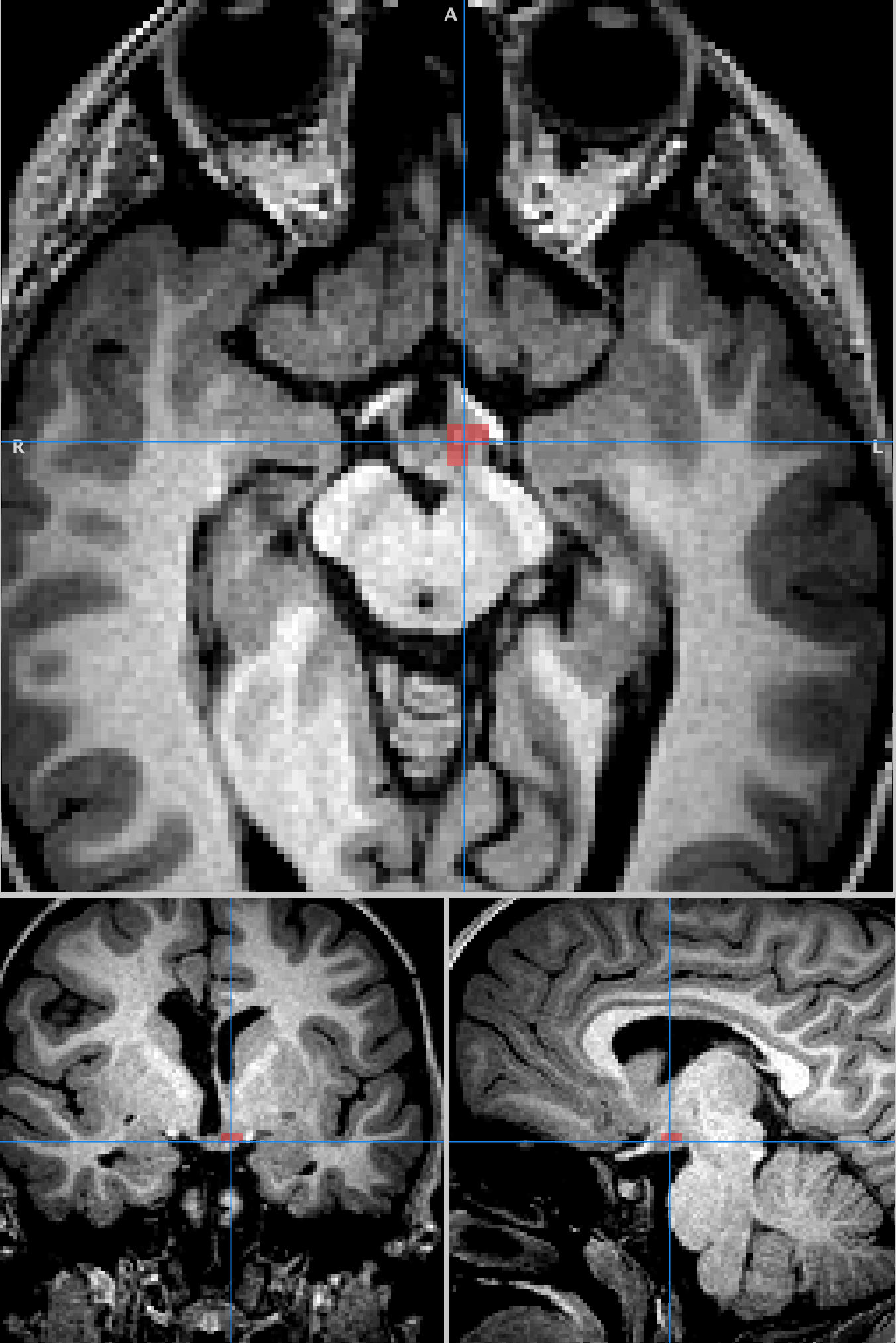 | 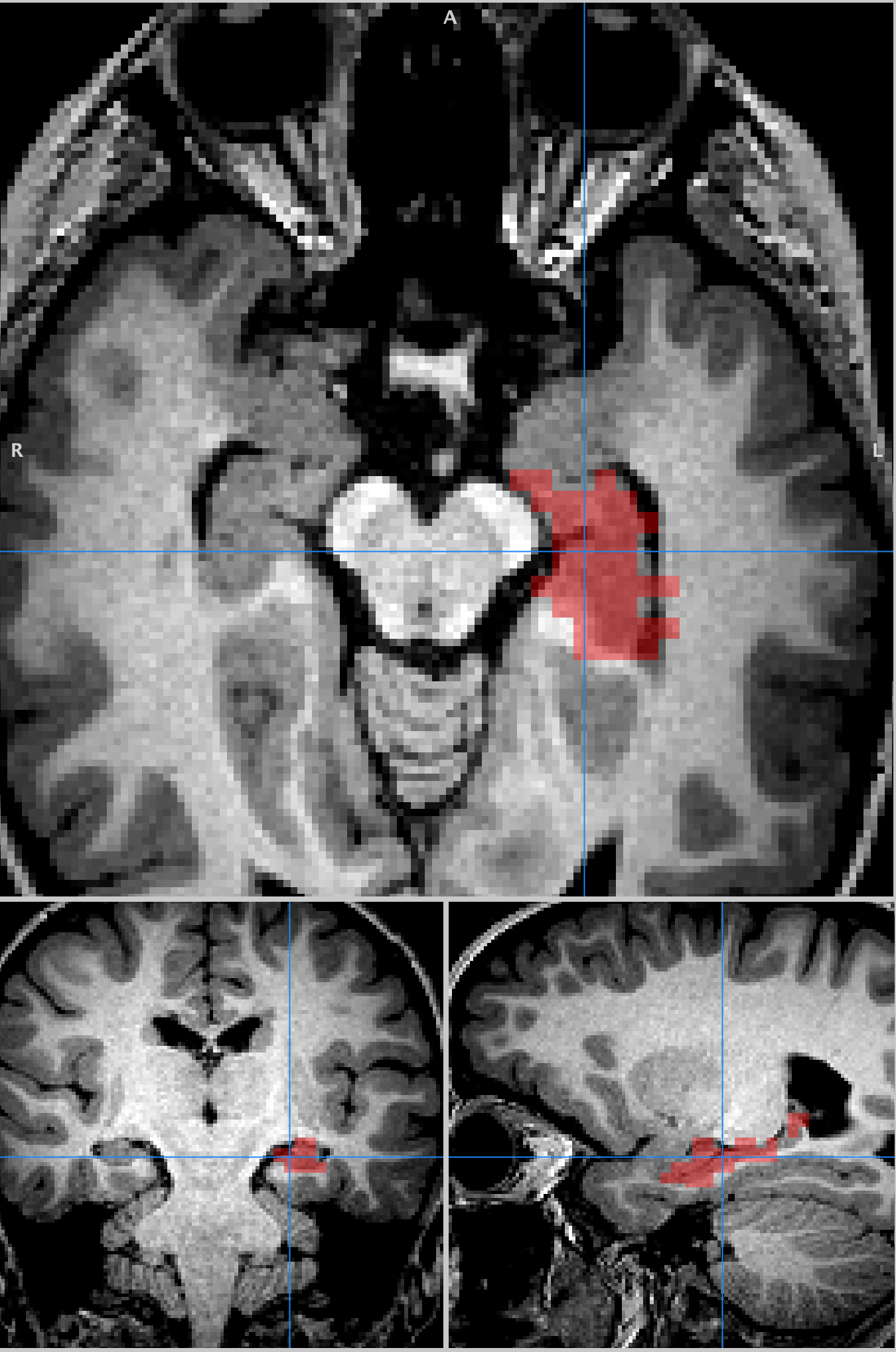 | 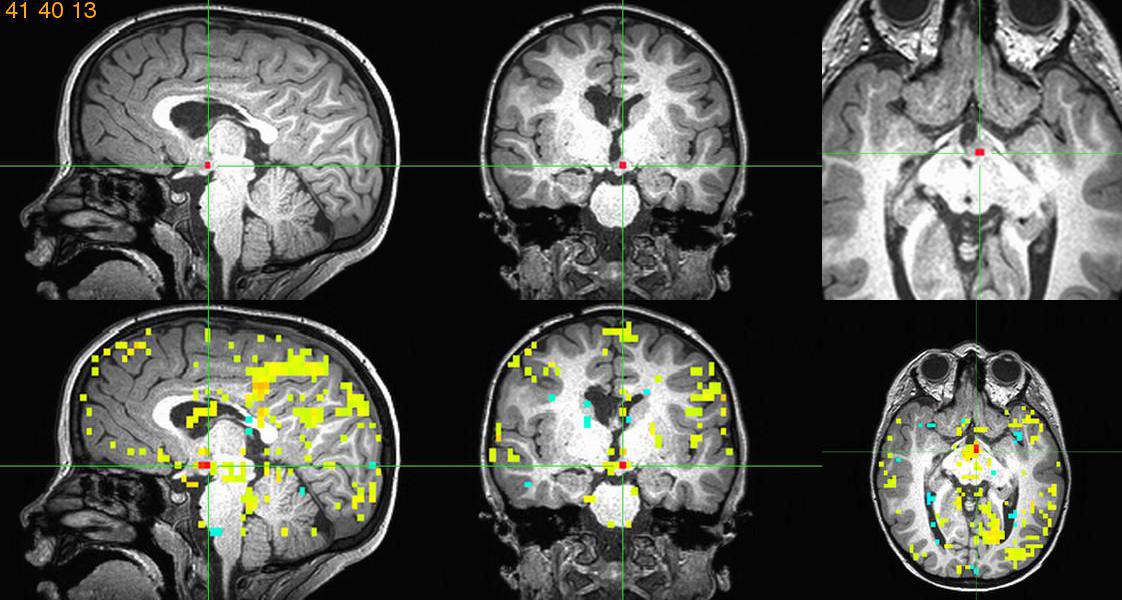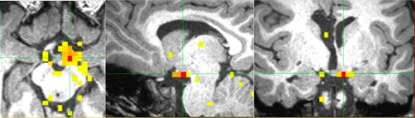 | 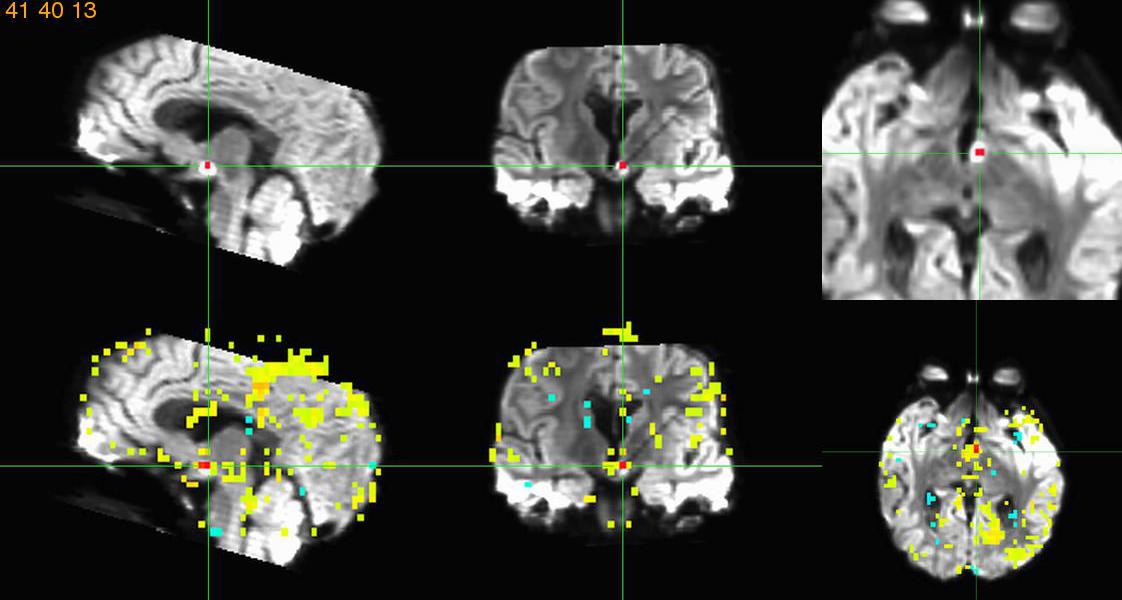 | 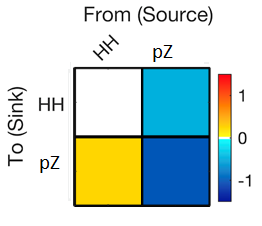 |
| P13-T9 | **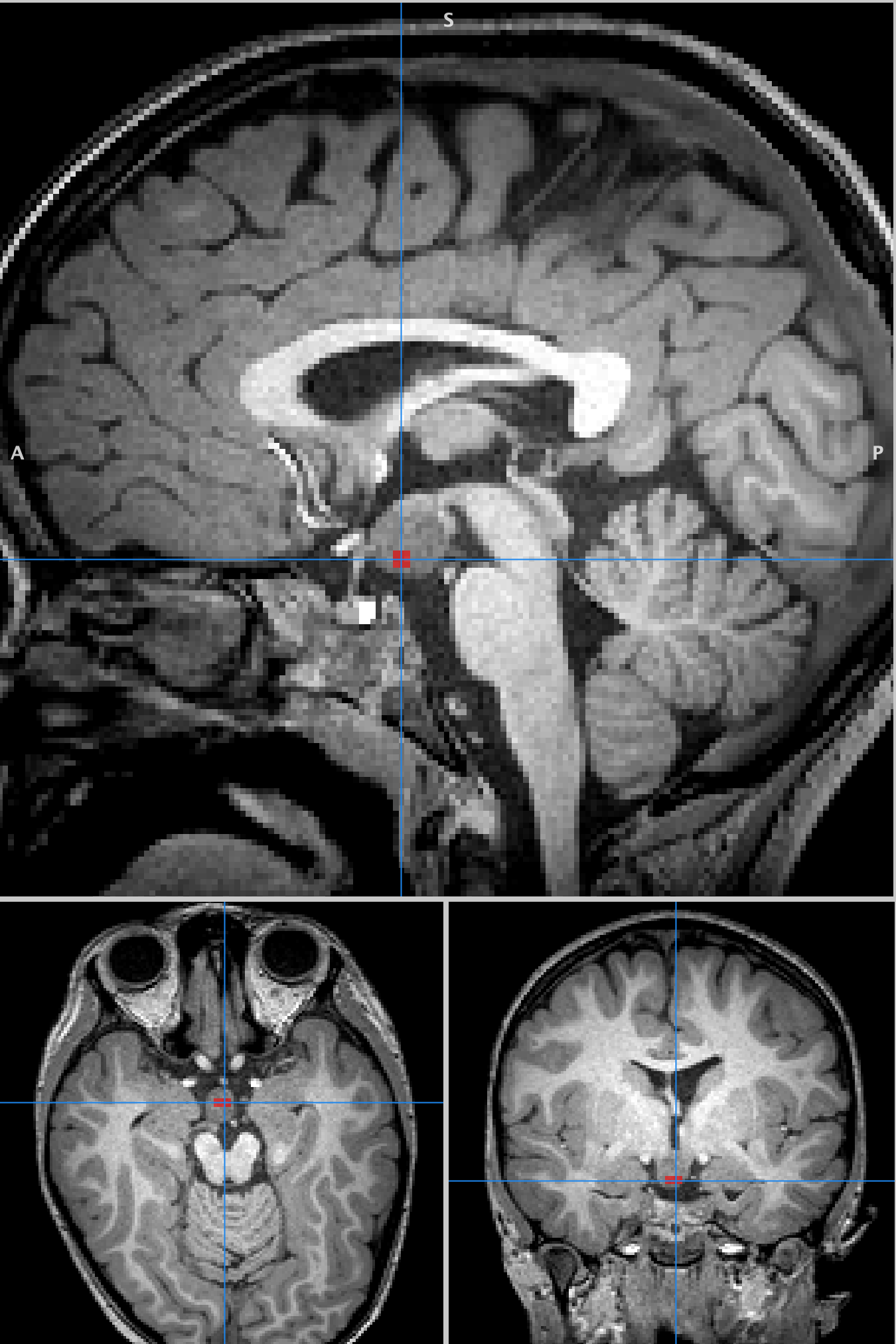** | 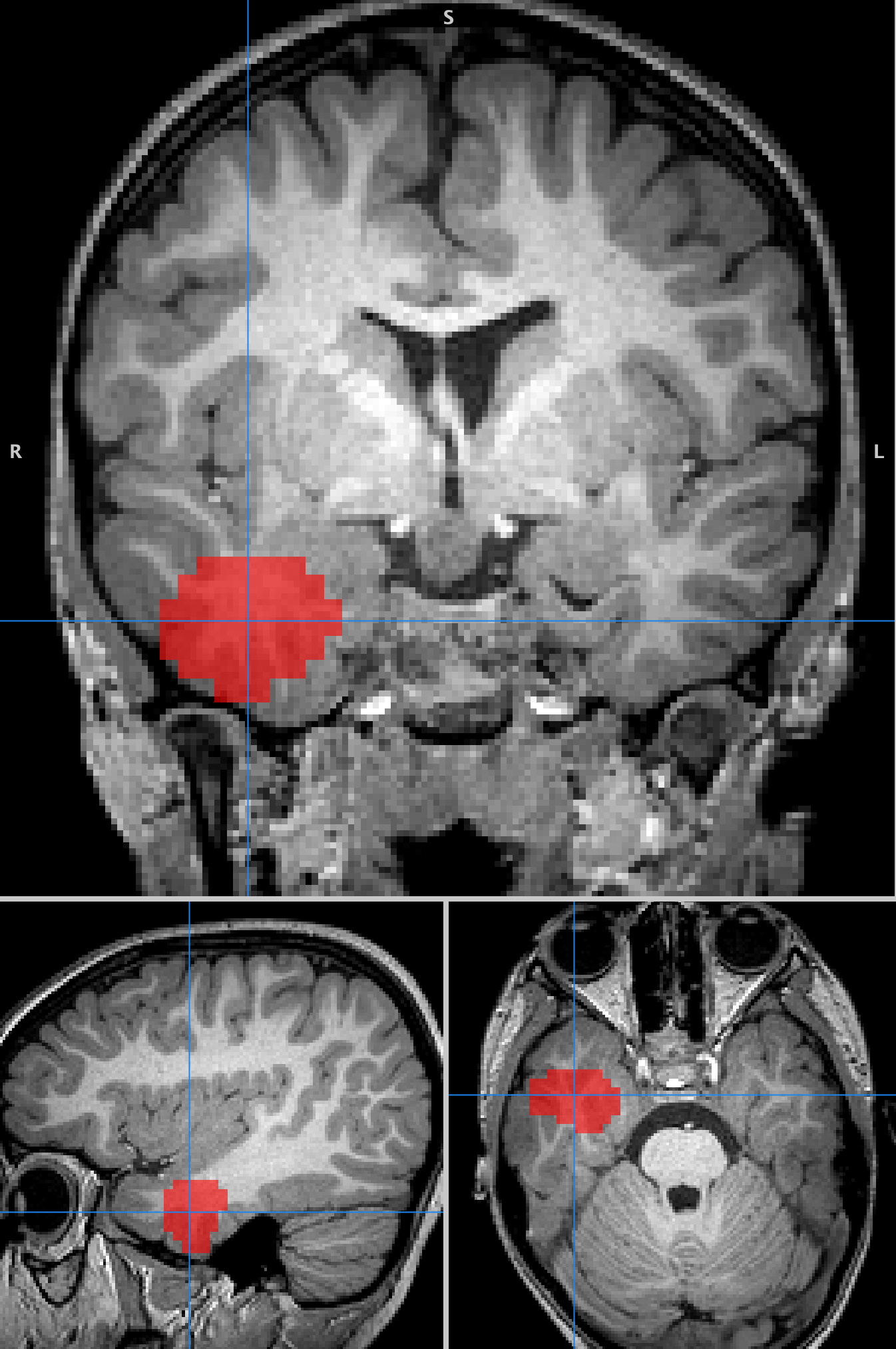 | 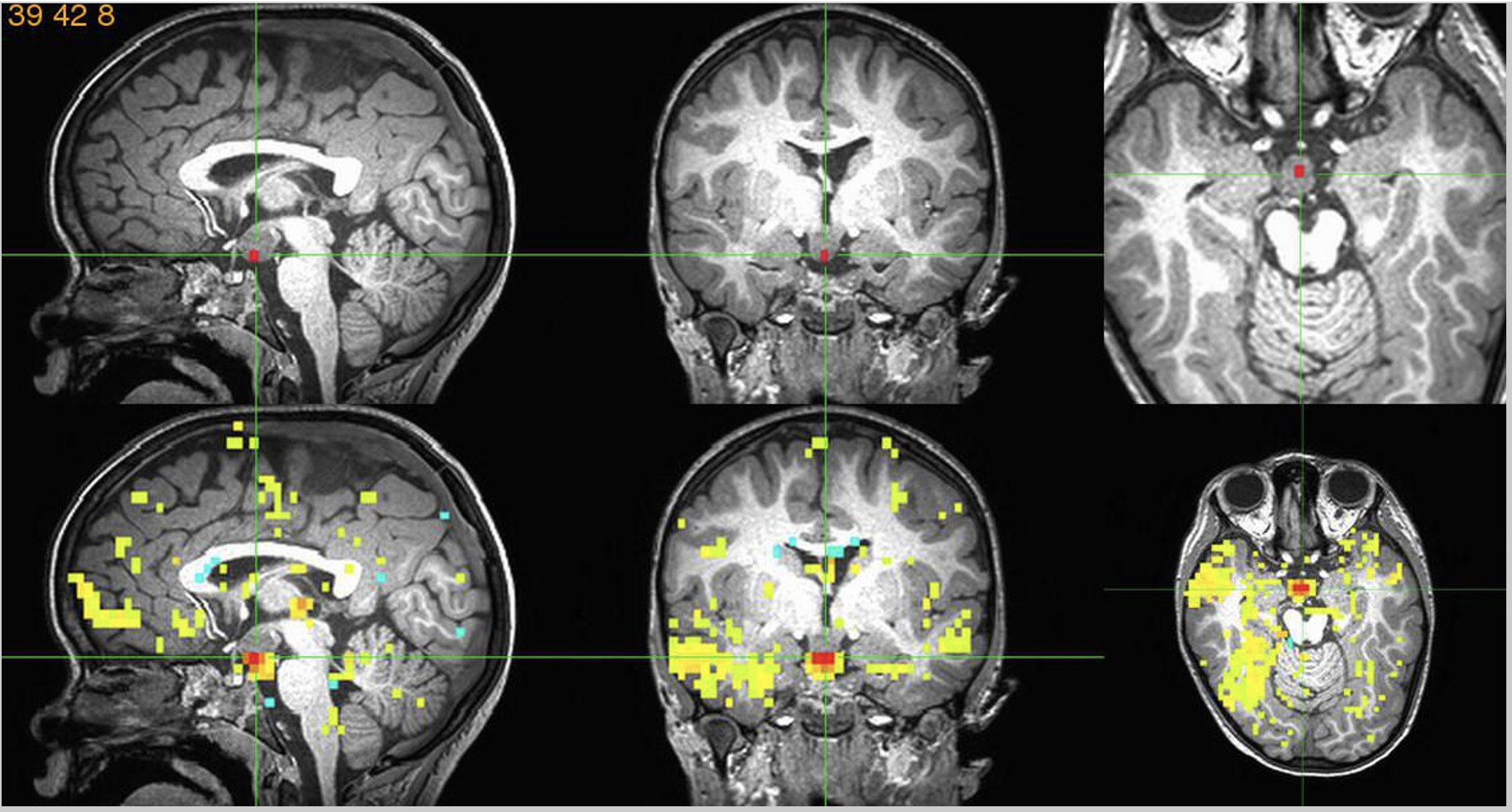 | 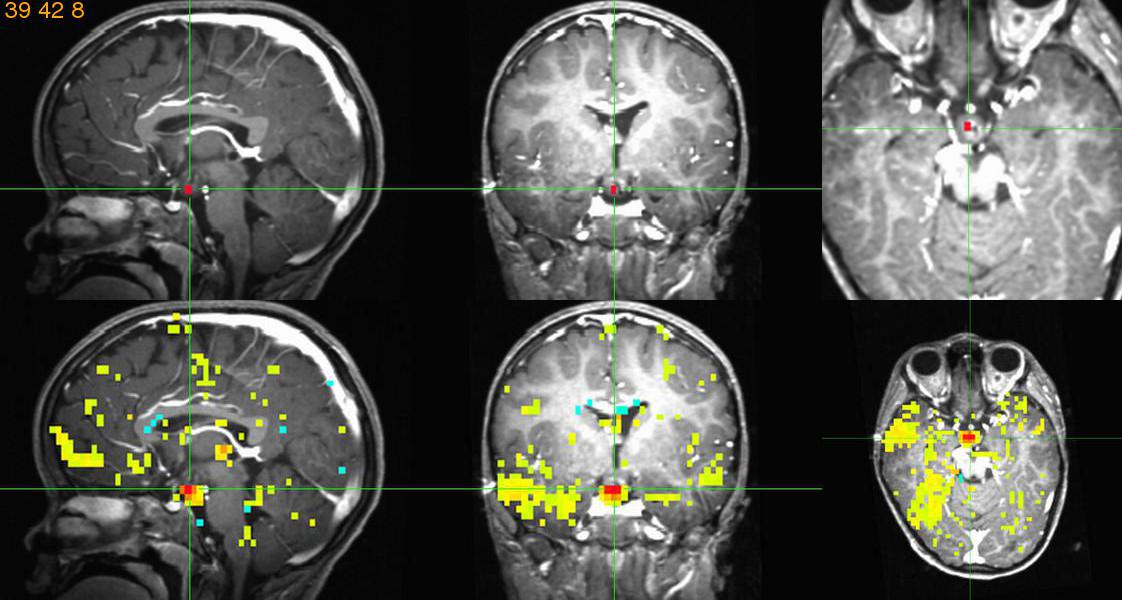 | 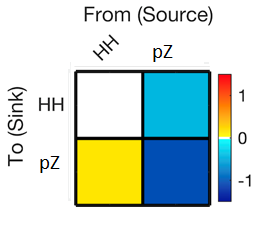 |
| P14-T10 | 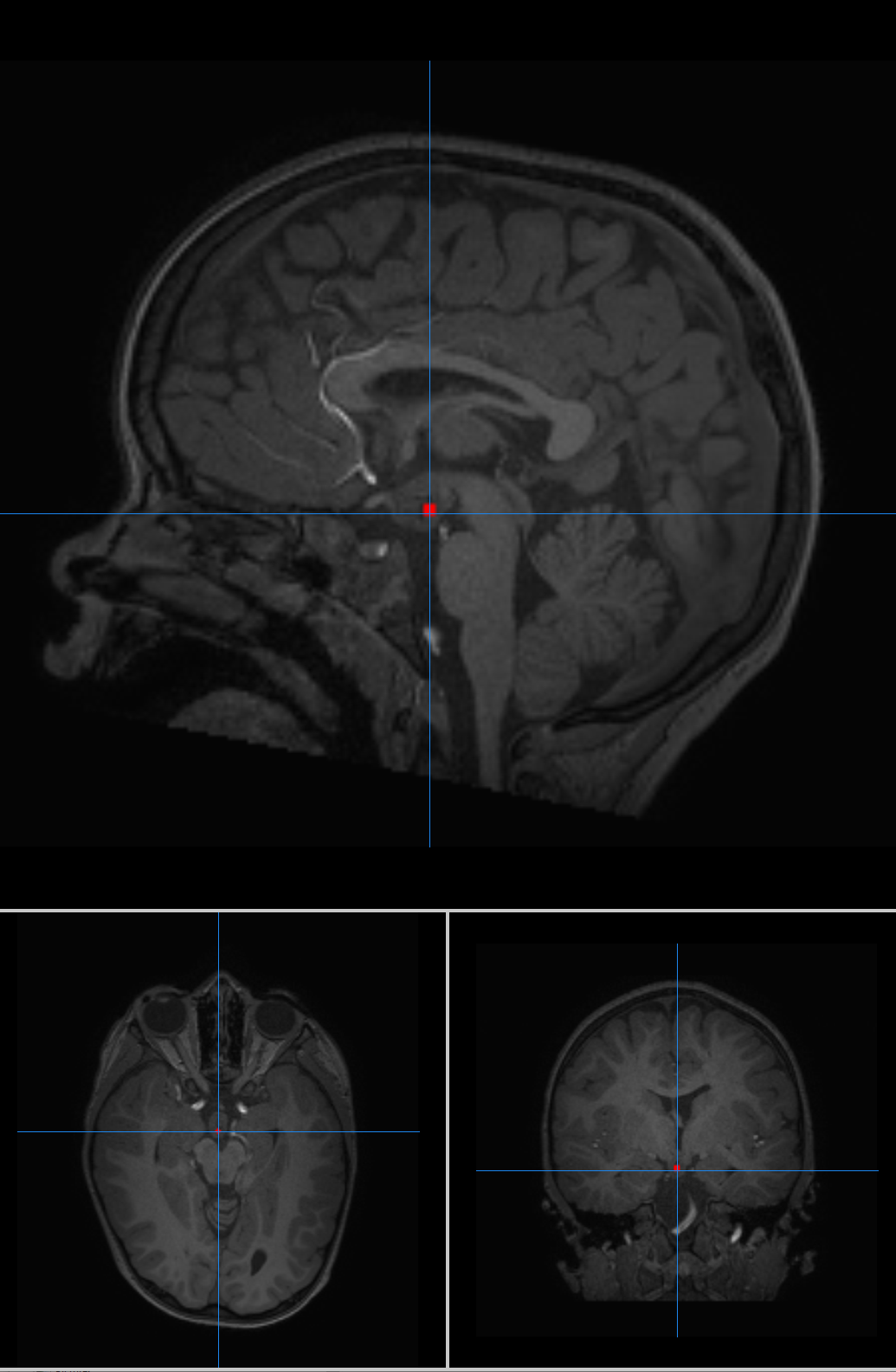 | 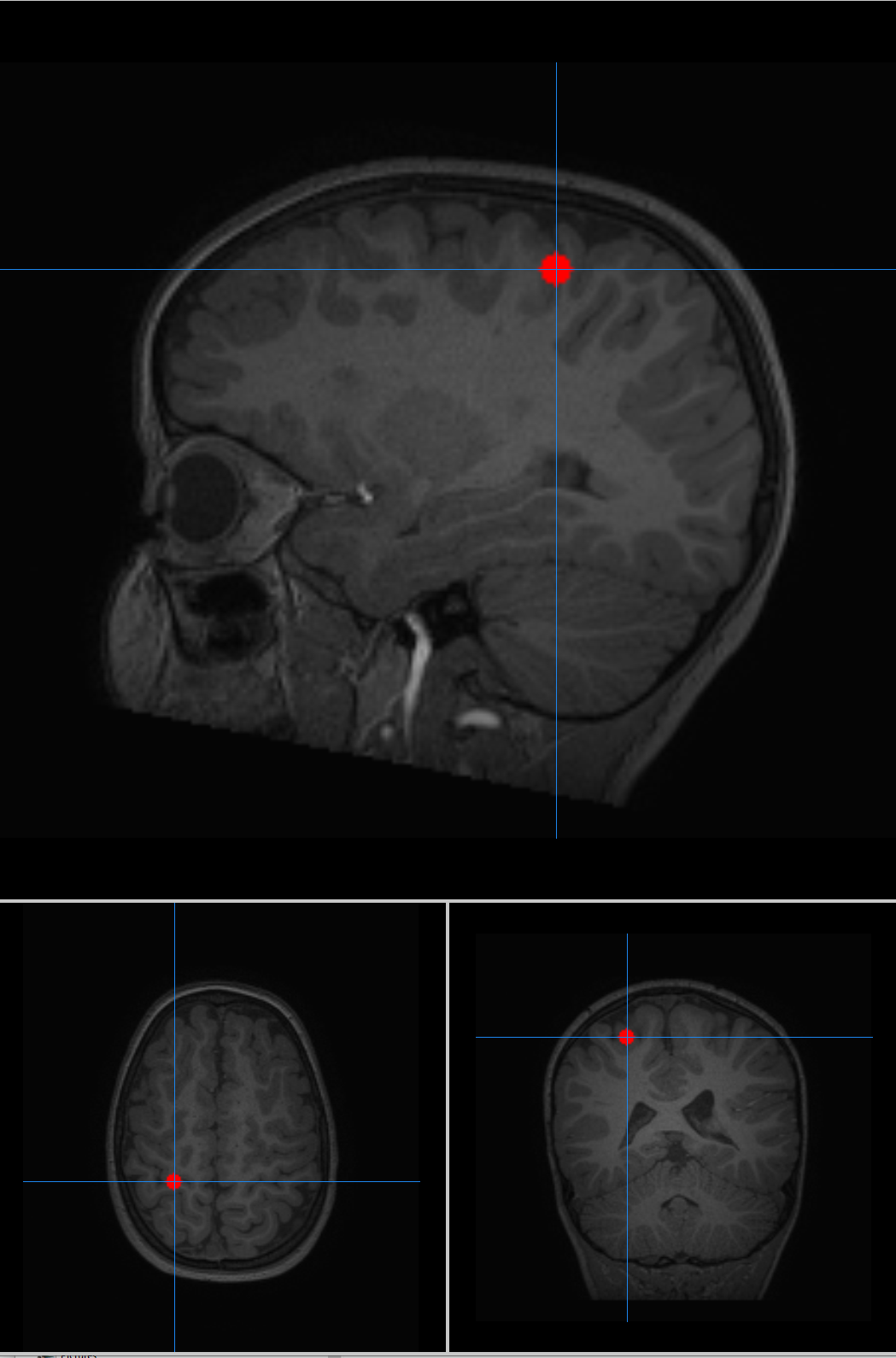 | 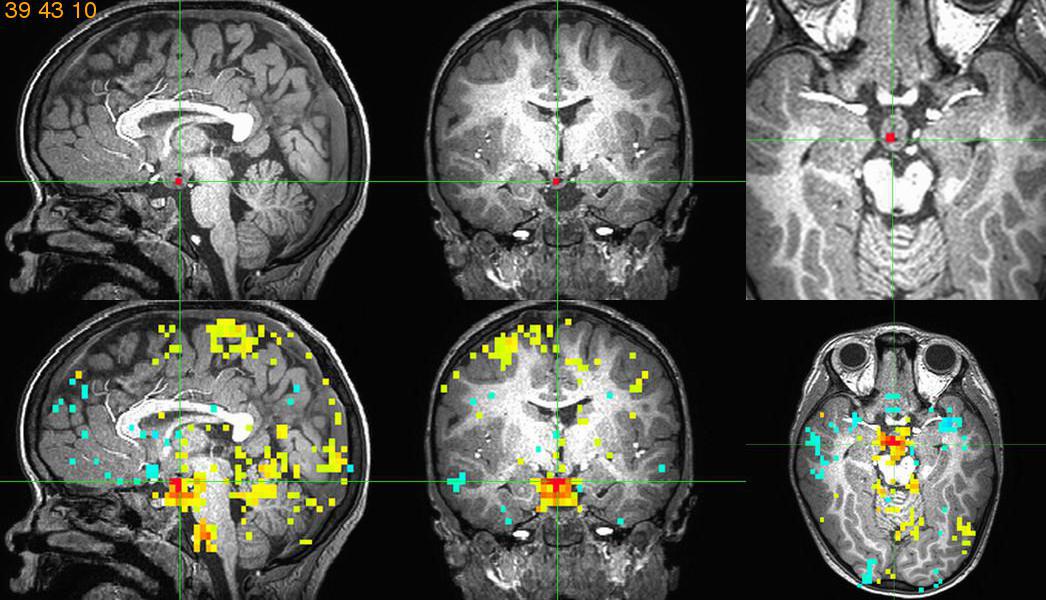 | 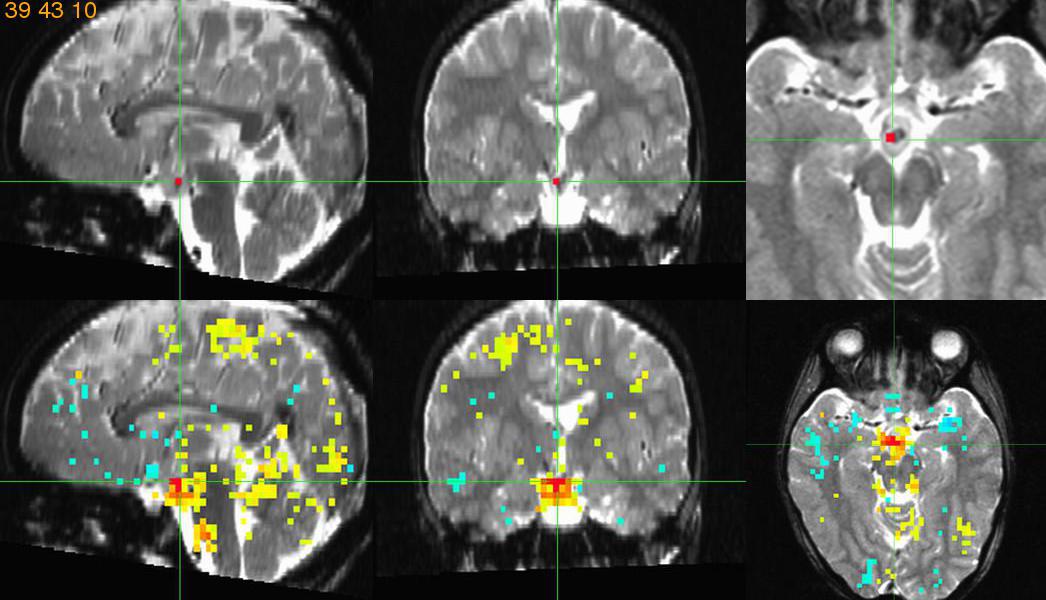  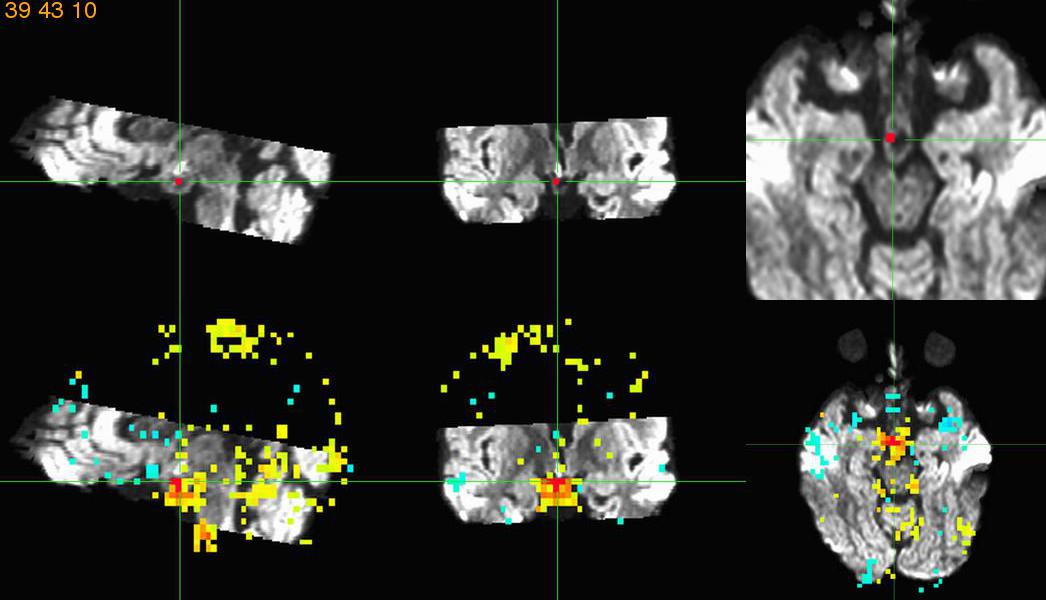 | 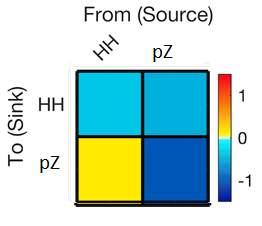 |
| P15-T11 | 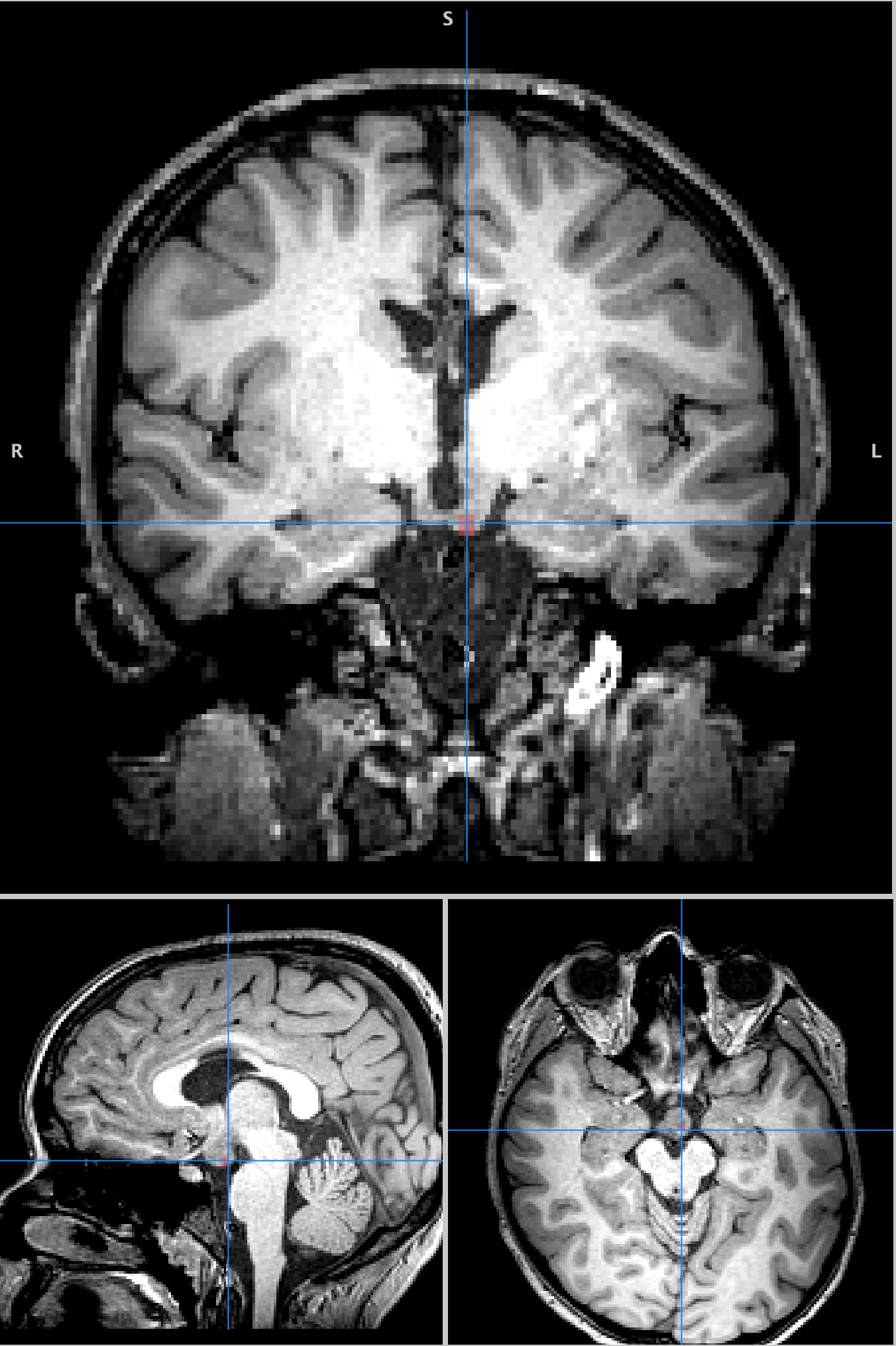 | 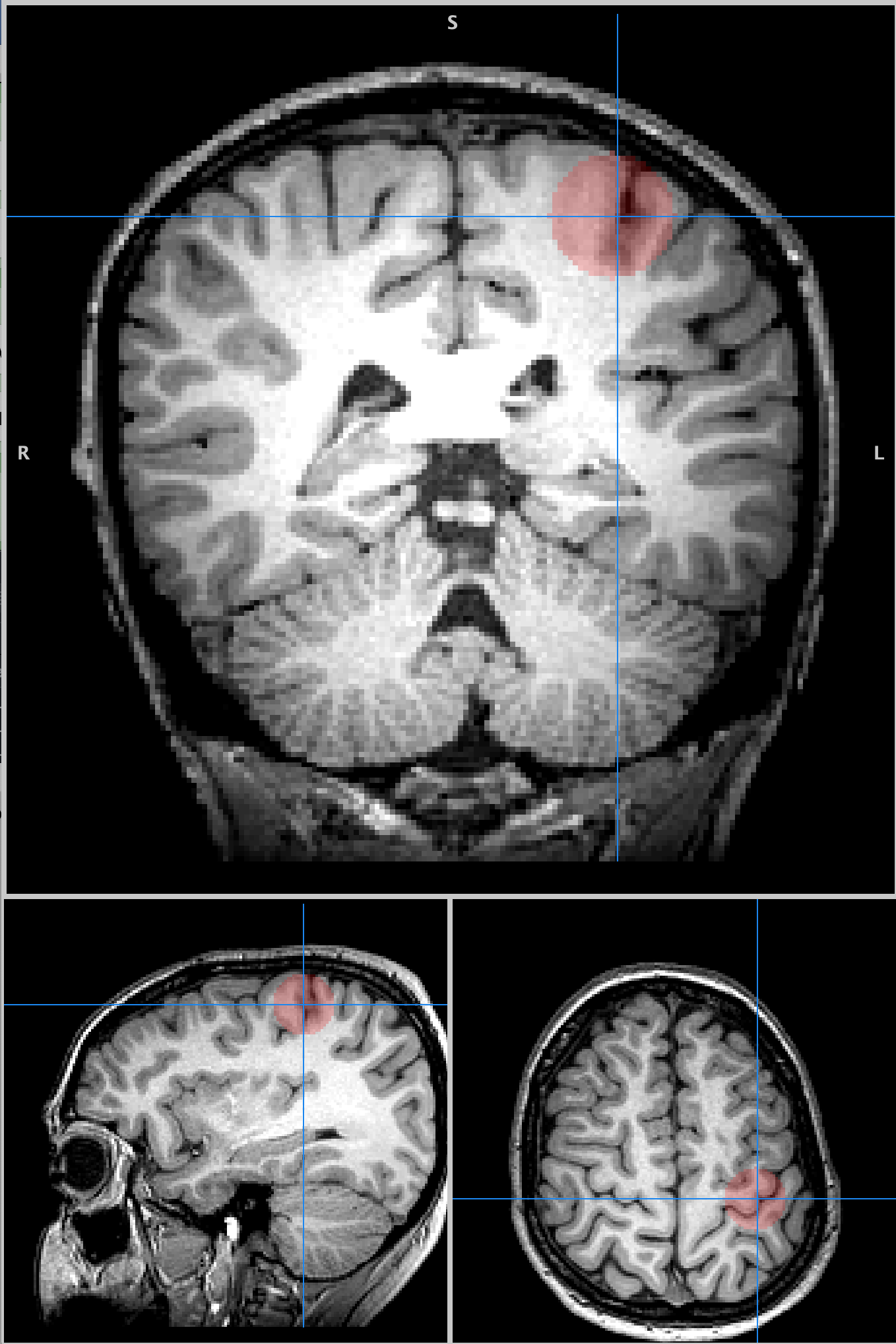 | 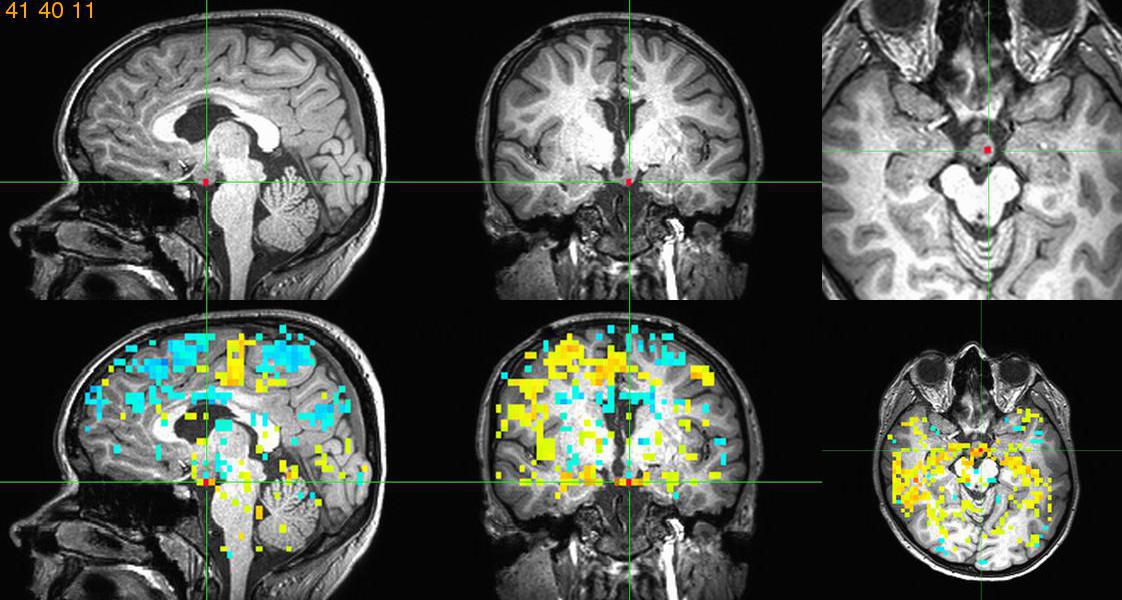 | 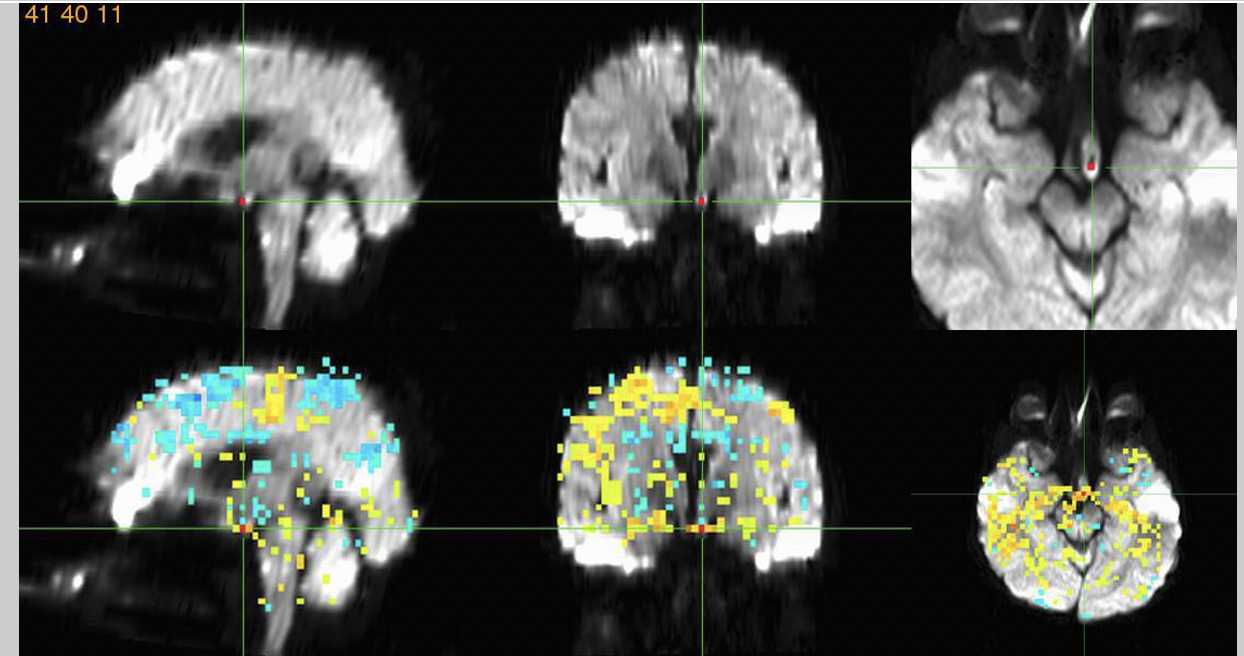  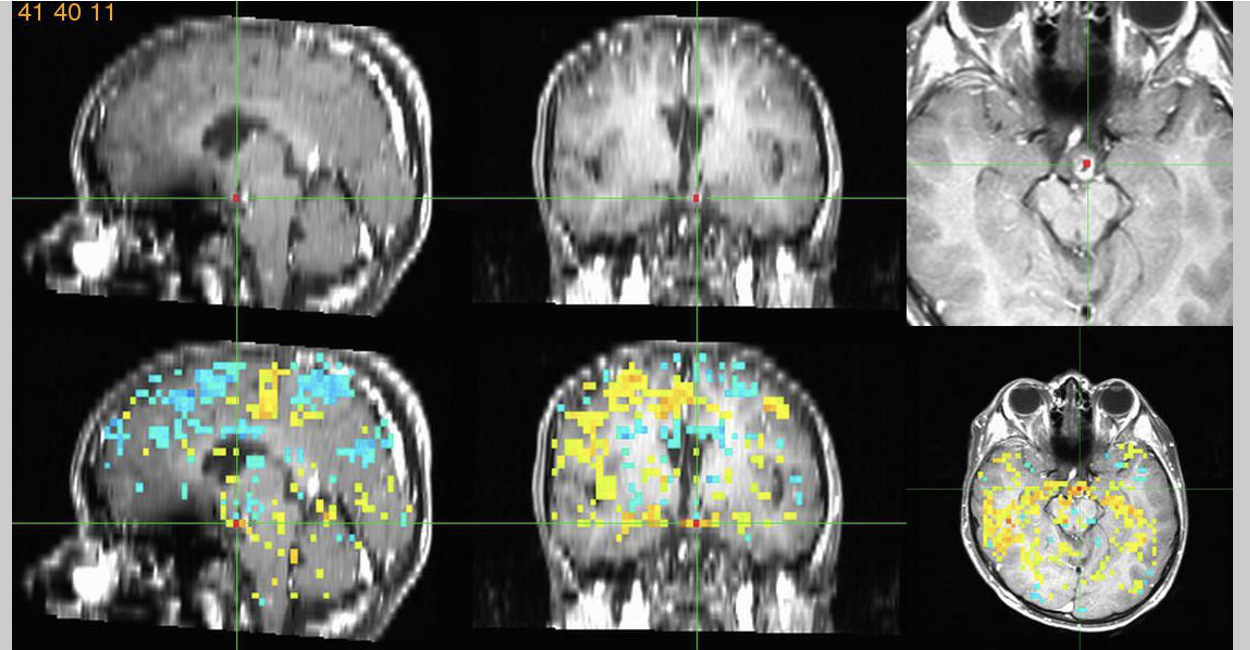 | 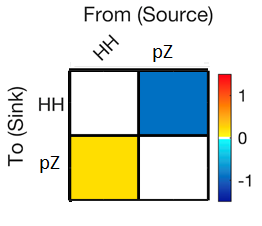 |
| P16-T12 | 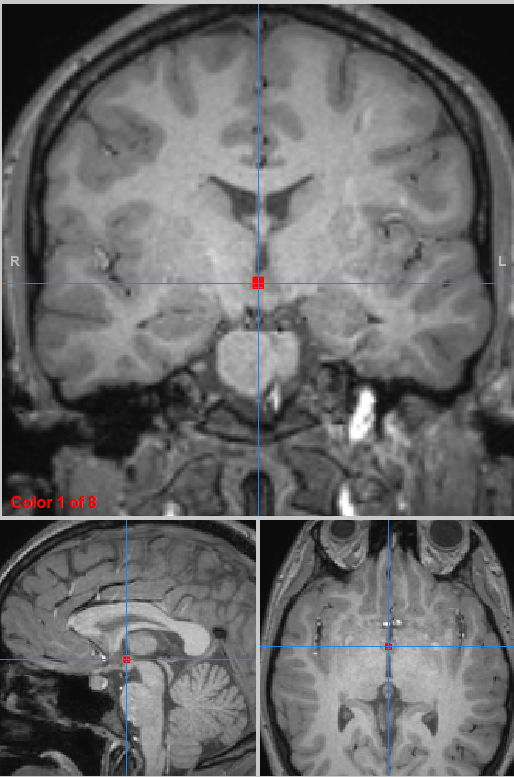 | 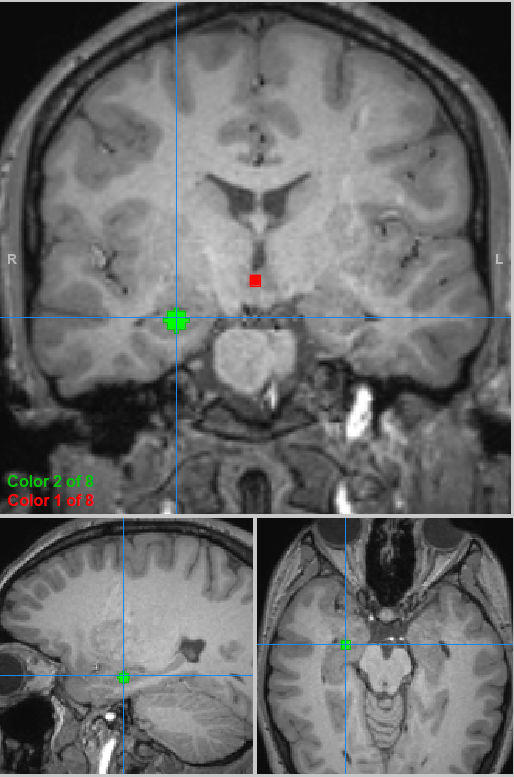 | 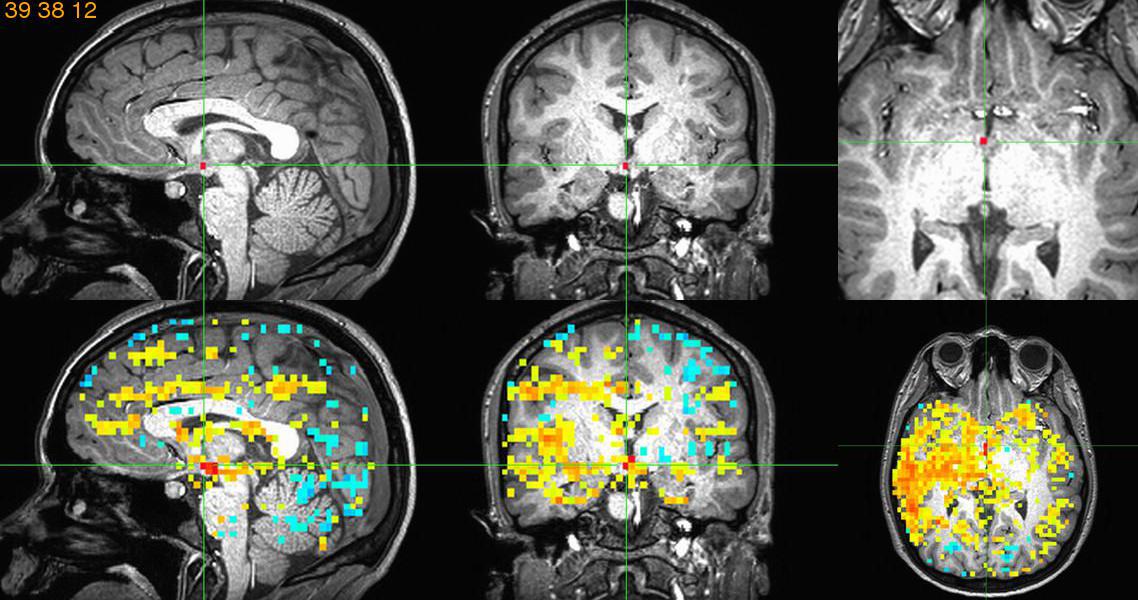 | 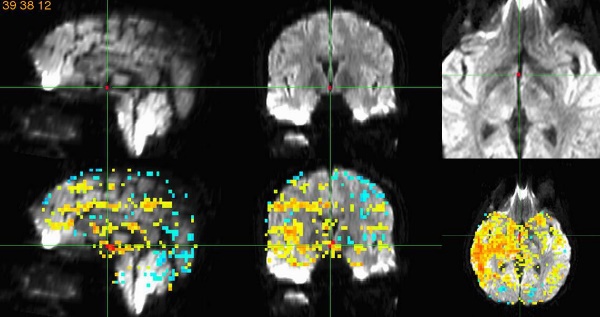 | 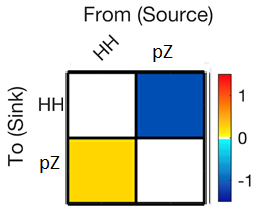 |
| P17-T13 | 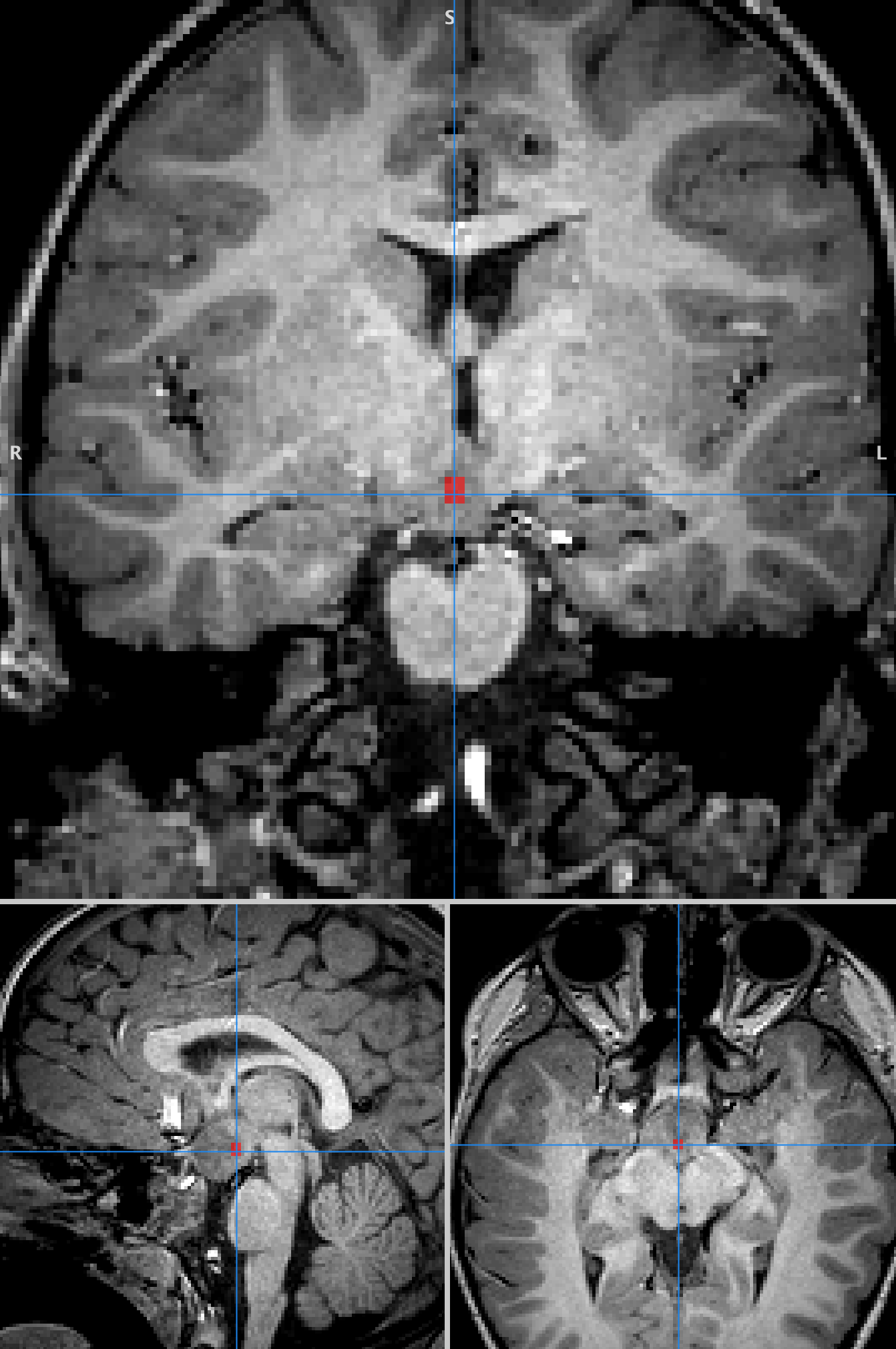 | 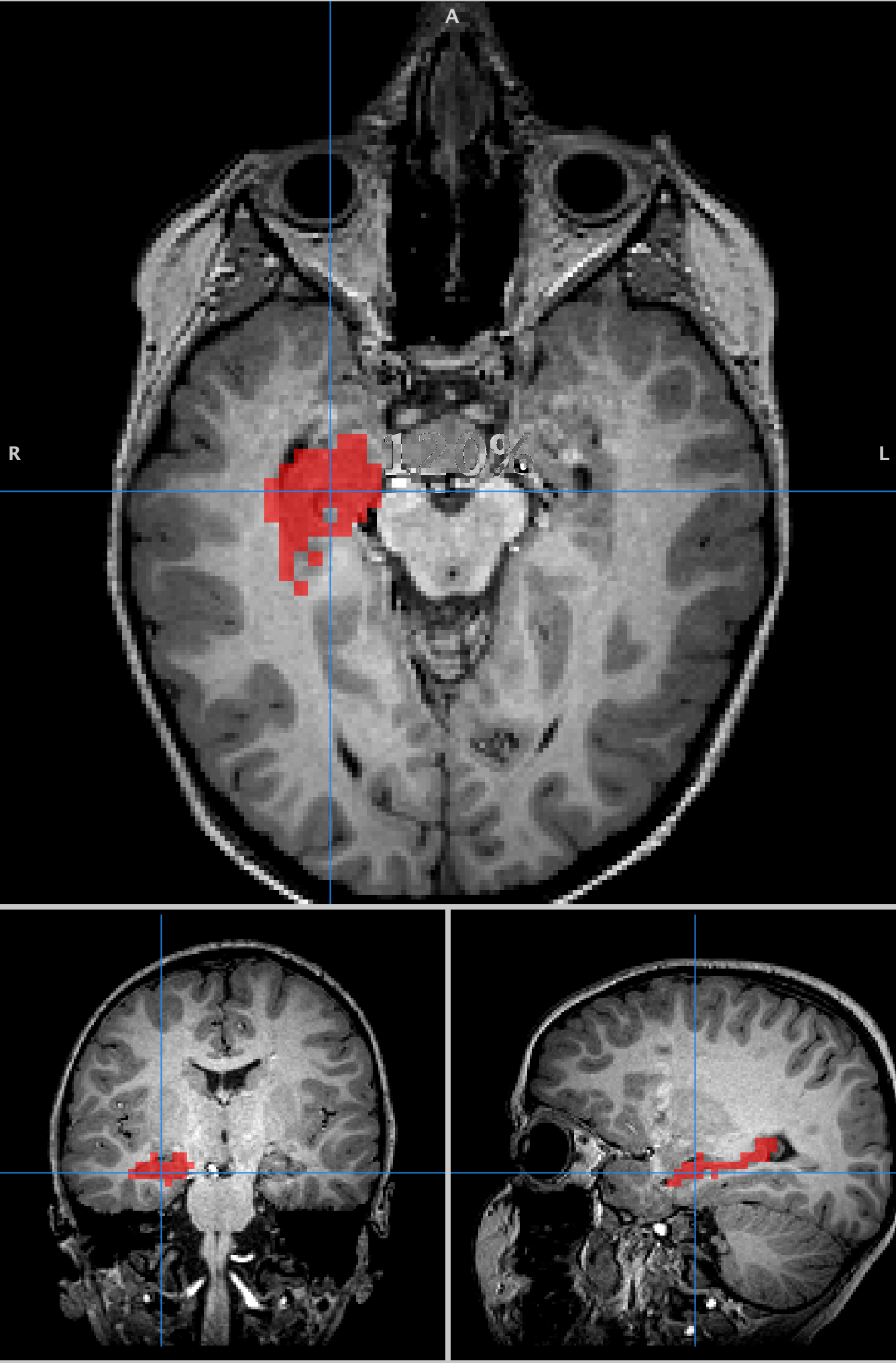 | 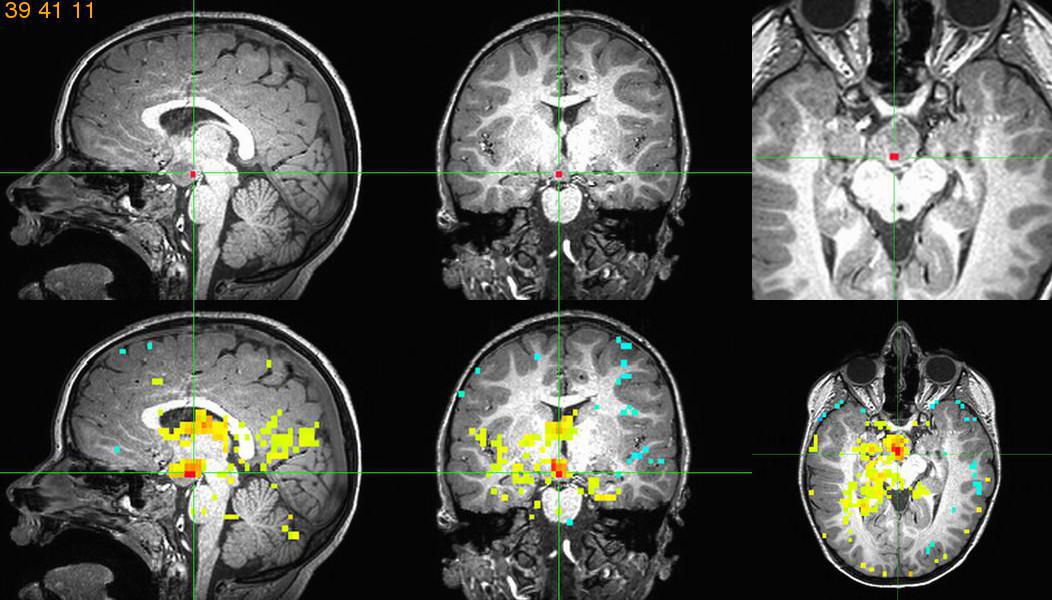 | 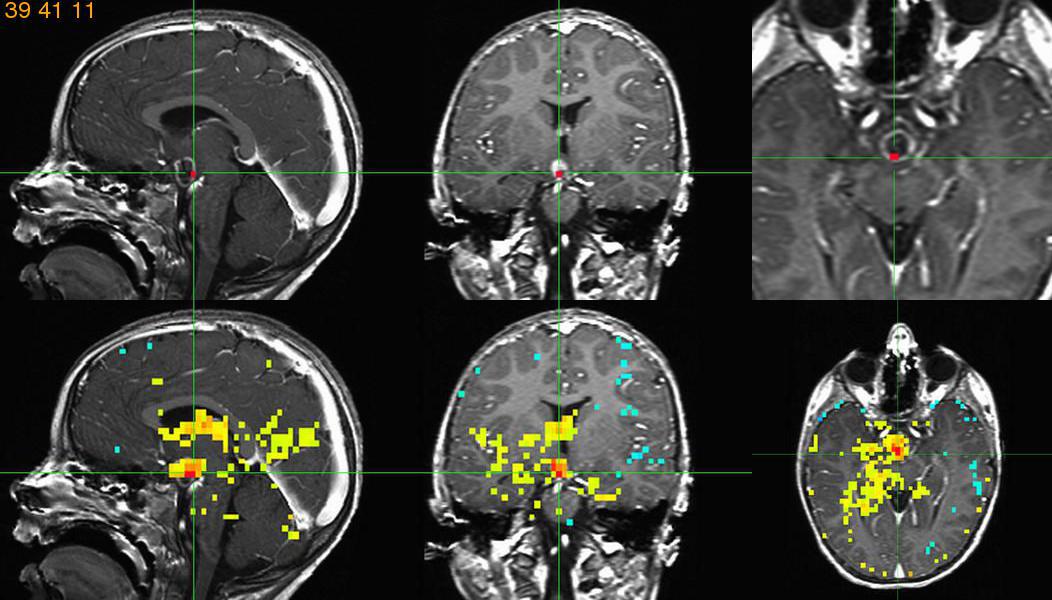  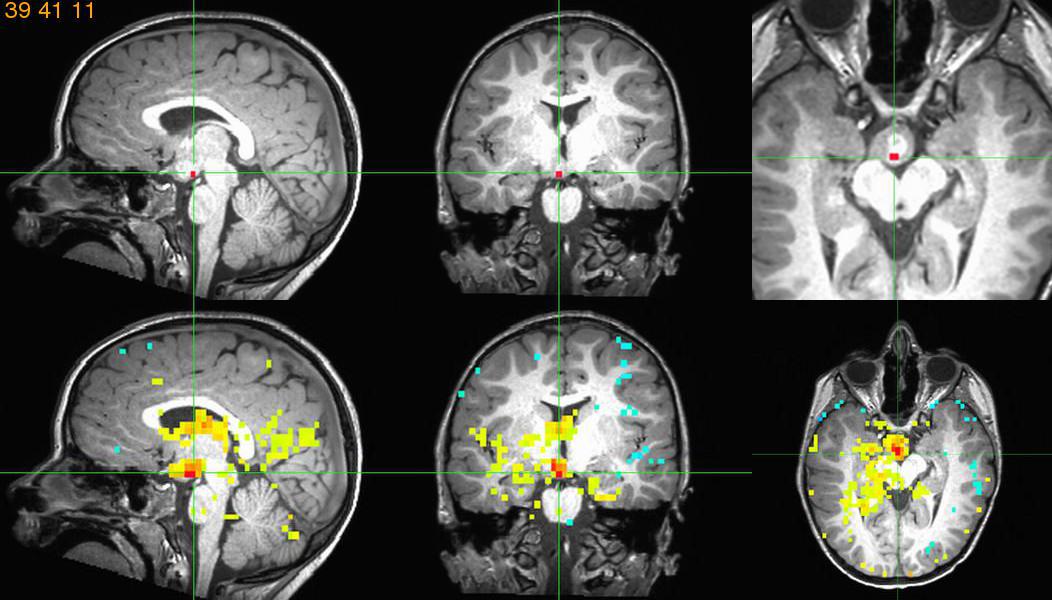 | 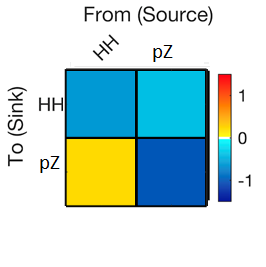 |
| P18-T14 | 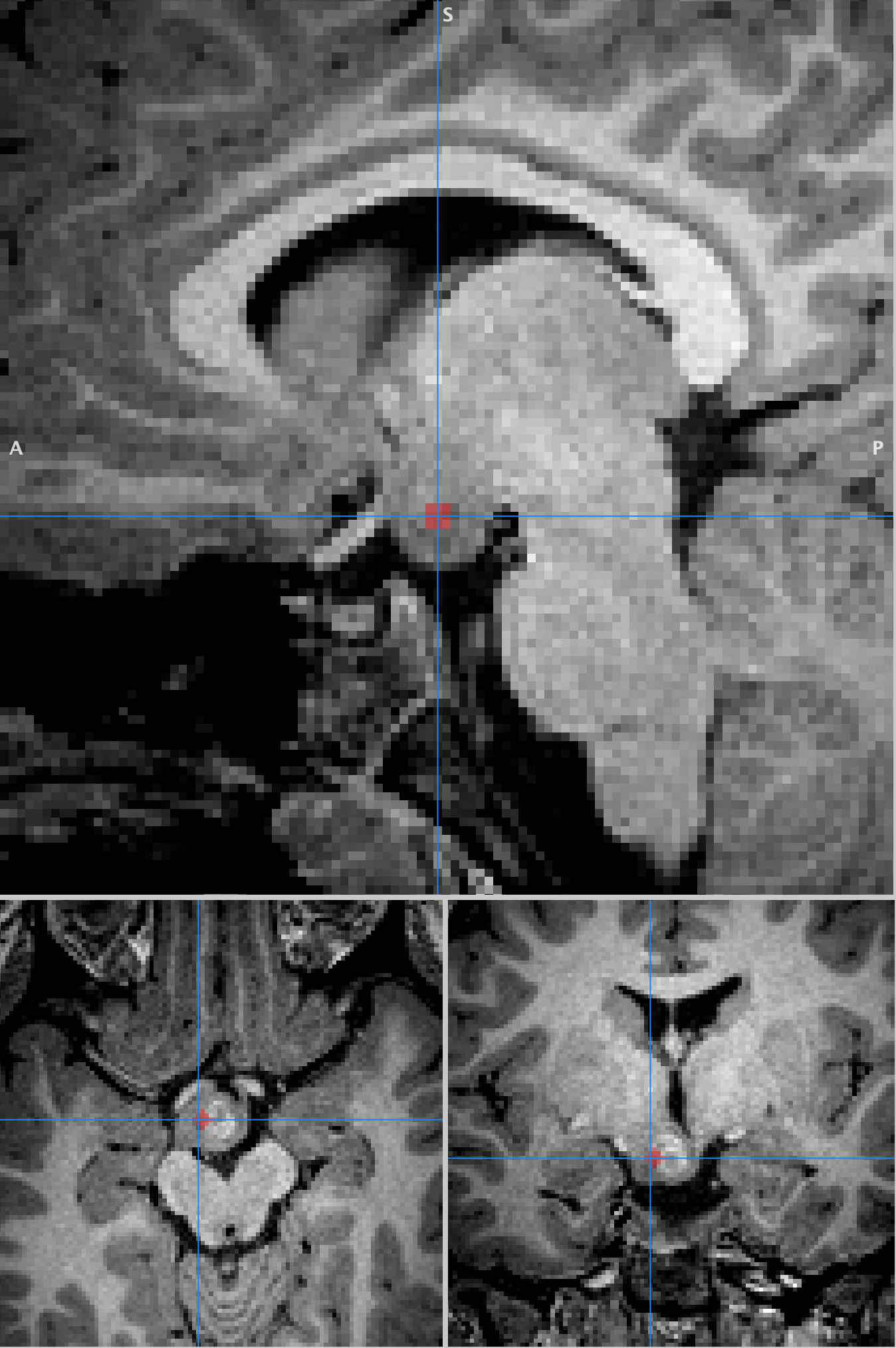 | 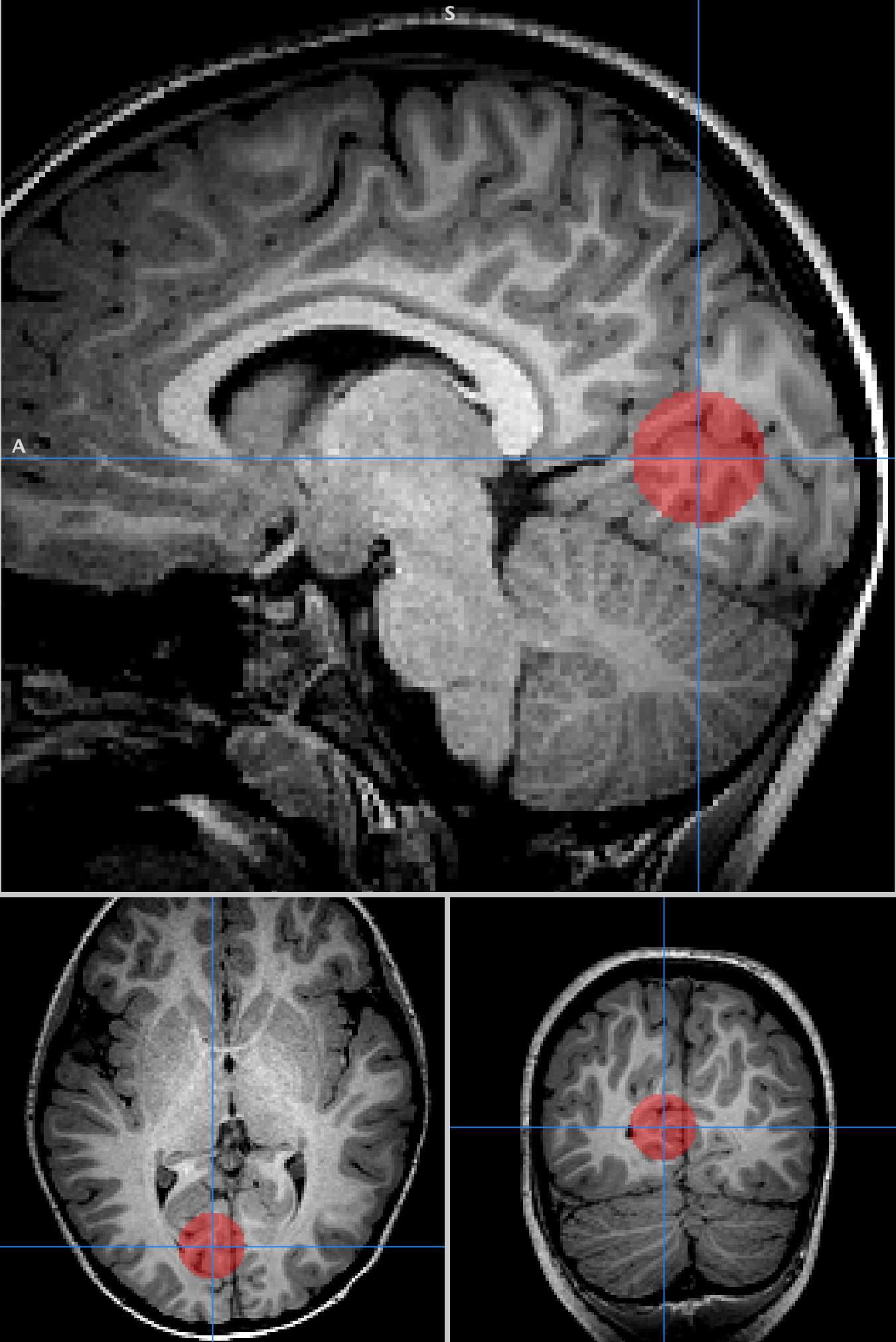 | 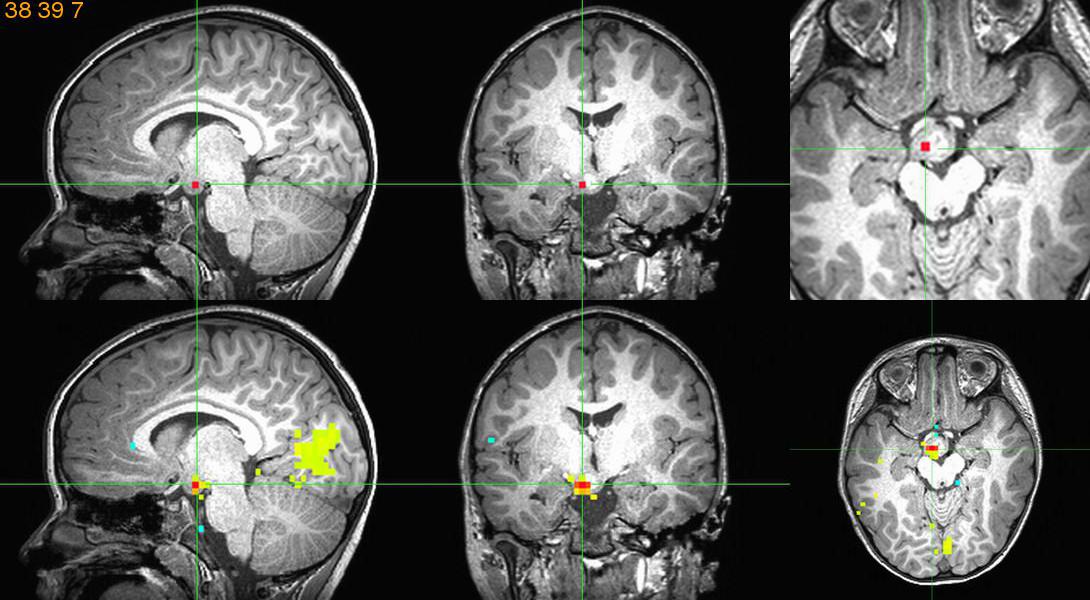 | 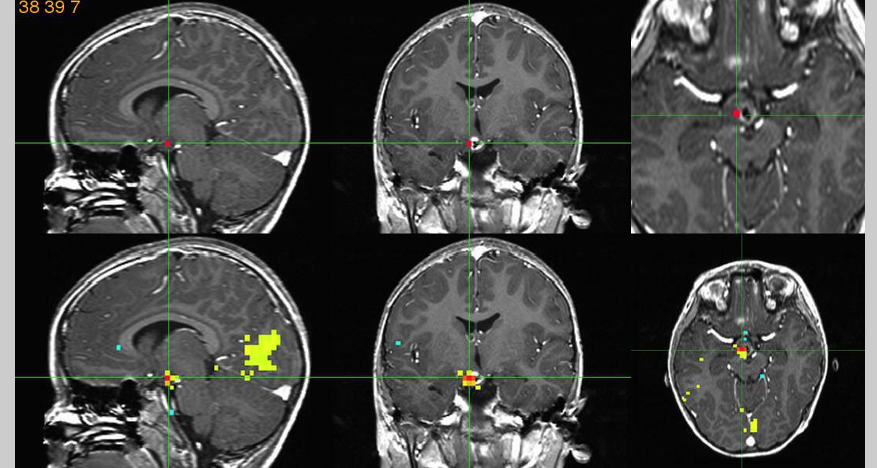 | 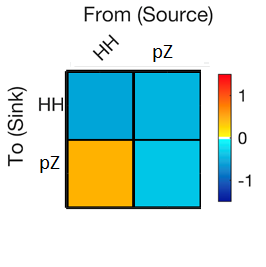 |
| P19-T16 | 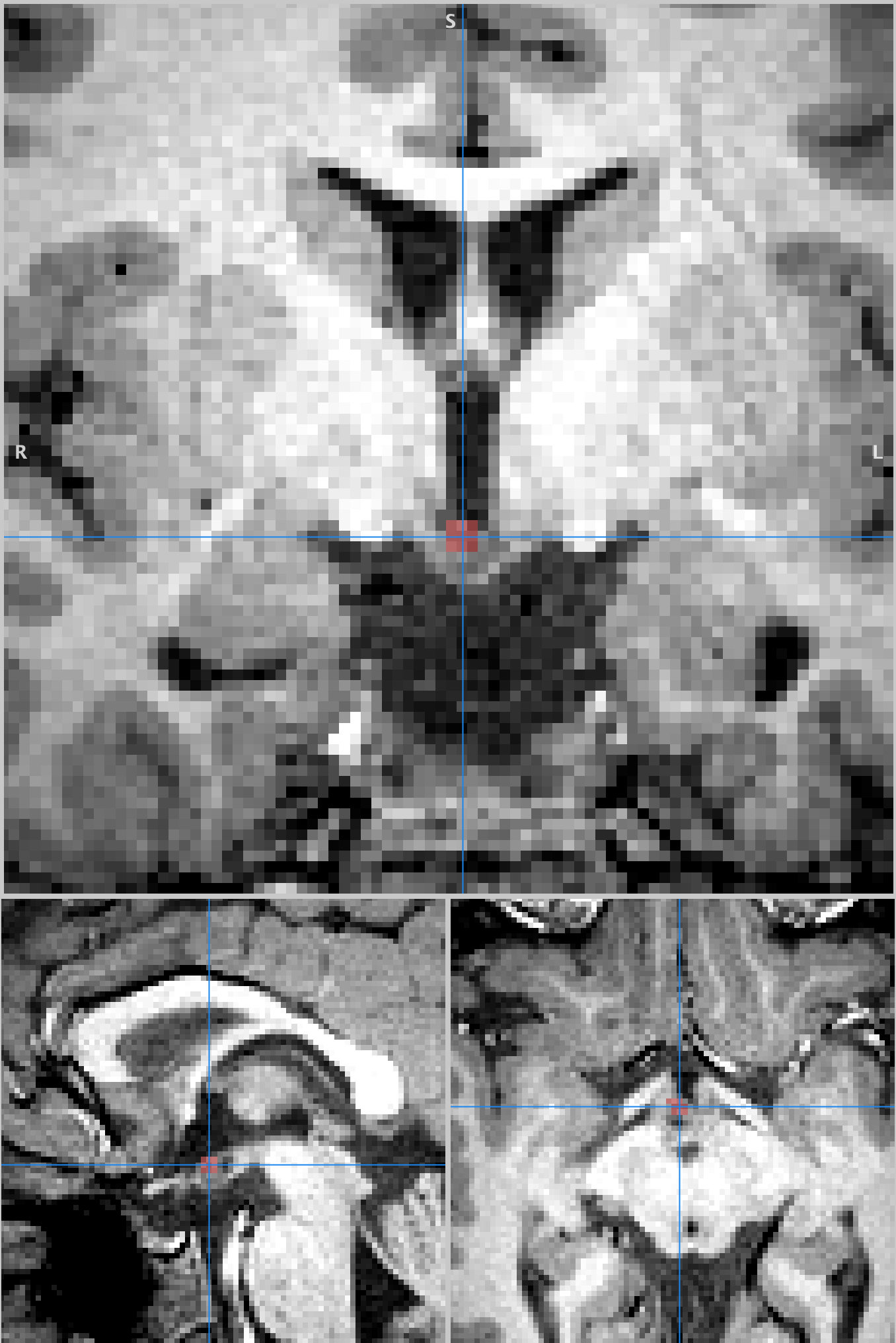 | 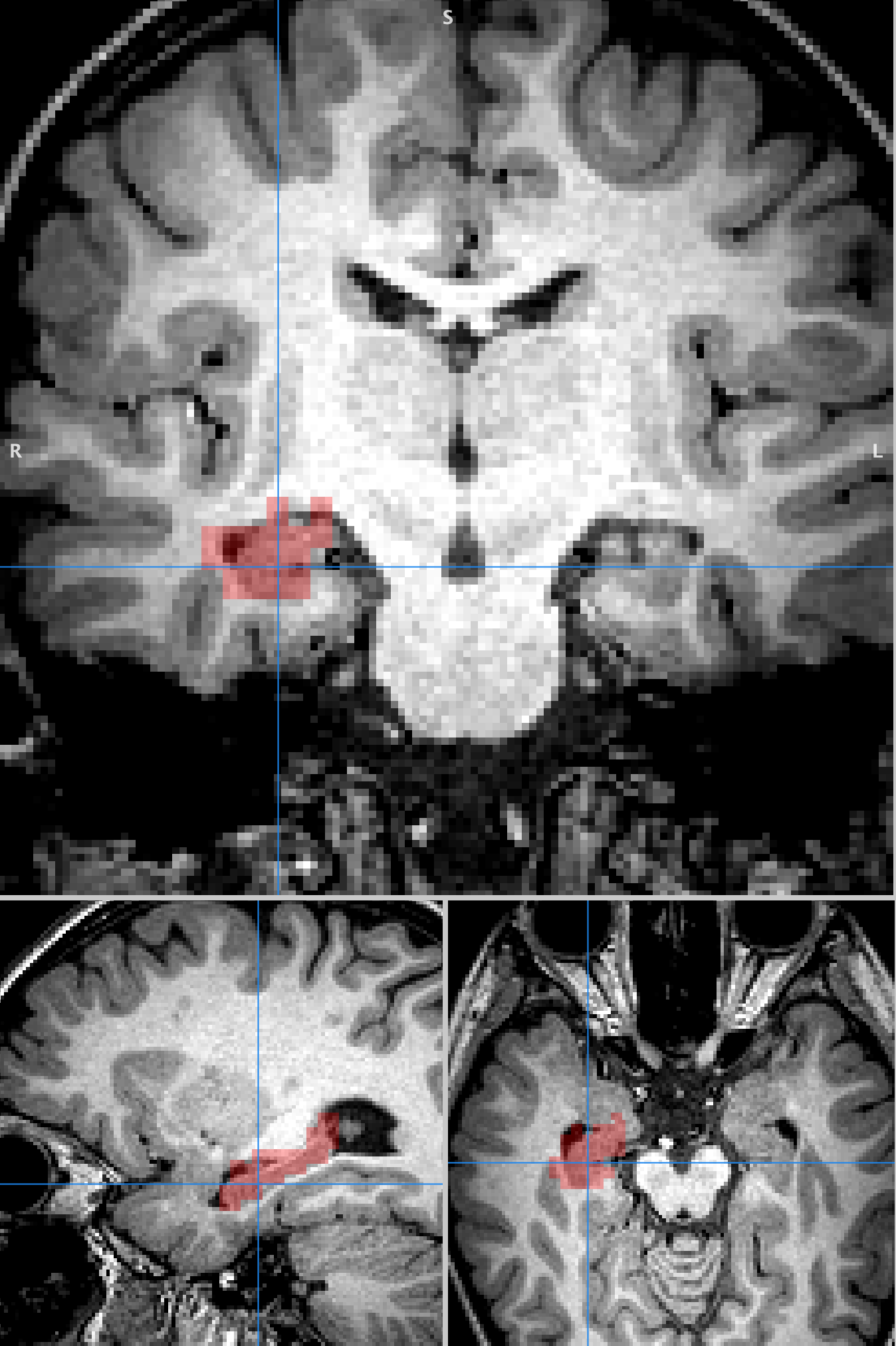 | 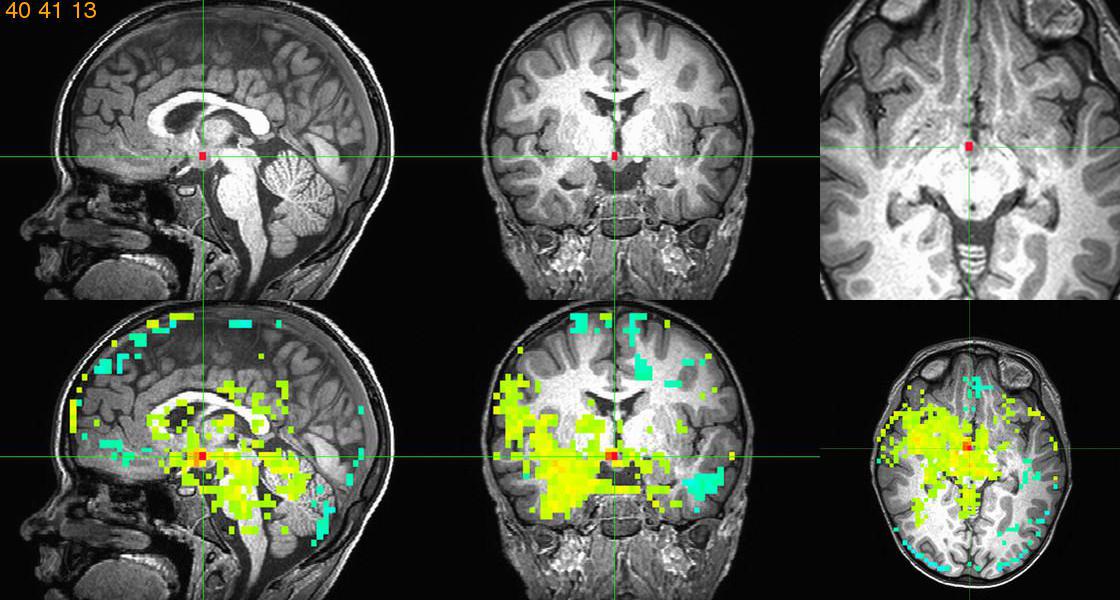 | 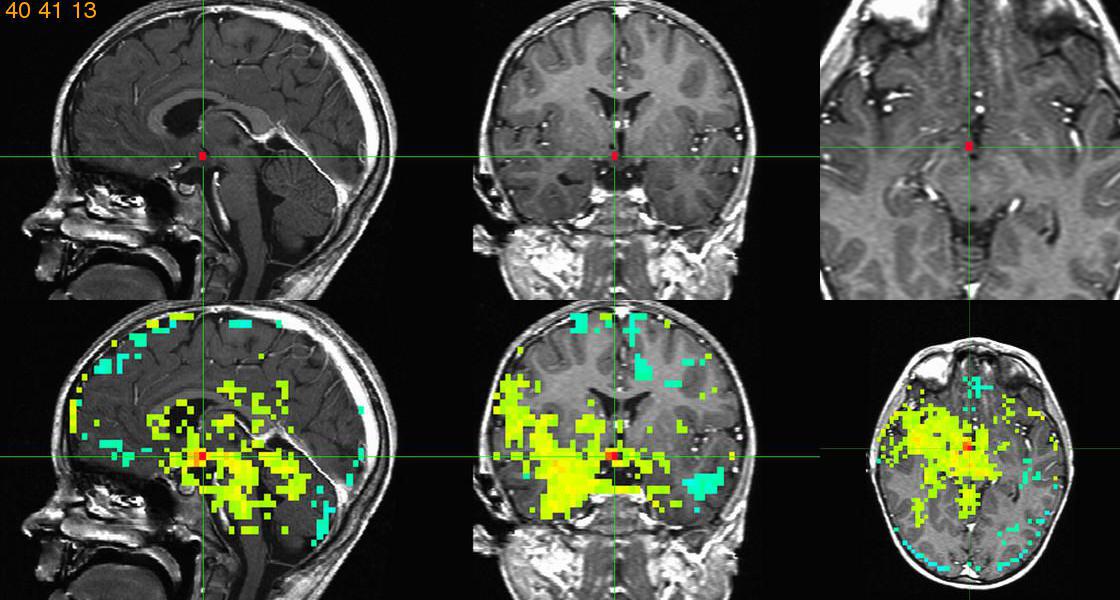 |  |
| P20-T17 |  |  |  |  |  |
| P21-T18 |  |  |  |  |  |
| P22-T20 |  |  |  |  |  |
| P23-T21 |  |  |  |  |  |
| P24-T22 |  |  |  |  |  |
| P25-T23 |  |  |  |  |  |
| P26-T24 |  |  |  |  |  |
| P27-T28 |  |  |  |  |  |
| P28-T29 |  |  |  |  |  |
| P29-T30 |  |  |  |  |  |
| P30-T31 |  |  |  |  |  |
| P31-T33 |  |  |  |  |  |
| P-32-T32 |  |  |  |  |  |
|  |  |  |  |  |  |
