# Supplementary material for: Discerning Seizure-Onset v. Propagation Zone: Pre-and-Post-Operative Resting-State fMRI Directionality and Boerwinkle Neuroplasticity Index
Source: Neuroimage Clin. 2022 May 28;35:103063. doi: 10.1016/j.nicl.2022.103063 (PMC9163994; doi:10.1016/j.nicl.2022.103063)
Supplement: Supplementary data 2 [file mmc2.docx]

**Table S2. Demographics, Seizure Frequency, and Engel Outcomes**

| Pre-op ID # | Post-op ID # | Age at scan (yr.) | Gender M=1  F=2 | Hand R=1, L=2, ND=3 | Pre-op seizure per month | Post-op seizure per month | Seizure improv count | Seizure improv % | Engel Class | Pre-op SOZ→pZ | Pre-op pZ→SOZ | Post-op  BNI ^a^ |
| --- | --- | --- | --- | --- | --- | --- | --- | --- | --- | --- | --- | --- |
| 1 | 44 | 7.6 | 1 | 1 | 180 | 0 | -180 | 100 | Ia | + ^TP^ | - ^TP^ | No Signal ^TN^ |
| 2 |  | 17.0 | 1 | 1 | 40 | 0 | -40 | 100 | Ia | + ^TP^ | - ^TP^ |  |
| 3 |  | 13.4 | 2 | 1 | 150 | 0 | -150 | 100 | Ia | + ^TP^ | - ^TP^ |  |
| 4 |  | 2.6 | 1 | 1 | 450 | 0 | -450 | 100 | Ia | + ^TP^ | - ^TP^ |  |
| 5 |  | 8.4 | 1 | 1 | 4 | 0 | -4 | 100 | Ia | + ^TP^ | - ^TP^ |  |
| 6 |  | 9.4 | 2 | 1 | 46 | 2 | -44 | 96 | Ib | + ^TP^ | - ^TP^ |  |
| 7 | 32 | 14.2 | 1 | 1 | 2 | 0 | -2 | 100 | Ia | + ^TP^ | ns ^FN^ | BNI>70% ^TN^ |
| 8 |  | 4.2 | 2 | 1 | 25 | 5 | -20 | 80 | Ib | + ^TP^ | - ^TP^ |  |
| 9 |  | 9.5 | 1 | 2 | 39 | 11 | -28 | 73 | Ib | - ^FP^ | + ^FP^ |  |
| 10 |  | 5.7 | 1 | 2 | 11 | 1 | -10 | 91 | Ib | + ^TP^ | - ^TP^ |  |
| 11 |  | 3.2 | 1 | 2 | 10 | 1 | -9 | 90 | Ib | + ^TP^ | - ^TP^ |  |
| 12 | 33 | 5.0 | 2 | 2 | 60 | 0 | -60 | 100 | Ia | + ^TP^ | - ^TP^ | No Signal ^TN^ |
| 13 |  | 3.6 | 2 | 1 | 14 | 0 | -14 | 100 | Ia | + ^TP^ | - ^TP^ |  |
| 14 |  | 3.8 | 2 | 1 | 53 | 14 | -39 | 73 | Ib | + ^TP^ | - ^TP^ |  |
| 15 |  | 11.5 | 2 | 1 | 18 | 0 | -18 | 100 | Ia | + ^TP^ | - ^TP^ |  |
| 16 | 34 | 18.3 | 2 | 1 | 10.5 | 0 | -10.5 | 100 | Ia | + ^TP^ | - ^TP^ | BNI<70% ^FP^ |
| 17 |  | 5.2 | 1 | 3 | 420 | 63 | -357 | 85 | Ib | + ^TP^ | - ^TP^ |  |
| 18 |  | 6.5 | 1 | 3 | 53 | 32 | -21 | 40 | Ib | + ^TP^ | - ^TP^ |  |
| 19 |  | 5.0 | 1 | 1 | 18 | 0 | -18 | 100 | Ia | + ^TP^ | - ^TP^ |  |
| 20 | 35 | 4.0 | 1 | 3 | 32 | 0 | -32 | 100 | Ia | + ^TP^ | - ^TP^ | BNI>70% ^TN^ |
| 21 | 36 | 3.0 | 1 | 2 | 123 | 0 | -123 | 100 | Ia | + ^TP^ | - ^TP^ | BNI>70% ^TN^ |
| 22 |  | 18.1 | 2 | 3 | 60 | 21 | -39 | 65 | Ib | + ^TP^ | - ^TP^ |  |
| 23 | 37 | 3.7 | 1 | 1 | 53 | 0 | -53 | 100 | Ia | + ^TP^ | - ^TP^ | BNI>70%^TN^ |
| 24 |  | 4.7 | 2 | 3 | 82 | 21 | -61 | 74 | Ib | + ^TP^ | - ^TP^ |  |
| 25 |  | 5.9 | 2 | 3 | 21 | 0 | -21 | 100 | Ia | + ^TP^ | - ^TP^ |  |
| 26 | 38 | 9.9 | 1 | 2 | 53 | 0 | -53 | 100 | Ia | ns ^FN^ | - ^TP^ | No Signal ^TN^ |
| 27 | 39 | 5.7 | 1 | 1 | 21 | 0 | -21 | 100 | Ia | ns ^FN^ | - ^TP^ | BNI > 70% ^TN^ |
| 28 | 40 | 2.1 | 1 | 3 | 15 | 0 | -15 | 100 | Ia | + ^TP^ | - ^TP^ | BNI > 70% ^TN^ |
| 29 |  | 18.2 | 1 | 1 | 420 | 0 | -420 | 100 | Ia | + ^TP^ | - ^TP^ |  |
| 30 | 41 | 4.5 | 1 | 2 | 140 | 0 | -140 | 100 | Ia | + ^TP^ | - ^TP^ | BNI > 70% ^TN^ |
| 31 | 43 | 15.7 | 1 | 3 | 21 | 0 | -21 | 100 | Ia | ns ^FN^ | ns ^FN^ | BNI < 70% ^FP^ |
| 45* | 42 | 2.3 | 1 | 3 | 17.5 | 0 | -17.5 | 100 | Ia | ns ^FN^ | ns ^FN^ | No Signal ^TN^ |

*Note.* Improv = improvement; L = left; ND = not determined; ns = detected signal not statistically significant; R = right; yr. = year; ( * ) patient not included in pre-operative analysis due to number of scan volumes available but included included in the pre-post analysis; ( + ) = excitatory; ( - ) = inhibitory; ^TP^ = true positive; ^TN^ = true negative; ^FP^ = false positive; ^FN^ = false negative.

^a^ Boerwinkle Neuroplasticity Index = [SOZ Recovery Index = ] + [pZ Recovery Index = ].

$\frac{[pre - post soz\to pZ]}{[pre\_soz\to pZ]}$ *x 100*

$\frac{[pre - post pZ\to Soz]}{[pre\_pZ\to SOZ]}$ *x 100*
